# Synthesis, Biological Evaluation, and In Silico Modeling of *N*-Substituted Quinoxaline-2-Carboxamides

**DOI:** 10.3390/ph14080768

**Published:** 2021-08-04

**Authors:** Ghada Bouz, Sarah Bouz, Ondřej Janďourek, Klára Konečná, Pavel Bárta, Jarmila Vinšová, Martin Doležal, Jan Zitko

**Affiliations:** Faculty of Pharmacy in Hradec Králové, Charles University, 50005 Hradec Králové, Czech Republic; sarah-bouz@hotmail.com (S.B.); jando6aa@faf.cuni.cz (O.J.); konecna@faf.cuni.cz (K.K.); pavel.barta@faf.cuni.cz (P.B.); vinsova@faf.cuni.cz (J.V.); dolezalm@faf.cuni.cz (M.D.)

**Keywords:** antimycobacterial, cytotoxicity, molecular docking, *Mycobacterium tuberculosis*, pyrazinamide, quinoxaline, tuberculosis

## Abstract

Despite the established treatment regimens, tuberculosis remains an alarming threat to public health according to WHO. Novel agents are needed to overcome the increasing rate of resistance and perhaps achieve eradication. As part of our long-term research on pyrazine derived compounds, we prepared a series of their ortho fused derivatives, *N*-phenyl- and *N*-benzyl quinoxaline-2-carboxamides, and evaluated their in vitro antimycobacterial activity. In vitro activity against *Mycobacterium tuberculosis* H37Ra (represented by minimum inhibitory concentration, MIC) ranged between 3.91–500 µg/mL, with most compounds having moderate to good activities (MIC < 15.625 µg/mL). The majority of the active compounds belonged to the *N*-benzyl group. In addition to antimycobacterial activity assessment, final compounds were screened for their in vitro cytotoxicity. *N*-(naphthalen-1-ylmethyl)quinoxaline-2-carboxamide (compound **29**) was identified as a potential antineoplastic agent with selective cytotoxicity against hepatic (HepG2), ovarian (SK-OV-3), and prostate (PC-3) cancer cells lines. Molecular docking showed that human DNA topoisomerase and vascular endothelial growth factor receptor could be potential targets for **29**.

## 1. Introduction

**Figure 1:**
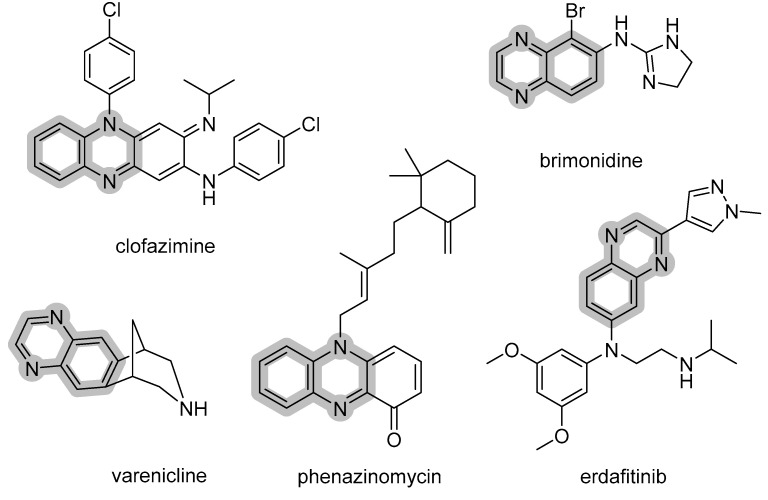
Examples of established drugs bearing a quinoxaline core in their chemical structures.

Quinoxaline is an attractive core in medicinal chemistry. Structure wise, quinoxaline is an isostere of quinoline (N for C) and naphthalene (2N for 2C), while it is a bioisostere of quinazoline, indole, benzimidazole, benzothiophene, and benzothiazole. Quinoxaline containing compounds can bind to various targets, which make them, in return, a privileged structure. Examples of marketed drugs bearing quinoxaline include the antileprotic clofazimine, the smoking cessation aid varenicline, the antitumor antibiotic phenazinomycin, the antitumor erdafitinib (inhibitor of fibroblast growth factor receptor, FGFR), and the α-adrenergic abrimonidine (refer to Figure 1).

**Figure 2:**
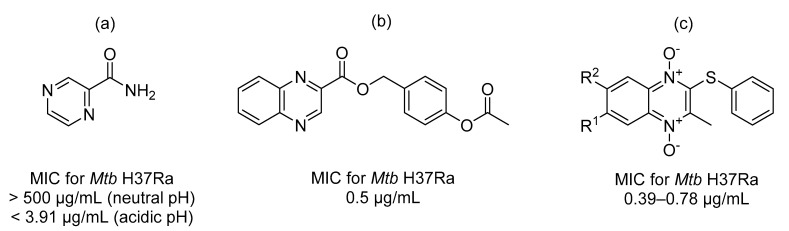
Chemical structures of (**a**) pyrazinamide; (**b**) 4-acetoxybenzyl quinoxaline-2-carboxylate; and (**c**) 3-methyl-2-(phenylthio)quinoxaline 1,4-dioxide derivatives.

The heterocyclic part of the quinoxaline nucleus is pyrazine. Pyrazinamide (Figure 2a) is a first-line antitubercular drug hydrolyzed by nicotinamidases to its active form pyrazinoic acid (pyrazine-2-carboxylic acid) in the cytoplasm [1]. Despite the availability of treatment plans for such an old infection, tuberculosis has not been eradicated yet and continues to be the leading cause of death from a single infectious agent, according to the WHO [2]. As an attempt to develop novel antituberculars, Seitz et al. prepared several derivatives of quinoxaline-2-carboxylic acids [3]. They identified one ester of quinoxaline-2-carboxylic acid, which acts as a prodrug with potent in vitro antitubercular activity against *Mycobacterium tuberculosis* H37Ra (*Mtb* H37Ra) (refer to Figure 2b). They suggested that the acetoxy group in the compound will be reduced to the free hydroxy group. Other quinoxaline-containing compounds with antitubercular activity against both drug-sensitive and drug-resistant strains are quinoxaline 1,4-dioxide and its derivatives [4,5]. The simple structure of quinoxaline 1,4-dioxide allows for several structural modifications to optimize activity and pharmacokinetic profile. Among all, derivatives of 3-methyl-2-(phenylthio)quinoxaline 1,4-dioxide possessed the best antimycobacterial activity [5] (refer to Figure 2c). Quinoxaline 1,4-dioxides also exert antiparasitic activity. The nature of the substituents (R^1^, R^2^) determines whether the compound will exhibit antimycobacterial or antiparasitic activity, as reviewed elsewhere [6].

**Figure 3:**
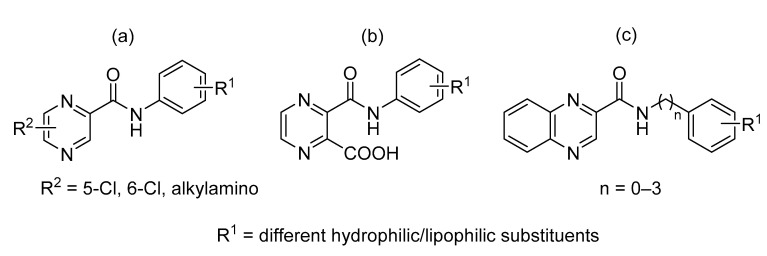
General structures of (**a**) our previously published compounds with favorable antitubercular activity; (**b**) 3-phenylcarbamoylpyrazine-2-carboxylic acid derivatives; and (**c**) title compounds.

Our research group is focused on developing novel antituberculars derived from pyrazinamide. Based on our previous studies of *N*-benzylpyrazine-2-carboxamides, we concluded that having a more lipophilic substituent on the pyrazine core such as 5-Cl, 6-Cl, or long alkylamino chains, favors in vitro antimycobacterial activity (refer to Figure 3a) [7,8,9]. Besides, in one of our publications, we suggested replacing the pyrazine core in a series of 3-phenylcarbamoylpyrazine-2-carboxylic acids (Figure 3b) with quinoxaline heterocycle as an attempt to improve the decaprenylphosphoryl-beta-D-ribose oxidase (DprE1) inhibition activity of such structures [10]. Therefore, in this paper, we report the design, synthesis, and biological activities of a series of quinoxaline-2-carboxamide derivatives (refer to Figure 3c for general structure). In contrast to esters prepared by Seitz et al. (Figure 2b), we prepared more stable amides of quinoxaline-2-carboxylic acids.

## 2. Results and Discussion

### 2.1. Chemistry

A series of quinoxaline-2-carboxylic acid derivatives was prepared by reacting quinoxaline-2-carboxylic acid activated with oxalyl chloride with the corresponding amines. Title compounds are mainly subdivided into two general structures differing in the length of the linker between the quinoxaline core and benzene core (addition of methylene -CH_2^-^_). In order to investigate the influence of the nature of the substituents (R) on biological activity, we selected various substituents ranging from hydrophilic to lipophilic. Depending on the availability of reagents, we managed to match nine pairs between the two structural types (R = H, 3-OH, 4-OH, 4-OCH_3_, 3-F, 2,4-diF, 2-Cl, 3-Cl, 3-CF_3_). We further extended the linker to ethylene (-C_2_H_4^-^_) and propylene (-C_3_H_6^-^_) for the non-substituted structures (R = H) to investigate such influence on biological activity. The final crude 33 compounds were purified by flash column chromatography. Final compounds were isolated as solids in moderate yields, usually ranging from 24% to 90% (after all purification steps) and characterized by ^1^H-NMR and ^13^C-NMR spectra, elemental analysis, IR spectra, and melting points. Obtained data were consistent with proposed structures. In the ^1^H-NMR spectra, the amidic hydrogen signal appeared as a singlet for *N*-phenyl derivatives (**1**–**14**) at 10.58–9.78 ppm (in CDCl_3_) or at 11.04–9.45 ppm (in DMSO-*d_6_*) and as a triplet with *J* = 6.6–6.0 HZ for the rest of the compounds at 9.55 ppm (in CDCl_3_) or at 9.81–8.66 ppm (in DMSO-*d_6_*). In the ^13^C-NMR spectra, the amidic carbon signal appeared at 164.07–160.64 ppm (in CDCl_3_) or 168.05–161.52 ppm (in DMSO-*d_6_*). All prepared compounds passed PAINS and Aggregators screening using ZINC15 utility (http://zinc15.docking.org/patterns/home; accessed on 5 March 2021).

### 2.2. Evaluation of Biological Activities

#### 2.2.1. In Vitro Antimycobacterial Activity

Title compounds were evaluated for their antitubercular activity against *Mtb* H37Ra, *M. smegmatis* (*M. smeg*), and *M. aurum* by the Microplate Alamar Blue Assay (MABA) [11]. According to the literature, minimum inhibitory concentration (MIC) values against *Mtb* H37Ra (avirulent strain) are at least qualitatively equal to MIC values against the standard virulent strain of *Mtb* H37Rv [12]. Therefore, the employment of avirulent strain *Mtb* H37Ra represents an adequate surrogate for the highly pathogenic *Mtb* H37Rv. *M. smegmatis* and *M. aurum* are fast-growing mycobacteria that cause infection in immunocompromised patients. At the same time, these two mycobacterial species are surrogate organisms, having a cell wall structure and resistance profile similar to *Mtb* H37Rv [13]. The cytotoxicity of the title compounds was assessed on the HepG2 liver cancer cell line. For active compounds against *Mtb* H37Ra (those with MIC values < 62.5 µg/mL), we calculated their activity in micro molar (µM) and selectivity index (SI) values to evaluate the selectivity of such compounds toward mycobacteria. SI values were calculated by dividing the IC_50_ against HepG2 by the MIC in µM against *Mtb* H37Ra. SI value above 10 is favorable as it indicates that the compound can be used at concentrations 10 times greater than its MIC without exerting toxicity. Results are summarized in Table 1. Apart from anti-microbial activity, literature search showed that compounds **1**, **5**, **6**, **13**, and **15** were previously evaluated as 5-HT_3_ receptor antagonists for the management of depression [14]. Compound **32** was evaluated as a G-protein-coupled metabotropic receptor (mGluR1) antagonist for the treatment of chronic pain [15].

Regarding antimycobacterial activity against *Mtb* H37Ra, we found that compounds with methylene bridge (*N*-benzyl derivatives; **15**–**31**) in general had better activity than *N*-phenyl derivatives (**1**–**14**). This is prominent with the two most active compounds being *N*-benzyl derivatives while their corresponding *N*-phenyl derivatives are inactive; R = 4-OCH_3_ [**20** (MIC = 3.91 µg/mL) vs. **5** (MIC ≥ 125 µg/mL)] and R = 3-CF_3_ [**28** (MIC = 3.91 µg/mL) vs. **14** (MIC ≥ 500 µg/mL)]. Among the nine matching pairs, *N*-benzyl derivatives had superior activities in seven pairs (R = H, 4-OH, 4-OCH_3_, 3-F, 2-Cl, 3-Cl, 3-CF_3_). Extension in the amidic bridge from methylene to ethylene (**32**) negatively affected the activity, while extending the bridge to propylene (**33**) preserved the antitubercular activity of compound **15** on *Mtb* H37Ra. As for the substituents (R), we found that there was no correlation between lipophilicity and activity. However, we noticed an influence of the position of the substituents, especially in compounds with a di-substituted benzene ring. Compounds with di-substitution at positions 2 and 4 were inactive, while those with 2,5 and 3,4 di-substitutions were active (**30** with R = 2,4-diCl had MIC*_Mtb_*_H37Ra_ ≥ 500 µg/mL, while **31** with R = 3, 4-diCl had MIC*_Mtb_*_H37Ra_ = 7.81 µg/mL). One interesting observation was that compound **6** bearing a cyclohexyl core was active against *Mtb* H37Ra (MIC = 31.25 µg/mL), while compound **1** with benzene was inactive (MIC ≥ 500).

In order to study the contribution of the quinoxaline core, we compared the antitubercular activity on *Mtb* H37Ra between the title compounds (we selected two of the most active with MIC = 3.91 µg/mL) and corresponding derivatives (the same substituent R) of 3-phenylcarbamoylpyrazine-2-carboxylic acids (Figure 2b), which were originally designed as DprE1 inhibitors (refer to Table 2). Published compounds in Semelkova et al. [10] were originally evaluated against the virulent strain *Mtb* H37Rv, therefore, we retested them on the avirulent strain *Mtb* H37Ra. Again, we found that *N*-benzyl-quinoxaline-2-carboxamides exerted superior activity.

As for antitubercular activity on *M. smegmatis*, compounds **6** (MIC = 31.25 µg/mL; 122.4 µM), **7** (MIC = 62.5 µg/mL; 233.8 µM), **16** (MIC = 62.5 µg/mL; 237.4 µM), and **26** (MIC = 31.25 µg/mL; 105 µM) exerted mild activity. Only compound **26** was active against *M. aurum* (MIC = 31.25 µg/mL; 105 µM), making it the compound with the broadest spectrum of activity.

#### 2.2.2. In Vitro Cytotoxicity

In vitro model of the human hepatocellular carcinoma (HepG2 cell line) was used to assess the cytotoxicity of the presented compounds. This model was chosen since the combinatorial multi-drug anti-TB regimen is known to carry the risk of hepatotoxicity [17]. Results are presented as the inhibitory concentration, which reduces the viability of the cell population to 50% of the maximum viability (IC_50_) and are calculated by non-linear regression from a semilogarithmic plot of incubation concentration versus percentage of the absorbance relative to the untreated controls. A number of compounds precipitated in the culture medium at higher concentrations, which made the determination of their absolute IC_50_ and consequently SI values unfeasible. Based on the obtained results presented in Table 1, just like antitubercular activity, cytotoxicity does not correlate with lipophilicity. For the most active compounds **18**, **20**, and **28**, we found that selectivity index (SI, preferably >10) favored compounds **18** (SI = 82.6) and **20** (SI = 16.1) over compound **28** (SI = 4.12). HepG2 IC_50_ curves of selected compounds can be found in Figure S1 in Supplementary Material S2.

Compound **29** was the most cytotoxic compound against HepG2 cells (IC_50_ = 37.3 µM), and interestingly was not active against the mycobacterial test strains. Therefore, compound **29** was also screened against eight bacterial (four Gram-positive and four Gram-negative) and eight fungal strains, where the activity against these agents was not revealed (refer to Table 3 for full biological activity profile). This selective cytotoxicity prompted further investigation of the antineoplastic properties of compound **29**. We first ensured that compound **29** was nontoxic for non-cancerous human cells by evaluating its toxicity against the kidney cortex proximal tubule HK-2 cell line. Then, we screened compound **29** against the human epithelial kidney cancer A498 cell line, human prostate cancer PC-3 cell line, human ovarian cancer SK-OV-3 cell line, and human glioblastoma U-87 MG cell line. Compound **29** was selectively toxic against the HepG2 (IC_50_ = 37.3 µM), PC-3 (IC_50_ = 68.5 µM), and SK-OV-3 (IC_50_ = 49.6 µM) cell lines. Finally, as an attempt to identify the molecular target for compound **29**, we docked the molecule to all targets mentioned in a review on anticancer quinoxalines by Kaushal et al. [18]. Results of the docking experiments are discussed in the following Section 2.3.2. 

### 2.3. In Silico Modeling - Molecular Docking Studies

#### 2.3.1. Exploring the Mechanism of Antimycobacterial Activity by In Silico Methods

**Figure 4:**
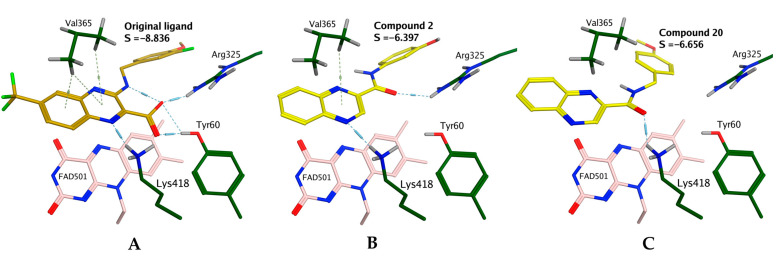
Results of molecular docking into mycobacterial decaprenylphosphoryl-beta-D-ribose oxidase (DprE1; pdb id: 4P8N). (**A**)—Original inhibitor redocked; (**B**)—Compound **2** as a representative of *N*-phenyl title compounds; (**C**)—Compound **20** as a representative of *N*-benzyl title compounds.

We used a simple molecular docking study to evaluate the potential of our title quinoxaline derivatives toward the inhibition of mycobacterial DprE1. Inhibition of DprE1 as a possible mechanism of action (MoA) was suggested based on the structural similarity to the confirmed quinoxaline-based inhibitor (see Figure 4, panel A). Based on the observed whole-cell antimycobacterial activity (Table 1), we chose compound **2** as a representative of the *N*-phenyl subseries (**1**–**14**, excl. **6**) and compound **20** as a representative of the *N*-benzyl series (**15**–**31**). These prototypic compounds were docked into mycobacterial DprE1 (pdb id: 4P8N). For results, see Figure 4. The original ligand (confirmed quinoxaline-based inhibitor) was redocked with RMSD = 0.458 Å and Score = −8.836. The original ligand (both in crystallographic and redocked pose) was stabilized by strong ionic interaction between Arg325 and carboxylate anion of the inhibitor, accompanied by two H-bonds (Tyr60 to carbonyl oxygen, Lys418 to N-1 of the quinoxaline core) and by hydrophobic interactions to the aromatic system of the quinoxaline core (arene–H interactions to Val365, π–π stacking interactions with FAD cofactor). The best pose for compound **2** (Figure 4, panel B, S = −6.397) retained the hydrophobic interactions to the quinoxaline core and the H-bond between Lys418 and N-1. The interactions of the missing carboxylic moiety (in comparison to the confirmed inhibitor) were partially substituted with the interaction of Arg325 to the carbonyl oxygen of the carboxamidic linker. However, the strong ionic interaction is missing, which is reflected by the significantly worse docking score. In compound **20**, the extended linker (extra methylene group) caused the deviation in the position of the quinoxaline core, which led to the loss of interaction with Arg325. This unfavorable shift was observed for all docked poses of title compounds with the extended linker (**15**–**31**).

Our title compounds are missing the important ionic interaction of the confirmed DprE1 inhibitor. Furthermore, the *N*-benzyl derivatives, which had higher in vitro antimycobacterial activity, exerted fewer interactions to the target in comparison with *N*-phenyl derivatives. Therefore, we do not consider inhibition of DprE1 as a probable MoA of our active quinoxaline derivatives. The replacement of the benzene core in series with the quinoxaline core improved the antitubercular activity on *Mtb* H37Ra, but not through improving the affinity of such structures to DprE1.

#### 2.3.2. Exploring the Mechanism of In Vitro Cytotoxicity by in Silico Methods

The molecular mechanisms of the cytotoxic (anticancer) activity of compounds based on the quinoxaline core have recently been reviewed [18]. Quinoxaline derivatives can act as inhibitors of angiogenesis, protein kinases inhibitors, inhibitors of topoisomerase I and/or II, DNA binding agents, antitubulin agents, inhibitors of Ras proteins or inhibitors of farnesyl transferase [18]. We chose our most cytotoxic quinoxaline derivative **29** (HepG2 IC_50_ = 37.3 µM) and determined its cytotoxicity profile in various cell lines (both cancerous and non-cancerous), see Table 3 for results. Compound **29** became our model compound for a pivotal in silico study of potential molecular mechanisms of its cytotoxic effects.

**Figure 5:**
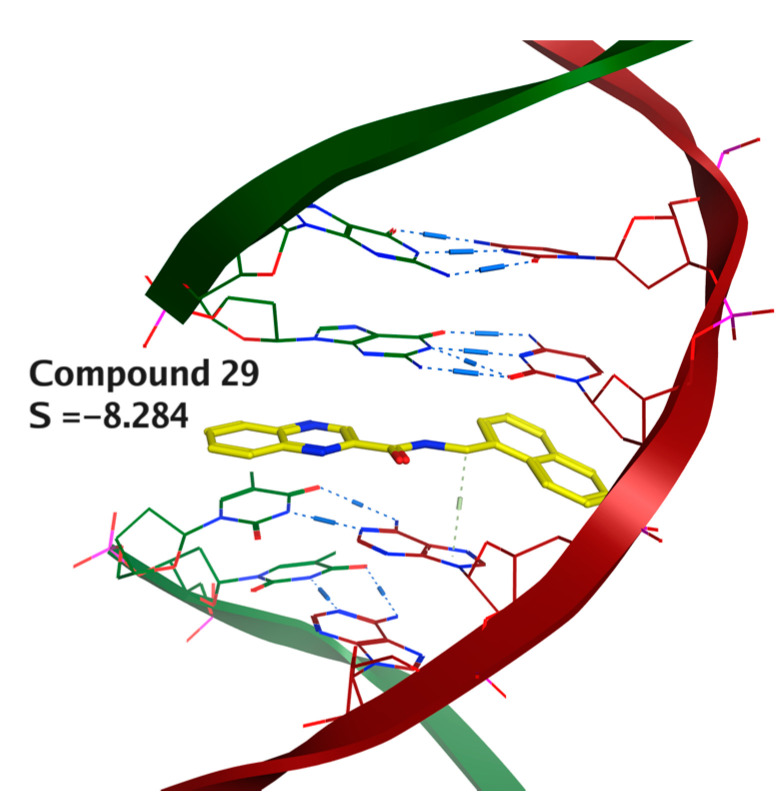
Intercalating properties of compound **29** predicted by molecular docking into the complex of human DNA topoisomerase with DNA (topoisomerase not depicted).

In the first approach, we performed molecular docking of compound **29** into human targets selected based on the review article above-mentioned [18]. The selection from the PDB database was made manually, considering the structure quality criteria and structural similarity of the co-crystalized ligand to compound **29**. The full list of studied targets may be found in Table S1 in Supplementary Material S3. The target for which we obtained the best docking score was the complex of human topoisomerase I with DNA (pdb id: 1K4T), and in Figure 5, we show that the planar aromatic rings of **29** are favorable for intercalation into the DNA in a similar manner as the original ligand of topotecan, which is an intercalating topoisomerase I inhibitor in clinical practice.

**Figure 6:**
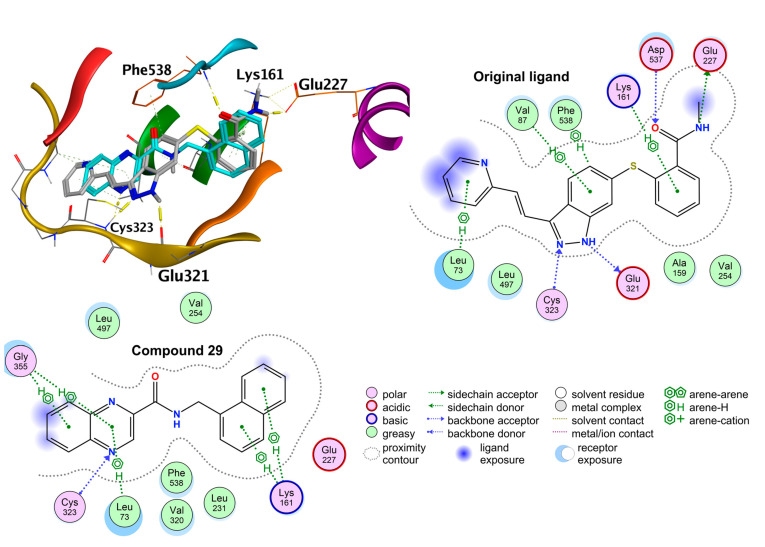
Results of the molecular docking of **29** (turquoise carbons) into vascular endothelial growth factor receptor 2 (VEGFR2; pdb id: 4AG8) in an overlay with the original ligand (grey carbons).

Many of the known anticancer targets of quinoxaline derivatives are various protein kinases [18]. Therefore, as a second approach, we used a curated database of protein kinases distributed with the MOE v2020.09 (Molecular Operating Environment) software package as a source of potential targets. From this database, we chose the structures of human kinases, which contained a ligand with structural similarity to compound **29** (MACCS Structural Keys (BitPacked), Tanimoto index ≥ 0.6). This search yielded 40 structures with unique PDB codes. The results of induced-fit molecular docking into these structures are summarized in Table S2 in Supplementary Material S3. In brief, the best scoring kinases were the kinase domains of vascular endothelial growth factor receptor 2 (VEGFR2) and VEGFR1. Therefore, as the next step, we extracted all human VEGFR2 structures from the MOE Kinase Database with resolution ≤ 2.5 Å, yielding 33 structures with unique PDB code (refer to Supplementary Material S3 for the list) and performed the induced-fit docking of **29** into these. In the top 10 list of best scoring structures (one best pose of the ligand per receptor), we observed that the absolutely best scoring pose of ligand **29** repeated five times (see Supplementary Material S3, Table S3). In Figure 6, we present this candidate binding mode of **29** to VEGFR2. Original ligand redocked with S = −10.284, RMSD = 0.432, and ligand efficiency (expressed as the score divided by number of heavy atoms) LE = −0.367, and compound **29** was docked with S = −8.074 and LE = −0.336. As seen from Figure 6, compound **29** was able to exert several arene–H interactions in a similar manner to the original ligand. Moreover, the quinoxaline nitrogen N-4 was an accepting H-bond from Cys323 similarly to N-2 of the indazole of the original ligand. We also intended to perform docking to human VEGFR1; however, only structures of low resolution could be found in the PDB database.

To sum up, our in silico efforts to suggest the possible mechanism of cytotoxicity of compound **29** confirmed some of the previously reported targets of cytotoxic quinolones, namely topoisomerase/DNA and various protein kinases. Our in silico modeling especially brought attention to the kinase domain of the human receptor for vascular endothelial growth factor (VEGFR). Interestingly, it was reported that interaction with VEGFR can trigger apoptosis in the HepG2 cell line [19]. This is consistent with the HepG2 cytotoxicity observed in our in vitro experiments. Furthermore, HepG2 [20], PC-3, and SK-OV-3 [21] cell lines were proven to express VEGFR2, while cell lines U-87 MG [22] and A498 [23] expressed VEGFR1. This fact may explain the selective toxicity of compound **29** toward the HepG2, PC-3, and SK-OV-3 cell lines.

## 3. Materials and Methods

### 3.1. General Information

All reagents and solvents (unless stated otherwise) were purchased from Sigma-Aldrich (Schnelldorf, Germany) and used without further purification. Reaction progress and purity of products were monitored using Silica 60 F_254_ TLC plates (Merck, Darmstadt, Germany). Flash chromatography of the final compounds was performed on a puriFlash XS420+ (Interchim, Montluçon, France) with original columns (spherical silica, 30 µm) provided by the same company. The mobile phase was ethyl acetate (EtOAc) in hexane (Hex), gradient elution 0–100%, and detection was performed by UV-VIS detector at 254 nm and 280 nm. The NMR spectra were recorded on a Varian VNMR S500 (Varian, Palo Alto, CA, USA) at 500 MHz for ^1^H and 126 MHz for ^13^C. The spectra of compounds **5**–**13**, **16**, and **17** were measured on Jeol JNM-ECZ600R at 600 MHz for ^1^H and 151 MHz for ^13^C. The spectra were recorded in DMSO-*d_6_* or CDCl_3_ at ambient temperature. The chemical shifts reported as δ values in ppm are indirectly referenced to tetramethylsilane (TMS) via the solvent signal (2.49 for ^1^H and 39.7 for ^13^C in DMSO-*d_6_*; 7.27 for ^1^H and 77.0 for ^13^C in CDCl_3_). IR spectra were recorded on a NICOLET 6700 FT-IR spectrophotometer (Nicolet, Madison, WI, USA) using the ATR-Ge method. Elemental analysis was done on a Vario MICRO cube Element Analyzer (Elementar Analysensysteme, Hanau, Germany) with values given as a percentage. Yields are given in percentage and refer to the amount of pure product after all purification steps. Melting points were determined in open capillary on a Stuart SMP30 melting point apparatus (Bibby Scientific Limited, Staffordshire, UK) and are uncorrected. Log*P* values were calculated using ChemDraw v18.1. (PerkinElmer Informatics, Waltham, MA, USA).

### 3.2. Chemistry

Final compounds were prepared from quinoxaline-2-carboxylic acid and corresponding amines by amidation using oxalyl chloride as an activating agent. Quinoxaline-2-carboxylic acid (2 mmol, 348 mg) was dissolved in 15 mL of anhydrous DCM in a 50 mL Erlenmeyer flask with stirring. In a separate 10 mL Erlenmeyer flask, oxalyl chloride (2 mmol, 254 mg) was mixed with 3 mL of anhydrous DCM, and then oxalyl chloride solution was added to the first flask. Two drops of DMF were then added to the reaction mixture. The mixture was covered with parafilm and let to stir at room temperature for 30 min (until no more effervescence by evolving gas, full activation of acid). Meanwhile, 2 mmol of the corresponding amine was added to 20 mL of anhydrous DCM with stirring. Pyridine (4.5 mmol, 356 mg) was then added to the amine solution, and the mixture was covered with parafilm and put into an ice bath. After 15 min, the content of the first Erlenmeyer flask (acyl chloride) was slowly added (dropwise) to the amine mixture while stirring in the ice bath for 30 min and the reaction was left to stir at room temperature overnight. The organic layer (DCM) was washed with equal volume (40 mL) of dilute HCl (5%), then two times with distilled water (2 × 40 mL) and brine (1 × 20 mL). The organic layer was dried over anhydrous MgSO_4_, adsorbed to silica, and purified with flash chromatography using gradient elution 0 to 100% EtOAc in Hex.

### 3.3. Analytical Data

Refer to Supplementary Material S1 for the analytical data of prepared compounds including physical description, melting point, yields, ^1^H-NMR, ^13^C-NMR, IR, and elemental analysis. Copies of ^1^H-NMR and ^13^C-NMR for title compounds are also enclosed.

### 3.4. Evaluation of Biological Activities

Full description of the used methodologies can be found in Supplementary Material S2.

#### 3.4.1. In Vitro Antimycobacterial Activity

Testing was performed by MABA [11] where the results were expressed as MIC in µg/mL in comparison with isoniazid (INH), rifampicin (RFM), and ciprofloxacin (CPX) as standards.

#### 3.4.2. In Vitro Antibacterial Activity

Microdilution broth method was performed according to EUCAST recommendations, with slight modifications. Results were expressed as MIC compared to gentamicin (GNT) and CPX as standards.

#### 3.4.3. In Vitro Antifungal Activity

Microdilution broth method was performed according to EUCAST recommendations, with slight modifications. Results were expressed as MIC compared to the standards amphotericin B (AmB) and voriconazole (VRC).

#### 3.4.4. In Vitro Cytotoxicity

The cytotoxicity of title compounds was measured using the standard human hepatocellular carcinoma HepG2 cell line. The used CellTiter 96 assay is based on the reduction of tetrazolium dye MTS in living cells to formazan, which is then determined colorimetrically. Further cytotoxicity evaluations for the selected compound **29** were performed on the kidney cortex proximal tubule HK-2 cell line (non-cancerous), human epithelial kidney cancer A498 cell line, human prostate cancer PC-3 cell line, human ovarian cancer SK-OV-3 cell line, and human glioblastoma U-87 MG cell line.

### 3.5. In Silico Modeling—Molecular Docking Studies

In silico calculations were performed in Molecular Operating Environment (MOE), 2020.09 (Chemical Computing Group ULC, Montreal, QC, Canada). A full description of the methodology used can be found in Supplementary Material S3.

## 4. Conclusions

To conclude, based on our experience with pyrazinecarboxamides and their antimycobacterial activity, we intended to study the influence of the quinoxaline core on such activity. Therefore, we prepared 33 derivatives of quinoxaline carboxamide, out of which 14 compounds were *N*-phenyl derivatives (compounds **1**–**14**, excl. **6**) and 17 compounds were *N*-benzyl derivatives (compounds **15**–**31**). To investigate the effect of extending the length of the carboxamidic bridge, we also prepared *N*-phenethylquinoxaline-2-carboxamide (two carbon bridge, **32**) and *N*-phenylpropylquinoxaline-2-carboxamide (three carbon bridge, **33**). We found that *N*-phenyl derivatives exerted the best activity against *Mtb* H37Ra and that the lipophilic nature of the substituent has no direct influence on the activity. *N*-phenyl derivatives in this paper had better activity against *Mtb* H37Ra than their corresponding 3-phenylcarbamoylpyrazine-2-carboxylic acid derivatives (previously published by our group as potential inhibitors of DprE1) bearing the same substituent R on the benzene ring. However, molecular docking studies showed that the improved activity was probably not due to better interaction with the DprE1 enzyme, suggesting that title compounds exert their antitubercular activity via a different, unknown mechanism. Through in vitro cytotoxicity screening in HepG2 liver cancer cell line, we identified compound **29** as a potential selective cytotoxic agent (inactive against mycobacterial strains). We further explored the cytotoxic nature of compound **29**, and we found that it is nontoxic against noncancerous human cell line, nontoxic against bacterial or fungal strains, nontoxic against epithelial kidney cancer A498 cell line and human glioblastoma U-87 MG, but was toxic against the human prostate cancer PC-3 cell line and human ovarian cancer SK-OV-3 cell line. This selective toxicity against liver, prostate, and ovarian cancer cell lines prompted a further investigation on the mechanism of action. We performed molecular docking studies during which we identified human DNA topoisomerase and vascular endothelial growth factor (VEGFR2) as potential targets for compound **29**. VEGFR2 is linked to apoptosis in the HepG2 cell line. The other two cell lines toward which compound **29** was found to be cytotoxic also express VEGFR2, while the insensitive cell lines express VEGFR1. Compounds **18** and **20** are promising candidates for further antimycobacterial activity optimization, while compound **29** has good potential to be developed as a selective antineoplastic agent.

## Figures and Tables

**Table 1:** Prepared compounds ordered by increasing calculated lipophilicity (log *P*) with their antimycobacterial activity expressed as minimum inhibitory concentration (MIC) and HepG2 cytotoxicity.

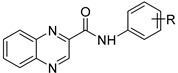							
**Cmpd**	**R**	**Log*P***	**MIC µg/mL (µM)**			**HepG2**	**SI ^††^**
			***Mtb* H37Ra**	***M. smeg***	***M. aurum***	**IC_50_ (µM)**	
**1**	H	2.43	≥500	≥250	≥250	>50 ^†^	
**2**	3-OH	2.04	15.625 (58.9)	≥125	≥125	>250 ^†^	>4.20
**3**	4-OH	2.04	62.5 (235.6)	≥500	≥500	>250 ^†^	>1.01
**4**	3,5-diOCH_3_	2.18	62.5 (202)	≥125	≥125	>25 ^†^	>0.12
**5**	4-OCH_3_	2.31	≥125	≥125	≥125	>50 ^†^	
**6**	Cyclohexyl *	2.32	31.25 (122.4)	31.25	≥500	>1000	>8.17
**7**	2-F	2.59	≥500	62.5	≥500	>100 ^†^	
**8**	3-F	2.59	250	≥500	≥500	>100 ^†^	
**9**	4-F	2.59	15.625 (58.5)	≥500	≥500	>100 ^†^	>1.71
**10**	4-N(CH_3_)_2_	2.72	≥500	≥500	≥500	>250 ^†^	
**11**	2,4-diF	2.75	≥500	≥500	≥500	>250 ^†^	
**12**	2-Cl	2.99	≥125	≥125	≥125	>50 ^†^	
**13**	3-Cl	2.99	≥500	≥500	≥500	>25 ^†^	
**14**	3-CF_3_	3.35	≥500	≥125	≥125	>25 ^†^	
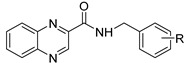							
**Cmpd**	**R**	**Log*P***	**MIC µg/mL (µM)**			**HepG2**	**SI ^††^**
			***Mtb*** **H37Ra**	***M. smeg***	***M. aurum***	**IC_50_ (µM)**	
**15**	H	2.50	15.625 (59.3)	≥125	≥125	>1000	>17
**16**	3-OH	2.11	31.25 (111.9)	62.5	≥ 500	600.7	5.40
**17**	4-OH	2.11	62.5 (223.8)	≥500	500	>250 ^†^	>1.1
**18**	2,5-diOCH_3_	2.25	3.91 (12.1)	≥500	≥500	>1000	82.60
**19**	3-OCH_3_	2.38	15.625 (47)	≥500	≥500	>1000	>21.3
**20**	4-OCH_3_	2.38	3.91 (11.8)	≥250	125	209.4	16.1
**21**	3-F	2.66	7.81 (27.8)	≥500	≥500	248.0	8.92
**22**	2,4-diF	2.75	≥500	≥500	≥500	>25 ^†^	
**23**	2-CH_3_	2.99	7.81 (28.2)	≥125	≥125	156.7	5.60
**24**	4-CH_3_	2.99	7.81 (28.2)	≥250	≥250	87.7	3.11
**25**	2-Cl	3.06	15.625 (52.5)	≥500	≥500	527.5	22.45
**26**	3-Cl	3.06	7.81 (26.2)	31.25	31.25	140.1	5.34
**27**	4-Br	3.33	7.81 (22.8)	≥500	≥500	>100 ^†^	>4.4
**28**	3-CF_3_	3.42	3.91 (11.8)	250	≥500	48.6	4.12
**29**	Naphthyl **	3.50	≥250	≥125	≥125	37.3	
**30**	2,4-diCl	3.62	≥500	≥125	≥125	>100 ^†^	
**31**	3,4-diCl	3.62	7.81 (23.5)	≥500	≥500	66.9	2.80
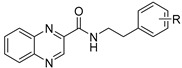							
**Cmpd**	**R**	**Log*P***	**MIC µg/mL (µM)**			**HepG2**	**SI ^††^**
			***Mtb*** **H37Ra**	***M. smeg***	***M. aurum***	**IC_50_ (µM)**	
**32**	H	2.78	≥500	≥500	≥500	>1000	
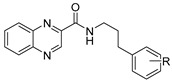							
**Cmpd**	**R**	**Log*P***	**MIC µg/mL (µM)**			**HepG2**	**SI ^††^**
			***Mtb*** **H37Ra**	***M. smeg***	***M. aurum***	**IC_50_ (µM)**	
**33**	H	3.20	15.625 (53.6)	≥500	≥500	145.9	2.7
**Standards**		**Log*P***	**MIC µg/mL (µM)**			**HepG2**	**SI ^††^**
			***Mtb*** **H37Ra**	***M. smeg***	***M. aurum***	**IC_50_ (µM)**	
PZA		−1.31	≥500 (≥4061) ^†††^	≥500	≥500	>1000	
INH		−0.64	0.25 (1.82)	15.625	3.91	>1000	>459.5
CPX		1.32	0.25 (0.75)	0.125	0.0156	>500 ^†^	>666.7
RIF		4.24	0.003125 (0.0038)	12.5	0.39	>500 ^†^	>131.579
Tamoxifen		6.07	na	na	na	19.56	na
* 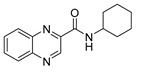				** 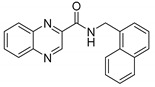			

^†^ Measurements at higher concentrations were not feasible due to precipitation in the testing medium. ^††^ Selectivity index (SI) = IC_50_ (µM)/MIC *Mtb* H37Ra (µM). ^†††^ PZA is inactive at pH 6.6 (pH of testing medium), activity is observed in acidic medium (MIC at pH of 6 is <3.91 µg/mL) [16]. * Structure of compound **6**. ** Structure of compound **29**.

**Table 2:** A comparison between previously published compounds with their corresponding title compounds (bearing the same substituent) in terms of activity against *Mycobacterium tuberculosis* H37Ra.

	Semelkova et al. [10]	This Paper	
R	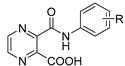	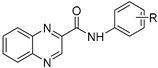	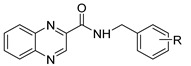
H	≥500	≥500 (**1**)	15.625 (**15**)
4-OCH_3_	≥500	≥125 (**5**)	3.91 (**20**)
3-CF_3_	250	≥500 (**14**)	3.91 (**28**)

**Table 3:** Biological activity profile for compound **29**.

**Activity against Mycobacterial Strains Expressed as MIC (µM)**							
*Mtb* H37Ra			*M. smeg*			*M. aurum*	
≥798			≥399			≥399	
**Activity against Bacterial Strains Expressed as MIC (µM)**							
*S. aureus*	MRSA	*E. faecalis*	*E. coli*	*P. aeruginosa*	*S. epidermidis*	*K. pneumoniae*	*S. marcescens*
≥500	≥500	≥500	≥500	≥500	≥500	≥500	≥500
**Activity against Fungal Strains Expressed as MIC (µM)**							
*C. albicans*	*C. krusei*	*C. parapsilosis*	*C. tropicalis*	*A. flavus*	*L. corymbifera*	*T. interdigitale*	*A. fumigatus*
≥500	≥500	≥500	≥500	≥500	≥500	≥500	≥500
**Cytotoxicity against Human Cell Lines Expressed as IC_50_ (µM)**							
HepG2		HK-2	U-87 MG		A498	PC-3	SK-OV-3
37.3		>250	>250		>250	68.5	49.6

For standards, refer to Supplementary Material S2.

## Data Availability

The authors confirm that the data supporting this study are available within the article and its Supplementary Materials. Additional data available upon request.

## Supplementary Materials

The following are available online at https://www.mdpi.com/article/10.3390/ph14080768/s1, Supplementary Material S1: Analytical data and copies of ^1^H-NMR and 13C-NMR spectra of title compounds; Supplementary Material S2: Detailed experimental procedures; Figure S1: HepG2 IC50 curves of selected compounds; Supplementary Material S3: In silico modeling–Detailed experimental procedures; Table S1: Results of molecular docking of compound **29** to the selected human targets (ordered by docking score), Table S2: Top 10 non-redundant human kinases as potential targets for compound **29**, Table S3: Top 10 scoring structures of human VEGFR2 after docking of compound **29**.
