# Supplementary material for: Synthesis, Biological Evaluation, and In Silico Modeling of N-Substituted Quinoxaline-2-Carboxamides
Source: Pharmaceuticals (Basel). 2021 Aug 4;14(8):768. doi: 10.3390/ph14080768 (PMC8399443; doi:10.3390/ph14080768)
Supplement: Supplementary file 1 [file pharmaceuticals-14-00768-s001.zip › Supplementary Material 1 - Analytical data.pdf]

Synthesis, Biological Evaluation and In Silico Modelling of *N*-substituted Quinoxaline-2-carboxamides

Ghada Bouz, Sarah Bouz, Ondřej Janďourek, Klára Konečná, Pavel Bárta, Jarmila Vinšová, Martin Doležal, and Jan Zitko

Faculty of Pharmacy in Hradec Králové, Charles University, Akademika Heyrovského 1203, 50005 Hradec Králové, Czech Republic

Supplementary Material 1–*Analytical data of title compounds*

For each compound present in the main text (compounds **1**–**33**), we report in this supplementary the physical description, melting points, yields, ^{1}H-NMR, ^{13}C-NMR, IR, and elemental analysis. Copies of ^{1}H-NMR and ^{13}C-NMR spectra of title compounds are also attached. The chemical structure of each compound is shown on the ^{1}H-NMR spectra. Solvent signals omitted in ^{1}H-NMR.

Compound **1**

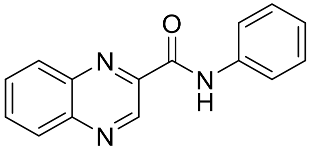

***N*-phenylquinoxaline-2-carboxamide**. M.p.: 182.2–183.5˚C {in literature M.p.: 170–171 ˚C [1]}. Yield: 50%. White solid powder. ^{1}H-NMR (500 MHz, DMSO-d_6) δ 10.81 (s, 1H, amide), 9.54 (s, 1H, pyrazine), 8.34–8.27 (m, 1H, aromatic), 8.25–8.18 (m, 1H, aromatic), 8.05–7.97 (m, 2H, aromatic), 7.97–7.91 (m, 2H, aromatic), 7.45–7.37 (m, 2H, aromatic), 7.20–7.11 (m, 1H, aromatic). ^{13}C-NMR (126 MHz, DMSO-d_6) δ 162.17, 144.90, 144.13, 143.12, 139.83, 138.34, 132.27, 131.55, 129.74, 129.30, 128.94, 124.50, 120.75, 120.66. IR (ATR-Ge, cm^{-1}): 3361 (NH, CONH), 1676 (CO, CONH), 1596, 1571, 1532 (aromatic). Calculated for $C_{15}H_{11}N_3O$ (249.27 g/mol): C, 72.28%; H, 4.45%; N, 16.86%. Found: C, 71.85%; H, 4.33%; N, 16.66%. CAS# 37648-63-8.

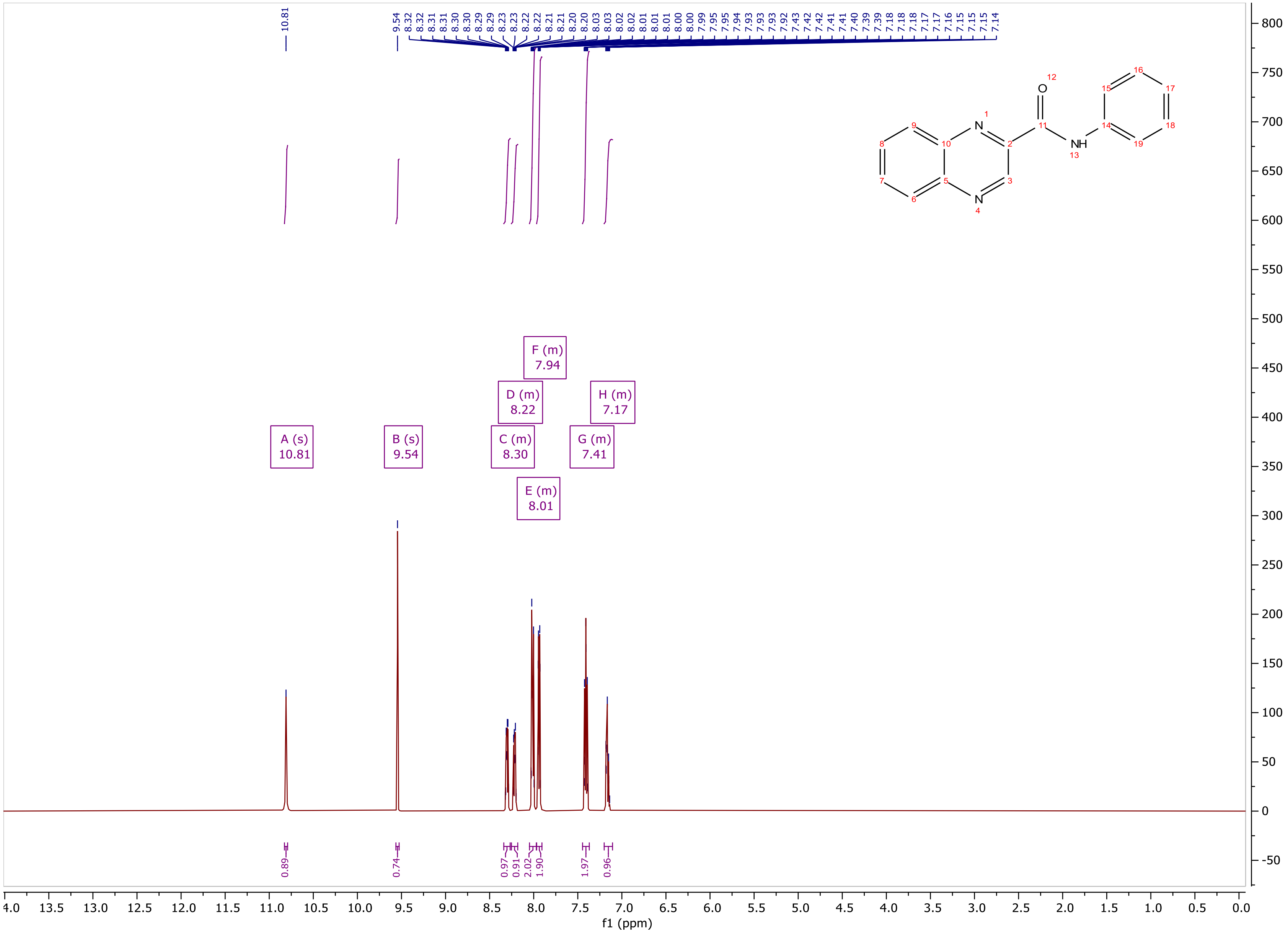

10.81
9.54
8.32
8.32
8.31
8.30
8.30
8.29
8.29
8.23
8.23
8.22
8.22
8.21
8.21
8.20
8.03
8.03
8.02
8.02
8.01
8.01
8.00
7.99
7.95
7.95
7.94
7.93
7.93
7.92
7.43
7.42
7.41
7.41
7.40
7.39
7.39
7.18
7.18
7.17
7.17
7.16
7.15
7.15
7.14
A (s) 10.81
B (s) 9.54
C (m) 8.30
D (m) 8.22
E (m) 8.01
F (m) 7.94
G (m) 7.41
H (m) 7.17
0.89
0.74
0.97
0.91
2.02
1.90
1.97
0.96
f1 (ppm)

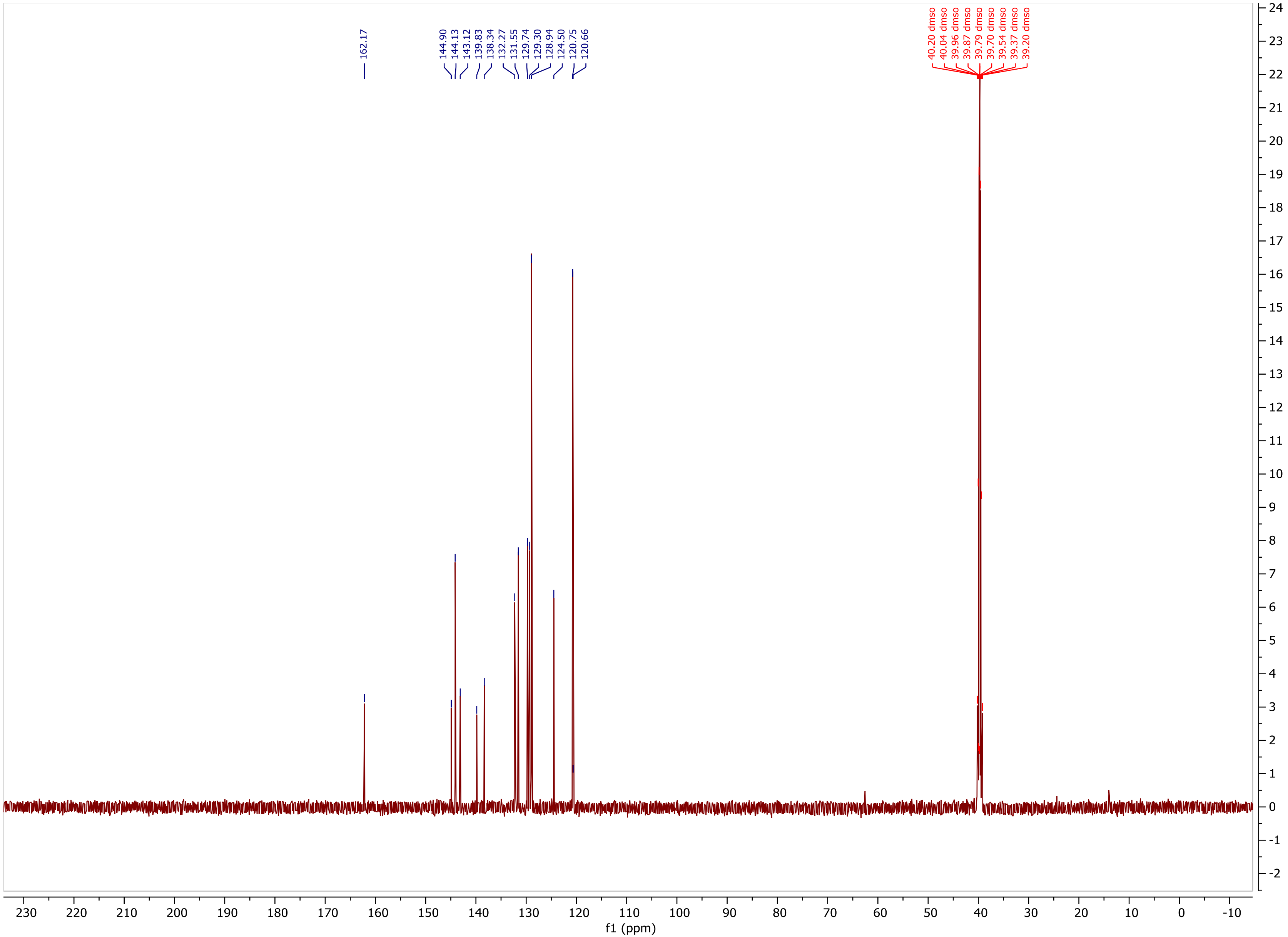

162.17
144.90
144.13
143.12
139.83
138.34
132.27
131.55
129.74
129.30
128.94
124.50
120.75
120.66
40.20 dmso
40.04 dmso
39.96 dmso
39.87 dmso
39.79 dmso
39.70 dmso
39.54 dmso
39.37 dmso
39.20 dmso
f1 (ppm)

Compound 2

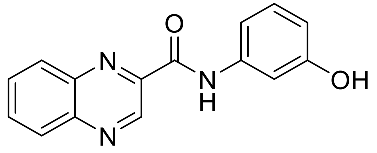

$\textbf{\textit{N}}$-(3-hydroxyphenyl)quinoxaline-2-carboxamide. M.p.: 238.1–241.2˚C. Yield: 80%. Yellow-orange solid powder. ^{1}H-NMR (500 MHz, DMSO-d_6) δ 10.65 (s, 1H, amide), 9.53 (s, 1H, pyrazine), 9.52 (s, 1H, hydroxy), 8.33–8.26 (m, 1H, aromatic), 8.26–8.16 (m, 1H, aromatic), 8.04–7.95 (m, 2H, aromatic), 7.54–7.49 (m, 1H, aromatic), 7.34–7.28 (m, 1H, aromatic), 7.21–7.13 (m, 1H, aromatic), 6.61–6.53 (m, 1H, aromatic). ^{13}C-NMR (126 MHz, DMSO-d_6) δ 162.32, 158.10, 145.26, 144.39, 143.37, 140.09, 139.60, 132.52, 131.80, 130.04, 129.89, 129.55, 111.94, 111.75, 108.04. IR (ATR-Ge, cm^{-1}): 3376 (NH, CONH), 1726 (CO, CONH), 1684, 1598, 1563 (aromatic). Calculated for $C_{15}H_{11}N_3O_2$ (265.27 g/mol): C, 67.92%; H, 4.18%; N, 15.84%. Found: C, 67.81%; H, 4.16%; N, 15.54%. CAS# 1206970-59-3.

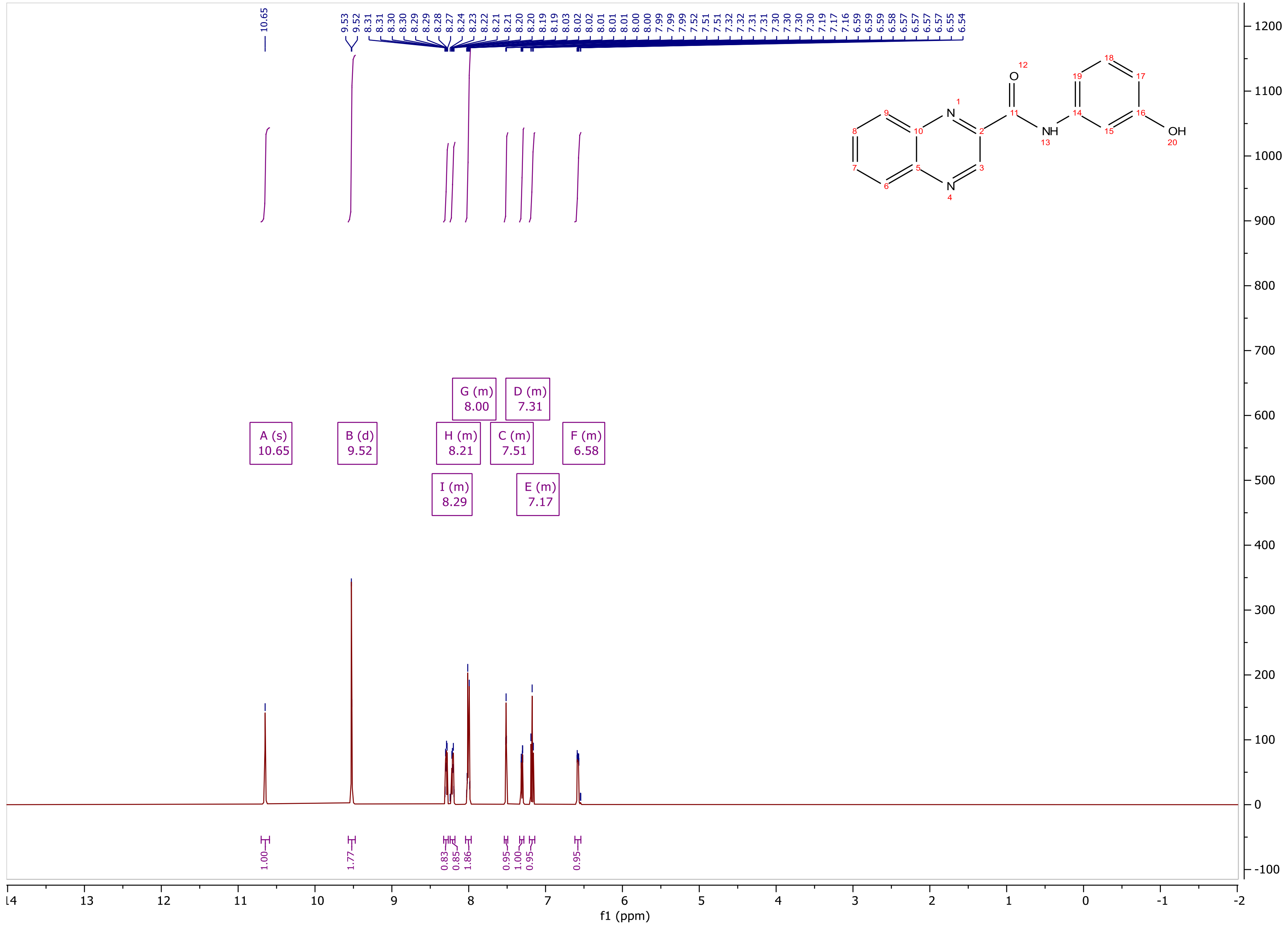

A (s) 10.65
B (d) 9.52
I (m) 8.29
H (m) 8.21
G (m) 8.00
C (m) 7.51
D (m) 7.31
E (m) 7.17
F (m) 6.58
f1 (ppm)
OH
NH
O
N

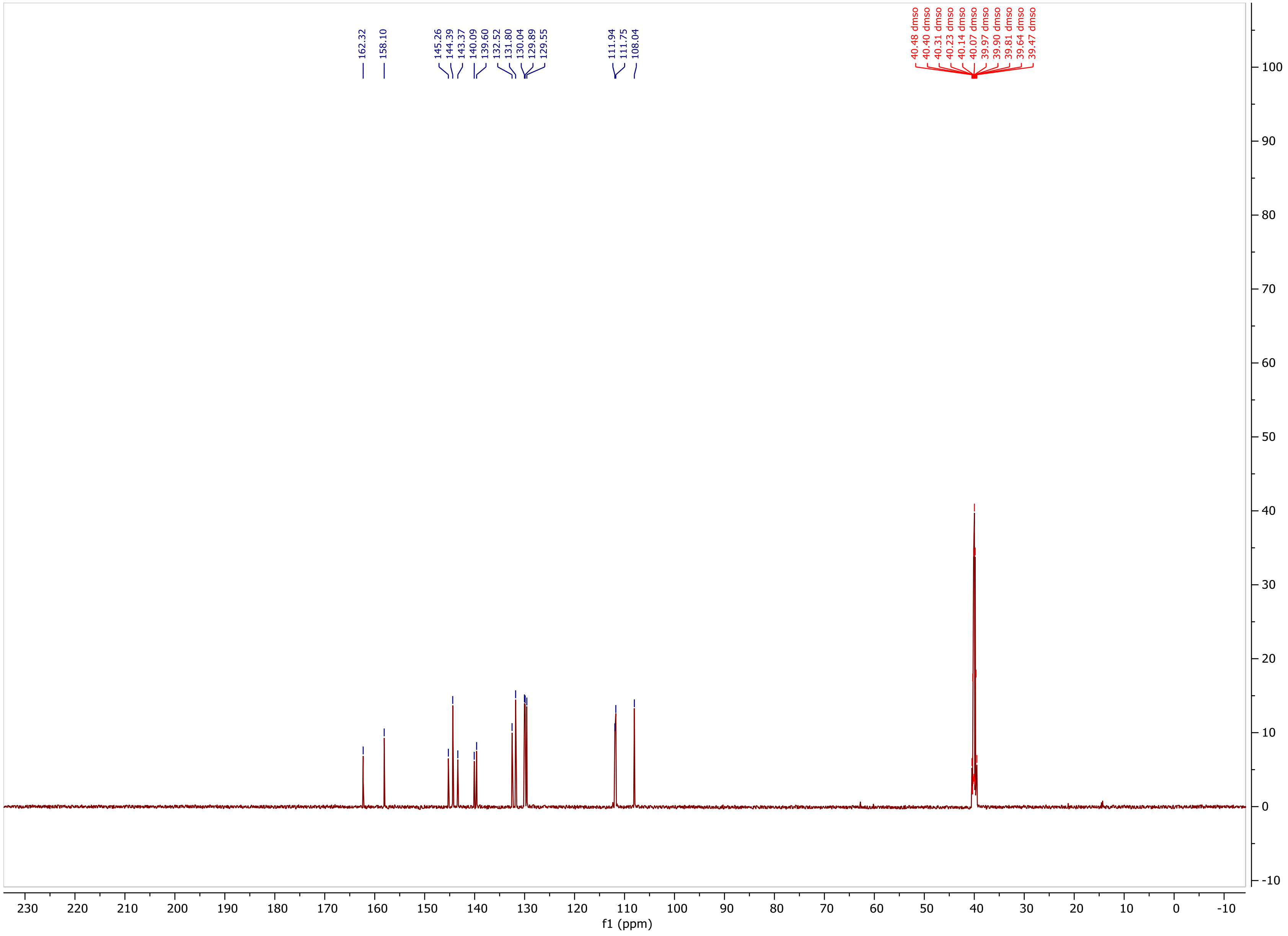

162.32
158.10
145.26
144.39
143.37
140.09
139.60
132.52
131.80
130.04
129.89
129.55
111.94
111.75
108.04
40.48 dmso
40.40 dmso
40.31 dmso
40.23 dmso
40.14 dmso
40.07 dmso
39.97 dmso
39.90 dmso
39.81 dmso
39.64 dmso
39.47 dmso
f1 (ppm)

Compound 3

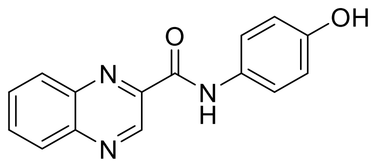

***N*-(4-hydroxyphenyl)quinoxaline-2-carboxamide**. M.p.: 224.7–226.1˚C. Yield: 68%. Orange-red solid powder. ^{1}H-NMR (500 MHz, DMSO-d_6) δ 10.60 (s, 1H, amide), 9.52 (s, 1H, pyrazine), 9.36 (s, 1H, hydroxy), 8.31–8.17 (m, 2H, aromatic), 8.03–7.96 (m, 2H, aromatic), 7.74–7.67 (m, 2H, aromatic), 6.83–6.76 (m, 2H, aromatic).^{13}C-NMR (126 MHz, DMSO-d_6) δ 161.52, 154.38, 145.13, 144.09, 143.04, 139.86, 132.10, 131.49, 129.93, 129.69, 129.28, 122.48, 115.31. IR (ATR-Ge, cm^{-1}): 3366 (NH, CONH), 1674 (CO, CONH), 1614, 1538, 1516 (aromatic). Calculated for $C_{15}H_{11}N_3O_2$ (265.27 g/mol): C, 67.92%; H, 4.18%; N, 15.84%. Found: C, 67.67%; H, 4.01%; N, 15.54%. CAS# 1912839-05-4.

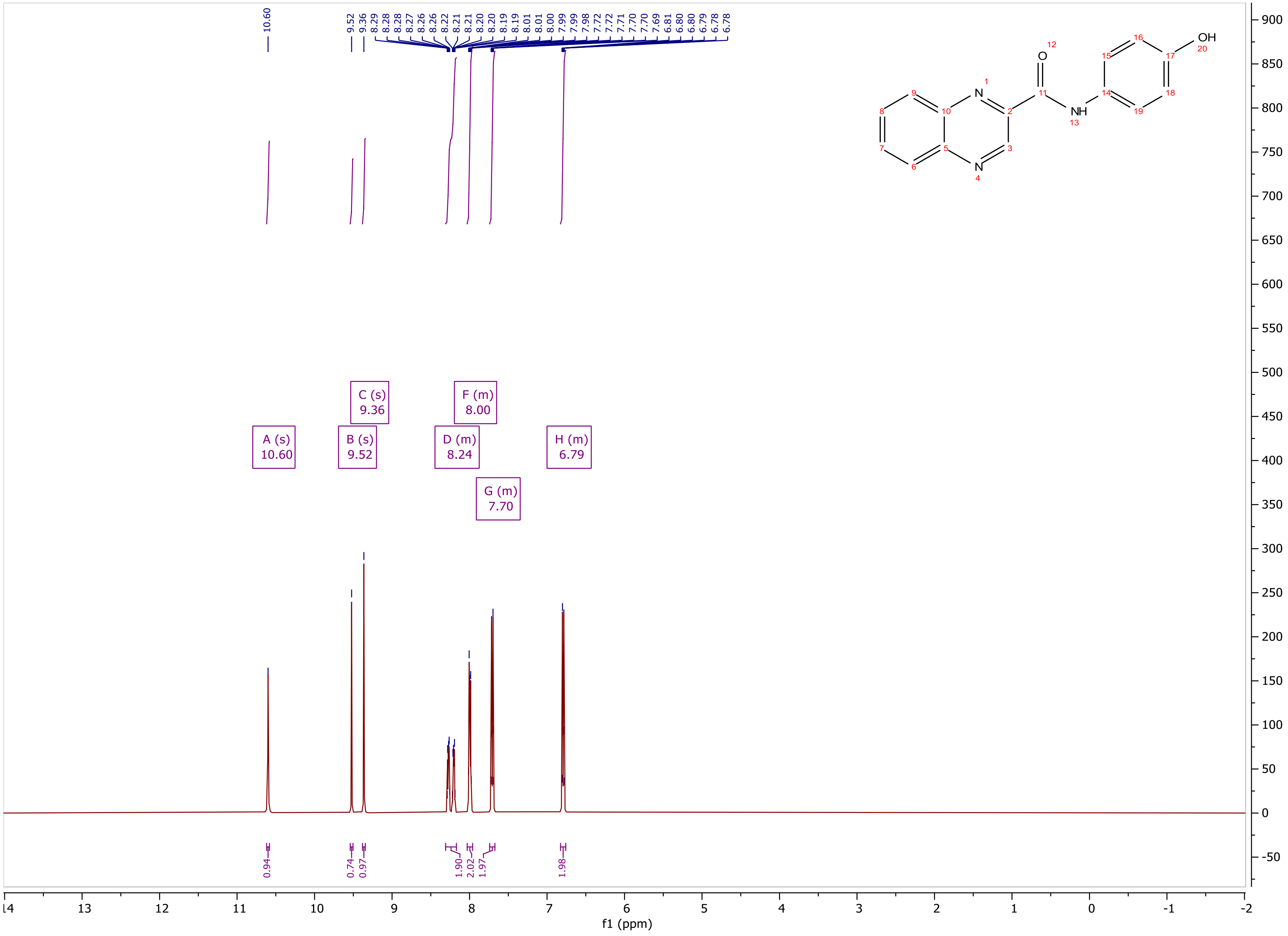

10.60
9.52
9.36
8.29
8.28
8.28
8.27
8.26
8.26
8.22
8.21
8.21
8.20
8.20
8.19
8.19
8.01
8.01
8.00
7.99
7.99
7.98
7.72
7.72
7.71
7.70
7.70
7.69
6.81
6.80
6.80
6.79
6.78
6.78
A (s) 10.60
B (s) 9.52
C (s) 9.36
D (m) 8.24
F (m) 8.00
G (m) 7.70
H (m) 6.79
0.94
0.74
0.97
1.90
2.02
1.97
1.98
f1 (ppm)
O
N
NH
N
OH

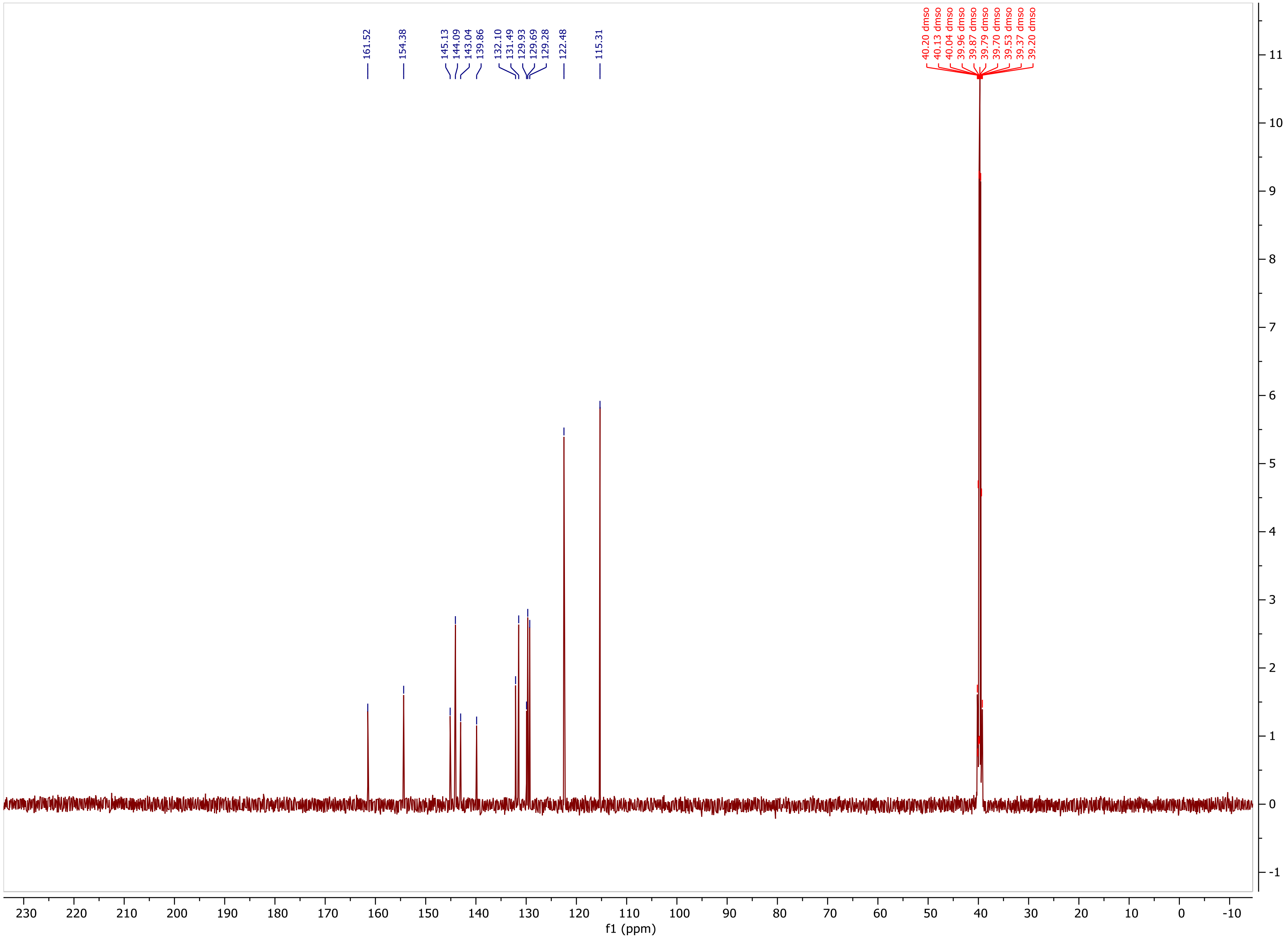
161.52
154.38
145.13
144.09
143.04
139.86
132.10
131.49
129.93
129.69
129.28
122.48
115.31
40.20 dmso
40.13 dmso
40.04 dmso
39.96 dmso
39.87 dmso
39.79 dmso
39.70 dmso
39.53 dmso
39.37 dmso
39.20 dmso
f1 (ppm)

Compound 4

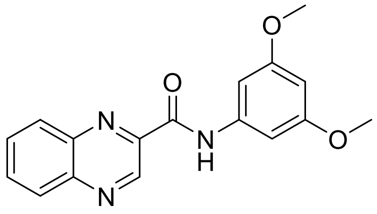

***N*-(3,5-dimethoxyphenyl)quinoxaline-2-carboxamide**. M.p.: 184.2–186.1˚C. Yield: 70%. Yellow fluffy solid. ^{1}H-NMR (500 MHz, DMSO-d_6) δ 10.72 (s, 1H, amide), 9.53 (s, 1H, pyrazine), 8.33–8.26 (m, 1H, aromatic), 8.24–8.18 (m, 1H, aromatic), 8.05–7.97 (m, 2H, aromatic), 7.26 (d, J = 2.2 Hz, 2H, aromatic), 6.31 (t, J = 2.3 Hz, 1H, aromatic), 3.76 (s, 6H, methoxy). ^{13}C-NMR (126 MHz, DMSO-d_6) δ 162.14, 160.65, 144.71, 144.09, 143.13, 140.00, 139.77, 132.30, 131.56, 129.71, 129.30, 98.89, 96.59, 55.38. IR (ATR-Ge, cm^{-1}): 3339 (NH, CONH), 1678 (CO, CONH), 1617, 1599, 1573 (aromatic). Calculated for $C_{17}H_{15}N_3O_3$ (309.33 g/mol): C, 66.01%; H, 4.89%; N, 13.58%. Found: C, 65.92%; H, 4.88%; N, 13.52%. CAS# 689265-59-6.

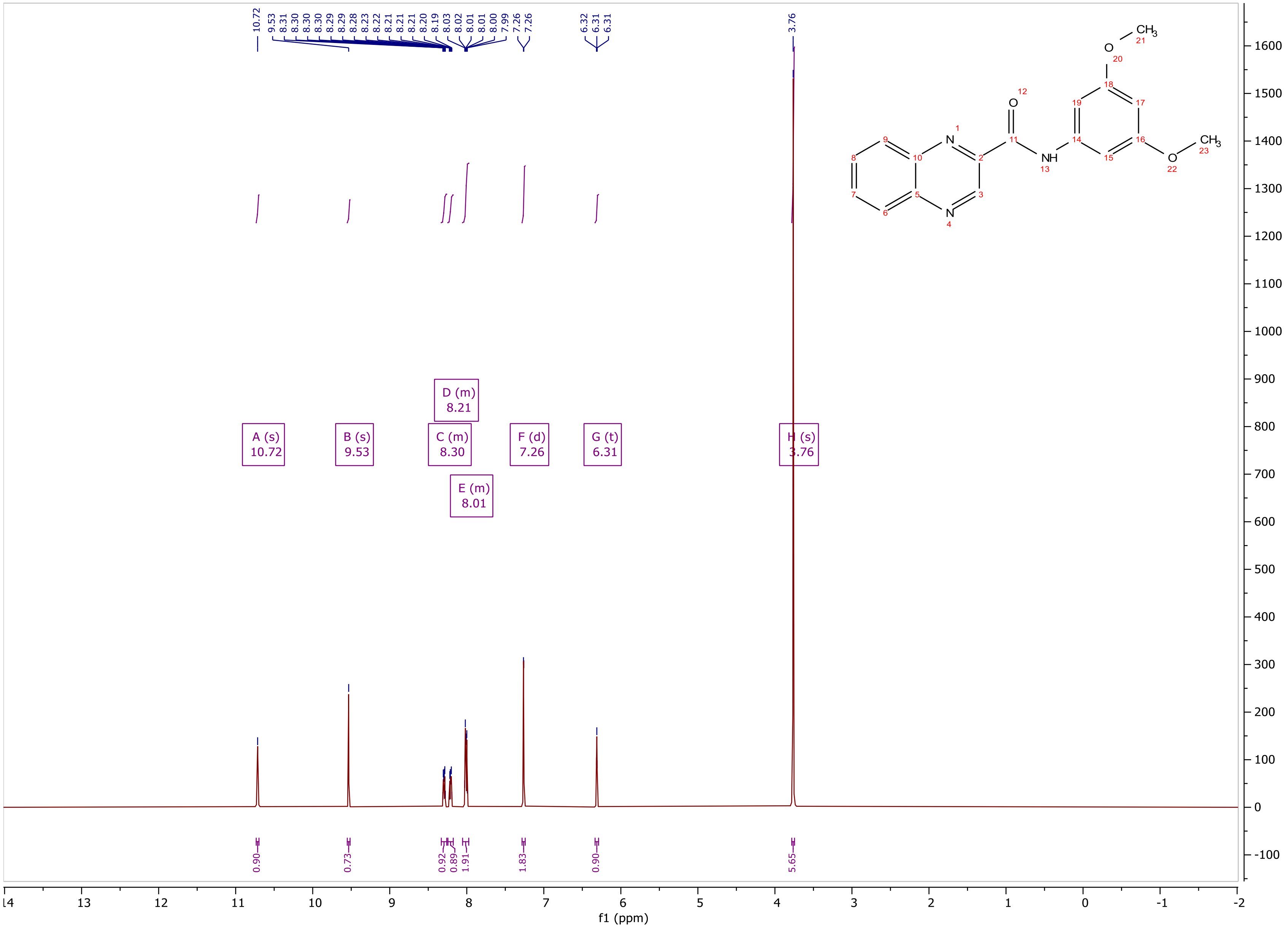

10.72
9.53
8.31
8.30
8.30
8.30
8.29
8.29
8.28
8.23
8.22
8.21
8.21
8.21
8.20
8.19
8.03
8.02
8.01
8.01
8.00
7.99
7.26
7.26
6.32
6.31
6.31
3.76
A (s)
10.72
B (s)
9.53
C (m)
8.30
D (m)
8.21
E (m)
8.01
F (d)
7.26
G (t)
6.31
H (s)
3.76
0.90
0.73
0.92
0.89
1.91
1.83
0.90
5.65
f1 (ppm)
N
N
O
NH
O
CH_3
O
CH_3

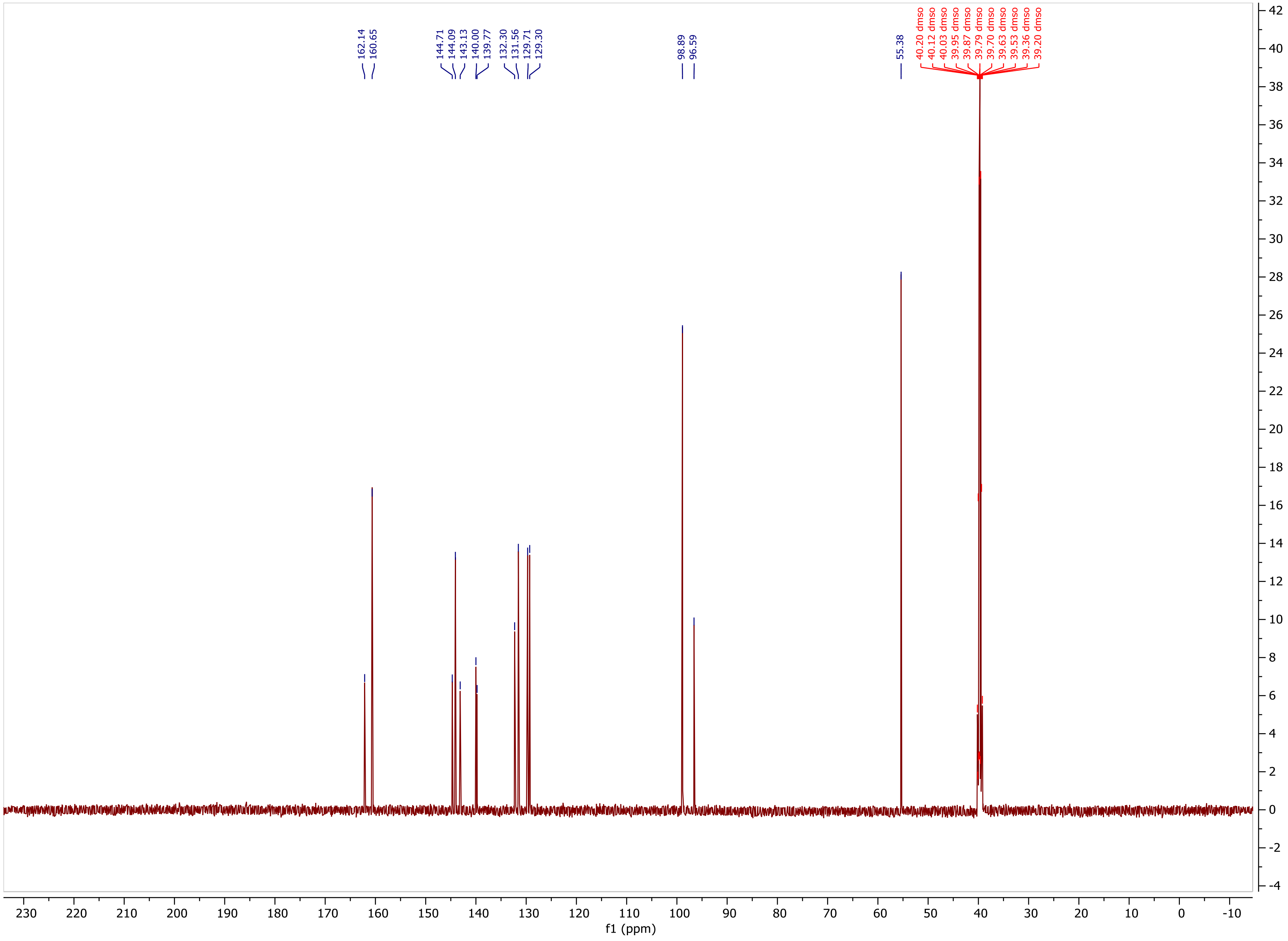

162.14
160.65
144.71
144.09
143.13
140.00
139.77
132.30
131.56
129.71
129.30
98.89
96.59
55.38
40.20 dmso
40.12 dmso
40.03 dmso
39.95 dmso
39.87 dmso
39.79 dmso
39.70 dmso
39.63 dmso
39.53 dmso
39.36 dmso
39.20 dmso
f1 (ppm)

Compound 5

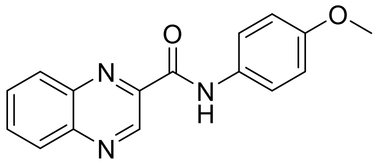

***N*-(4-methoxyphenyl)quinoxaline-2-carboxamide**. M.p.: 183.1–184.9˚C {in literature M.p.: 176–178°C [1]}. Yield: 77%. White powder. ^{1}H-NMR (600 MHz, Chloroform-*d*) δ 9.78 (s, 1H, amide), 9.76 (s, 1H, pyrazine), 8.26–8.15 (m, 2H, aromatic), 7.94–7.83 (m, 2H, aromatic), 7.79–7.73 (m, 2H, aromatic), 7.00–6.94 (m, 2H, aromatic), 3.84 (s, 3H, methoxy). ^{13}C-NMR (151 MHz, Chloroform-*d*) δ 160.64, 156.73, 143.96, 143.86, 143.47, 140.01, 131.69, 130.97, 130.51, 129.58, 129.53, 121.44, 114.32, 55.48. IR (ATR-Ge, cm^{-1}): 3369 (NH, CONH), 1683 (CO, CONH), 1559, 1531, 1512 (aromatic). Calculated for $C_{16}H_{13}N_3O_2$ (279.30 g/mol): C, 68.81; H, 4.69%; N, 15.05%. Found: C, 68.77%; H, 4.60%; N, 15.24%. CAS#901599-43-7.

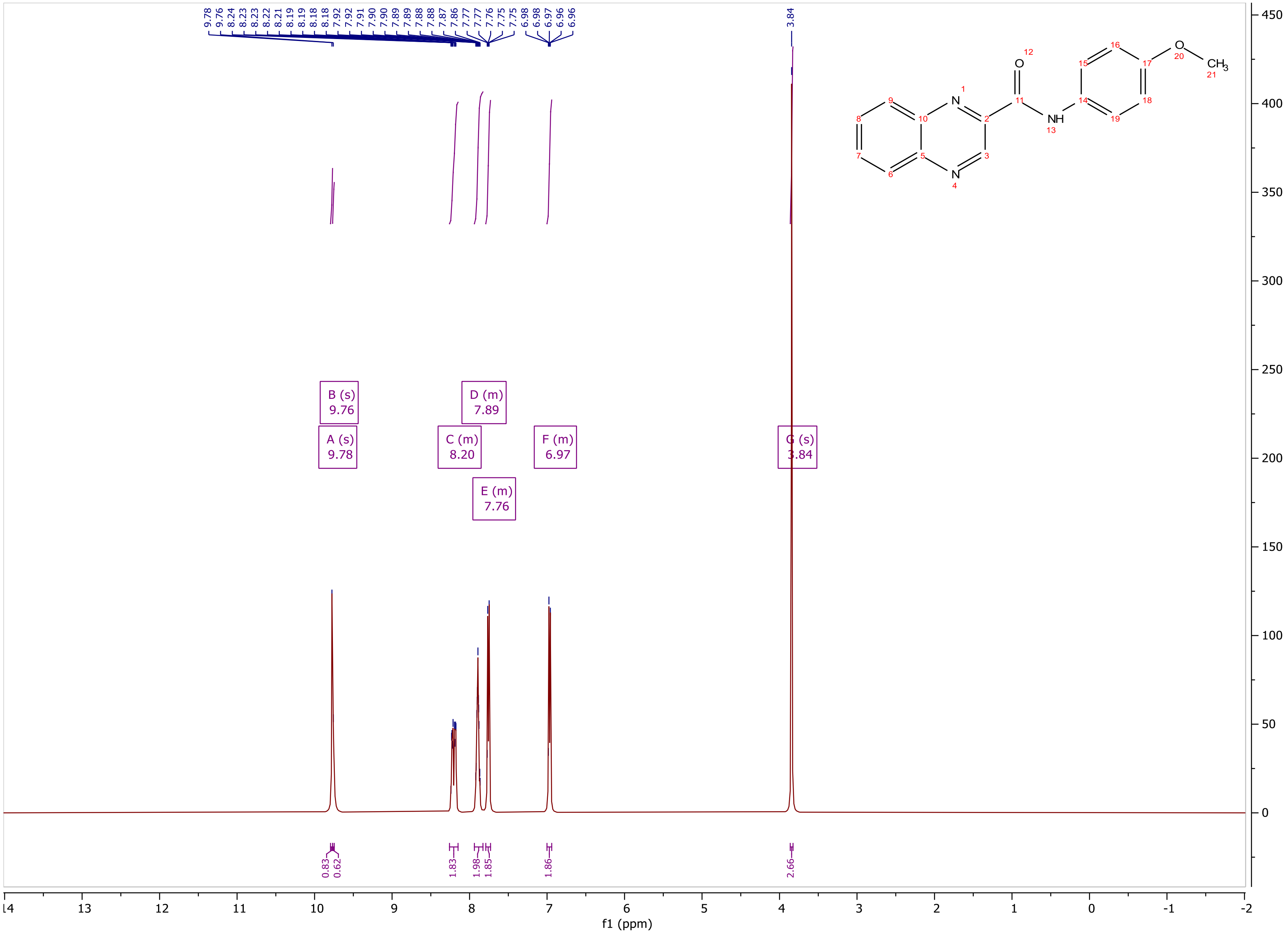

9.78
9.76
8.24
8.23
8.22
8.21
8.19
8.19
8.18
8.18
7.92
7.92
7.91
7.90
7.90
7.89
7.89
7.88
7.88
7.87
7.86
7.77
7.77
7.76
7.75
7.75
6.98
6.98
6.97
6.96
6.96
3.84
B (s) 9.76
A (s) 9.78
C (m) 8.20
D (m) 7.89
E (m) 7.76
F (m) 6.97
G (s) 3.84
0.83
0.62
1.83
1.98
1.85
1.86
2.66
f1 (ppm)
O
N
NH
N
CH_3

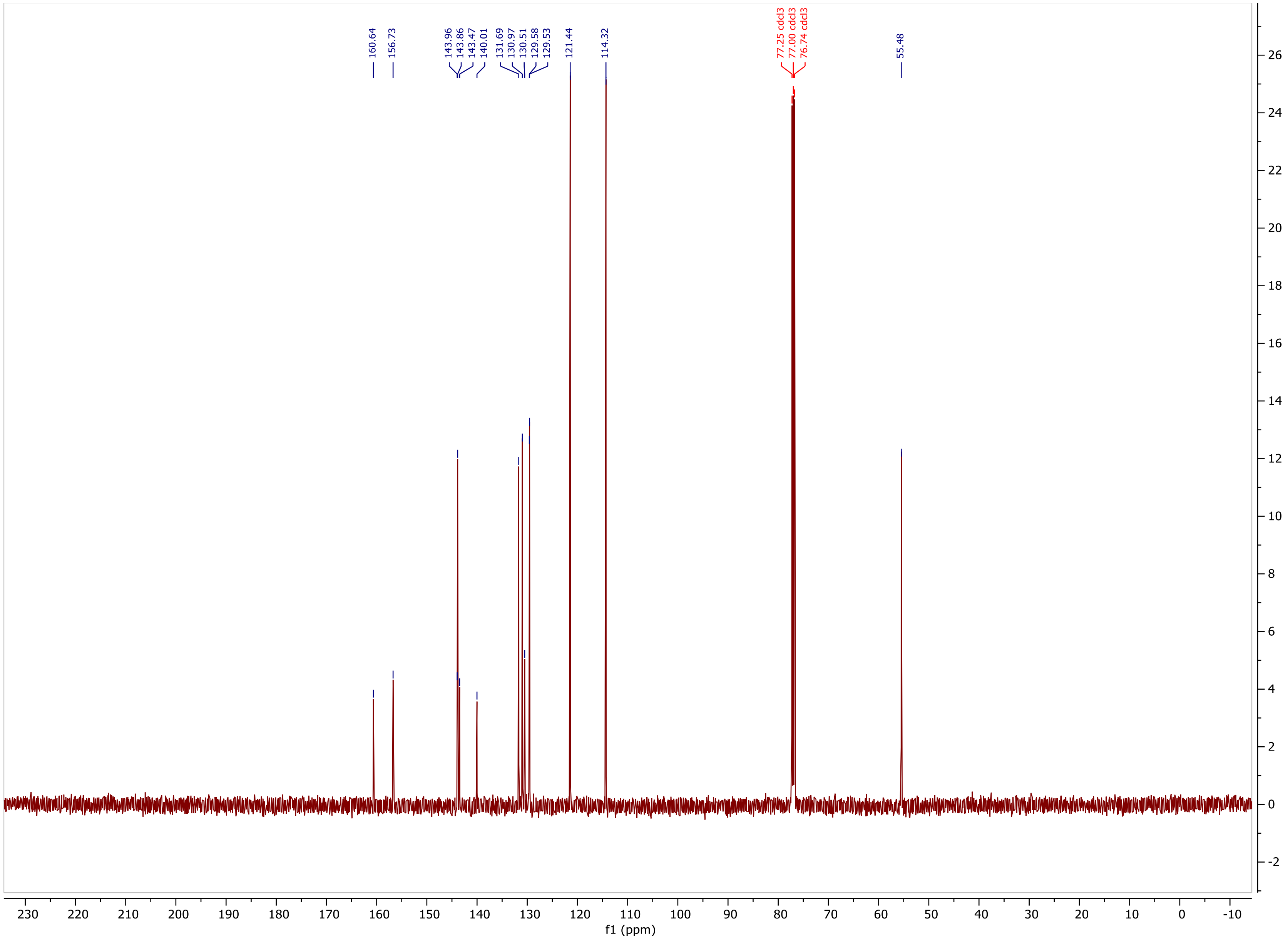

160.64
156.73
143.96
143.86
143.47
140.01
131.69
130.97
130.51
129.58
129.53
121.44
114.32
77.25 cdcl3
77.00 cdcl3
76.74 cdcl3
55.48
f1 (ppm)

Compound 6

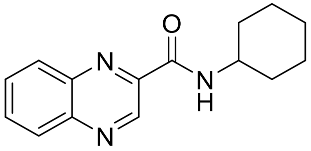

***N*-cyclohexylquinoxaline-2-carboxamide**. M.p.: 96–97.4˚C {in literature M.p.: 98–100˚C [1]}. Yield: 60%. Light beige powder. ^{1}H-NMR (600 MHz, DMSO-d_6) δ 9.45 (s, 1H, amide), 8.71 (s, 1H, pyrazine), 8.25–8.15 (m, 2H, aromatic), 7.99–7.96 (m, 2H, aromatic), 1.88–1.81 (m, 3H, cyclohexyl), 1.79–1.71 (m, 3H, cyclohexyl), 1.51–1.44 (m, 2H, cyclohexyl), 1.39–1.30 (m, 2H, cyclohexyl). ^{13}C-NMR (151 MHz, DMSO-d_6) δ 162.32, 144.82, 143.94, 143.03, 139.91, 131.91, 131.36, 129.59, 129.23, 48.47, 32.29, 25.28, 25.04. IR (ATR-Ge, cm^{-1}): 3270 (NH, CONH), 1669 (CO, CONH), 1540, 1531, 1517 (aromatic). Calculated for $C_{15}H_{17}N_3O$ (255,32 g/mol): C, 70.56; H, 6.71%; N, 16.46%. Found: C, 70.23%; H, 6.64%; N, 16.22%. CAS#155129-19-4.

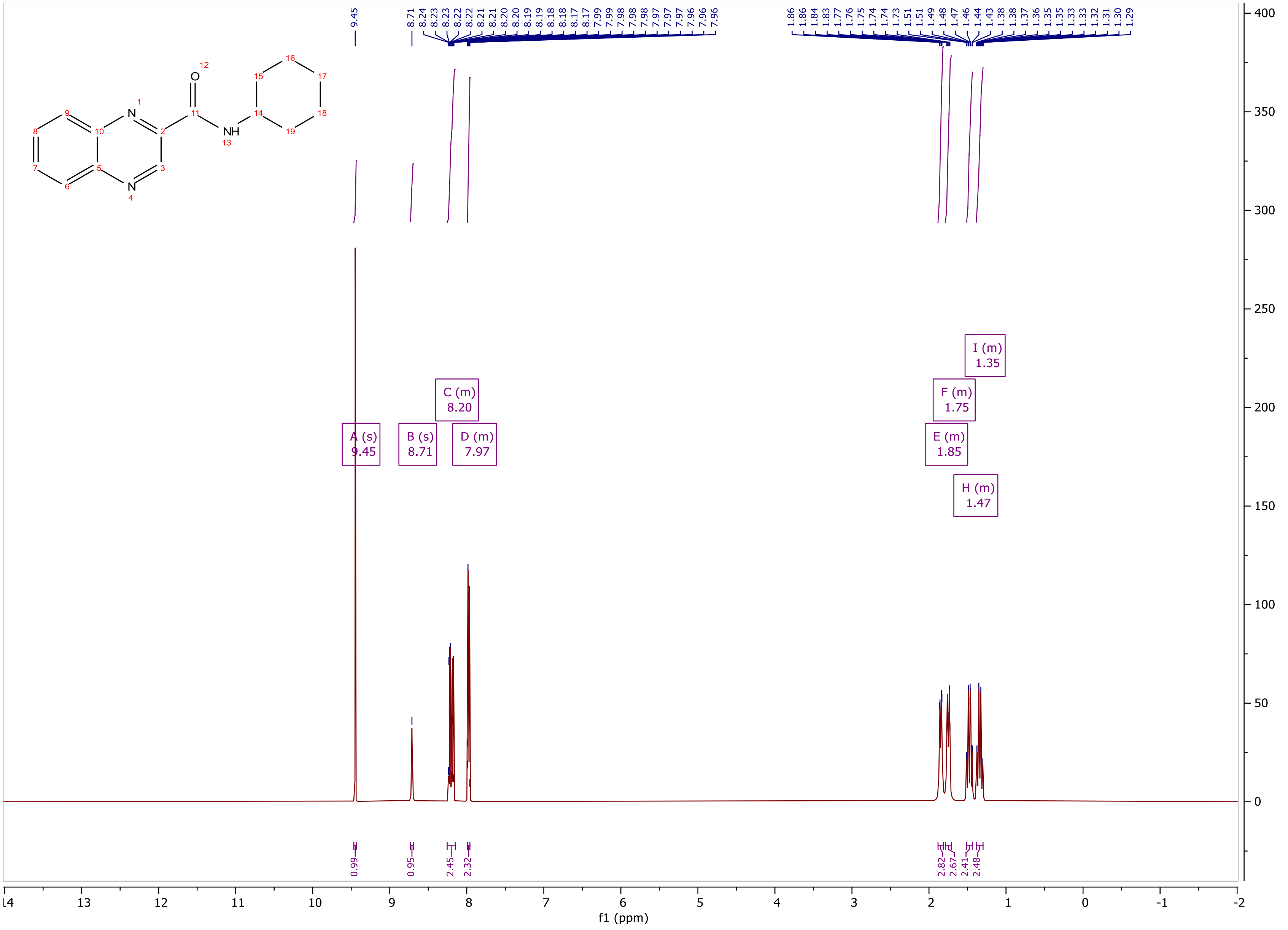

A (s)
9.45
B (s)
8.71
C (m)
8.20
D (m)
7.97
E (m)
1.85
F (m)
1.75
H (m)
1.47
I (m)
1.35
0.99
0.95
2.45
2.32
2.82
2.67
2.41
2.48
f1 (ppm)
NH
N
O

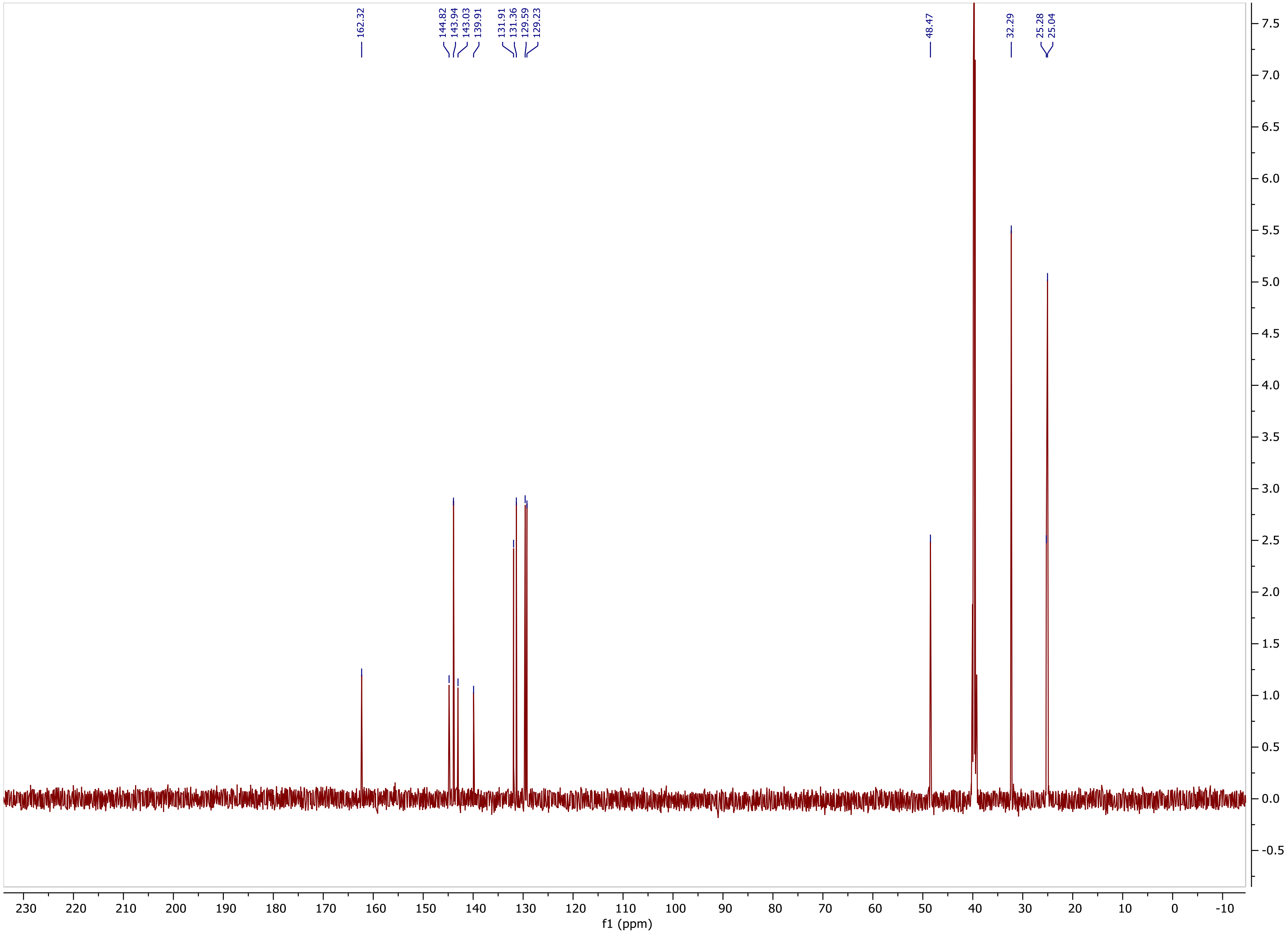

162.32
144.82
143.94
143.03
139.91
131.91
131.36
129.59
129.23
48.47
32.29
25.28
25.04
f1 (ppm)

Compound 7

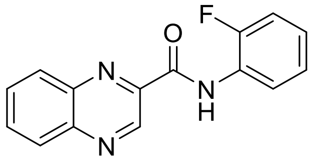

***N*-(2-fluorophenyl)quinoxaline-2-carboxamide**. M.p.: 180.4–182.0˚C. Yield: 55%. Light green powder. ^{1}H-NMR (600 MHz, Chloroform-*d*) δ 9.90 (s, 1H, amide), 9.77 (s, 1H, pyrazine), 8.27–8.15 (m, 2H, aromatic), 7.97–7.86 (m, 2H, aromatic), 7.83–7.75 (m, 1H, aromatic), 7.52–7.46 (m, 1H, aromatic), 7.43–7.32 (m, 1H, aromatic), 6.94–6.86 (m, 1H, aromatic).^{13}C-NMR (151 MHz, Chloroform-*d*) δ 164.07, 162.12, 161.02, 143.77, 142.90, 139.93, 131.99, 131.15, 129.63, 129.54, 115.17, 111.63, 111.46, 107.43, 107.21. IR (ATR-Ge, cm^{-1}): 3361 (NH, CONH), 1668 (CO, CONH), 1629, 1589, 1571 (aromatic). Calculated for $C_{15}H_{10}FN_3O$ (267.26 g/mol): C, 67.41; H, 3.77%; N, 15.72%. CAS#878609-83-7.

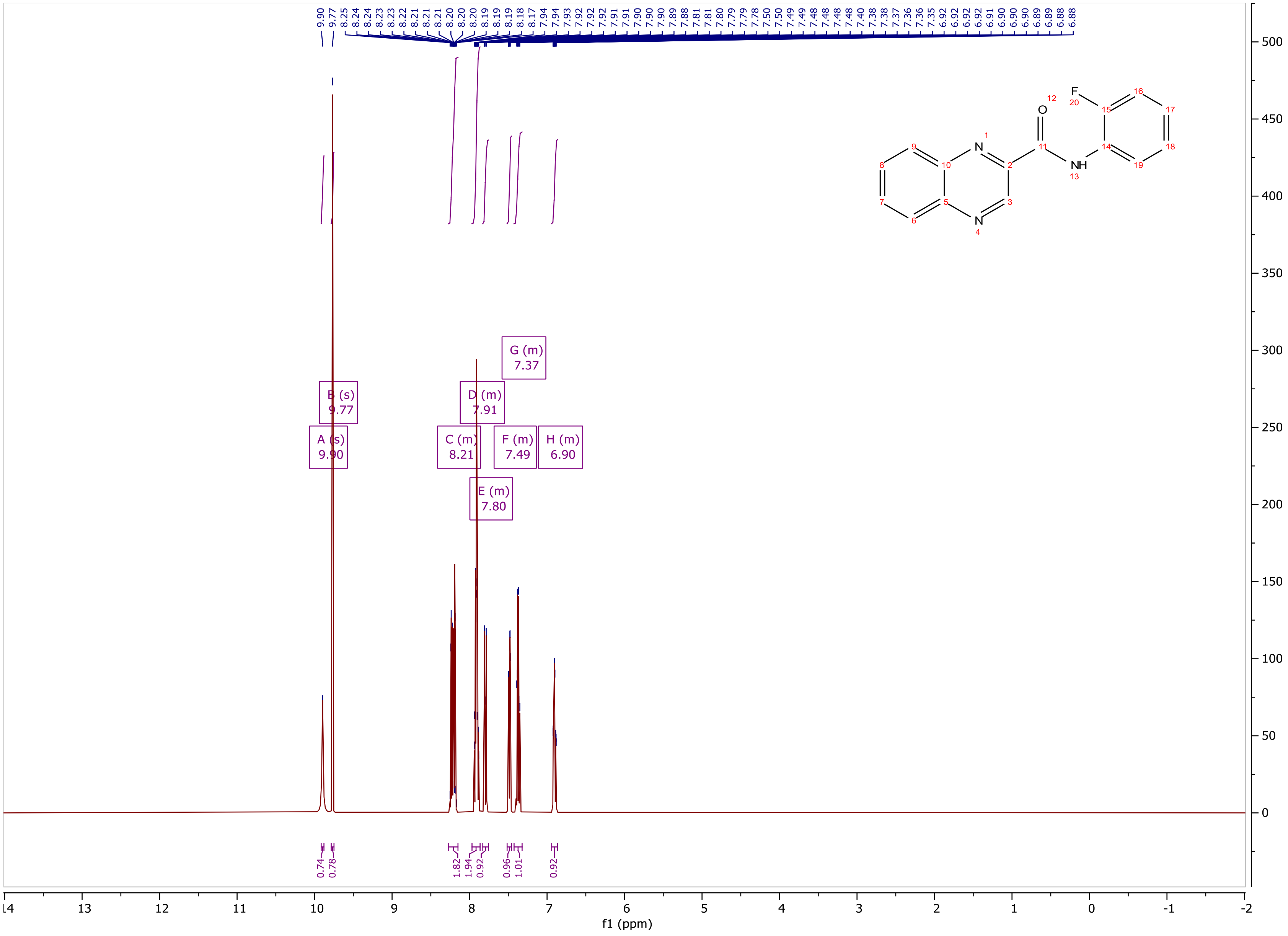

9.90
9.77
8.25
8.24
8.24
8.23
8.22
8.21
8.21
8.20
8.20
8.20
8.19
8.19
8.18
8.17
7.94
7.94
7.93
7.92
7.92
7.92
7.91
7.91
7.90
7.90
7.90
7.89
7.88
7.81
7.81
7.80
7.79
7.79
7.78
7.50
7.50
7.49
7.49
7.48
7.48
7.48
7.48
7.40
7.38
7.38
7.37
7.36
7.36
7.35
6.92
6.92
6.92
6.92
6.91
6.90
6.90
6.90
6.89
6.89
6.88
6.88
A (s)
9.90
B (s)
9.77
C (m)
8.21
D (m)
7.91
E (m)
7.80
F (m)
7.49
G (m)
7.37
H (m)
6.90
0.74
0.78
1.82
1.94
0.92
0.96
1.01
0.92
f1 (ppm)

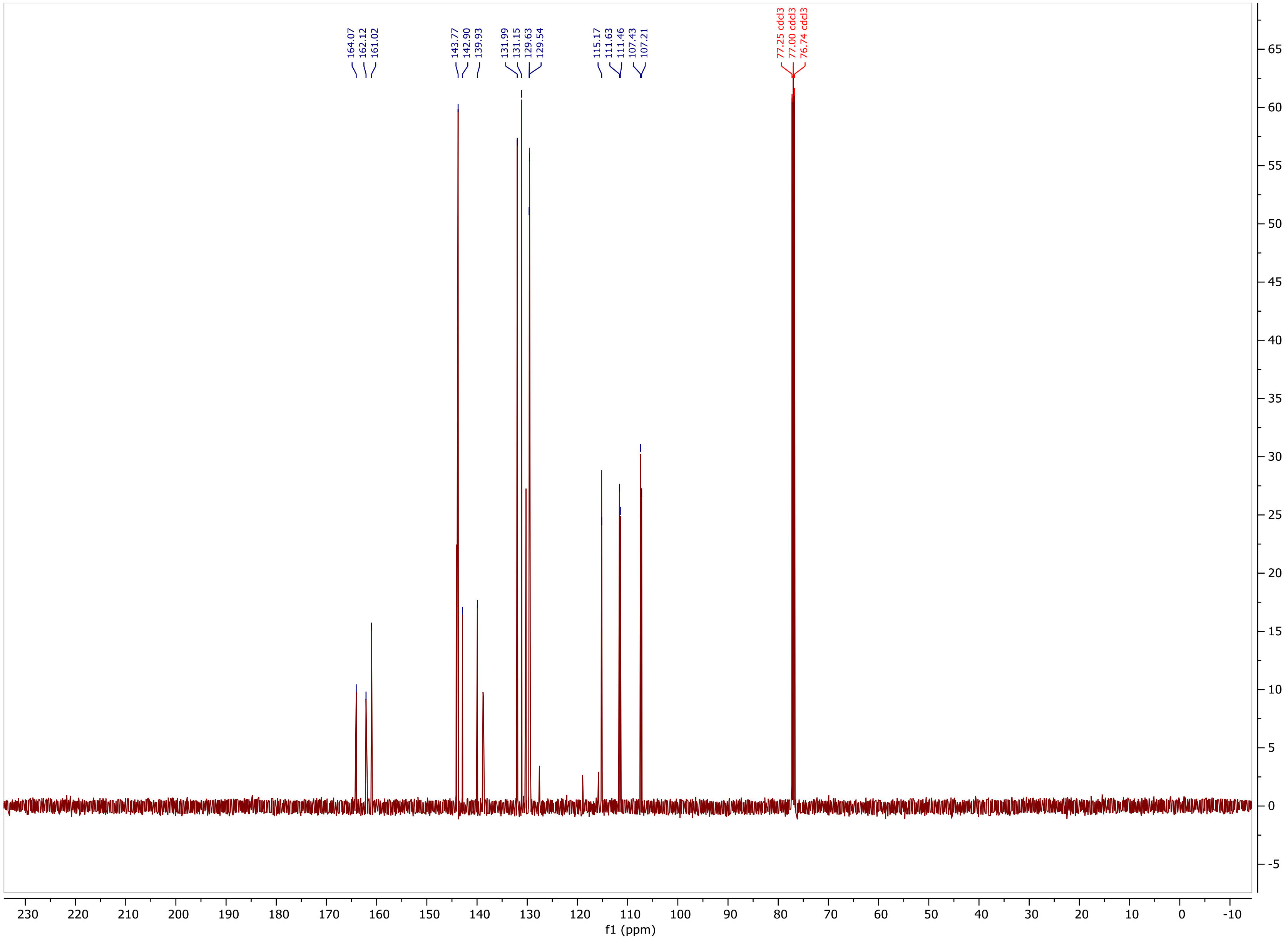
164.07
162.12
161.02
143.77
142.90
139.93
131.99
131.15
129.63
129.54
115.17
111.63
111.46
107.43
107.21
77.25 cdcl3
77.00 cdcl3
76.74 cdcl3
f1 (ppm)

Compound 8

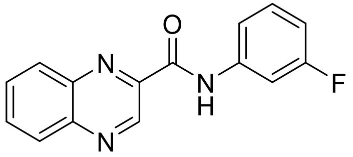

***N*-(3-fluorophenyl)quinoxaline-2-carboxamide**. M.p.: 179.5–181.2˚C. Yield: 64%. Beige powder. ^{1}H-NMR (600 MHz, Chloroform-*d*) δ 9.90 (s, 1H, amide), 9.77 (s, 1H, pyrazine), 8.25–8.18 (m, 2H, aromatic), 7.95–7.88 (m, 2H, aromatic), 7.83–7.76 (m, 1H, aromatic), 7.52–7.46 (m, 1H, aromatic), 7.43–7.32 (m, 1H, aromatic), 6.94–6.86 (m, 1H, aromatic). ^{13}C-NMR (151 MHz, DMSO-d_6) δ 163.31, 161.71, 159.09, 141.99, 139.92, 133.52, 132.29, 131.04, 130.41, 129.73, 116.89, 111.85, 111.71, 107.91, 107.73. IR (ATR-Ge, cm^{-1}): 3361 (NH, CONH), 1668 (CO, CONH), 1629, 1589, 1571 (aromatic). Calculated for $C_{15}H_{10}FN_3O$ (267.26 g/mol): C, 67.41; H, 3.77%; N, 15.72%. CAS#878609-81-5.

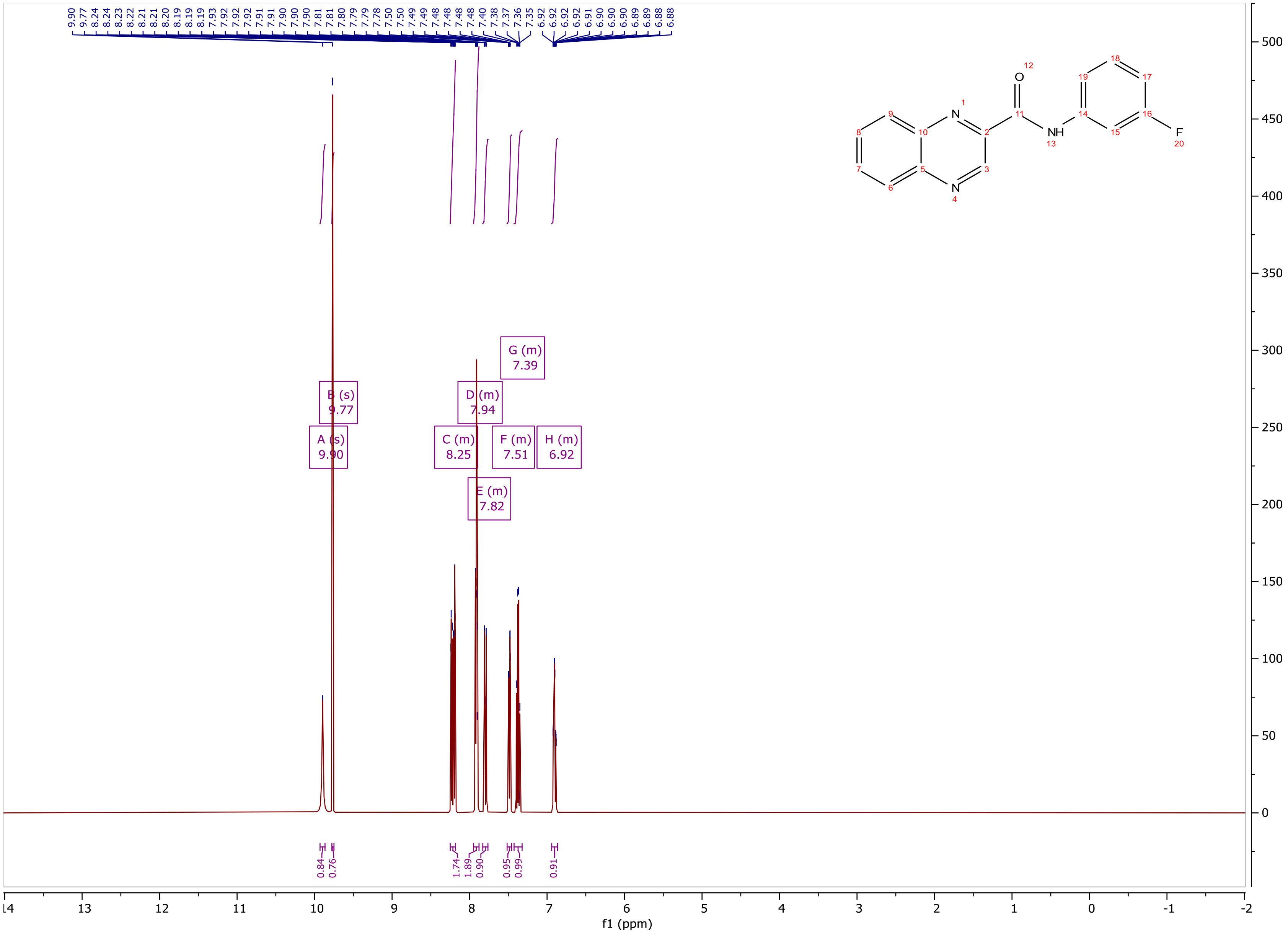

9.90
9.77
8.24
8.24
8.23
8.22
8.21
8.21
8.20
8.19
8.19
8.19
7.93
7.92
7.92
7.92
7.91
7.91
7.90
7.90
7.90
7.81
7.81
7.80
7.79
7.79
7.78
7.50
7.49
7.49
7.48
7.48
7.48
7.48
7.40
7.38
7.37
7.36
7.35
6.92
6.92
6.92
6.92
6.91
6.90
6.90
6.90
6.89
6.89
6.88
6.88
A (s)
9.90
B (s)
9.77
C (m)
8.25
D (m)
7.94
E (m)
7.82
F (m)
7.51
G (m)
7.39
H (m)
6.92
0.84
0.76
1.74
1.89
0.90
0.95
0.99
0.91
f1 (ppm)
N
N
O
NH
F
1
2
3
4
5
6
7
8
9
10
11
12
13
14
15
16
17
18
19
20

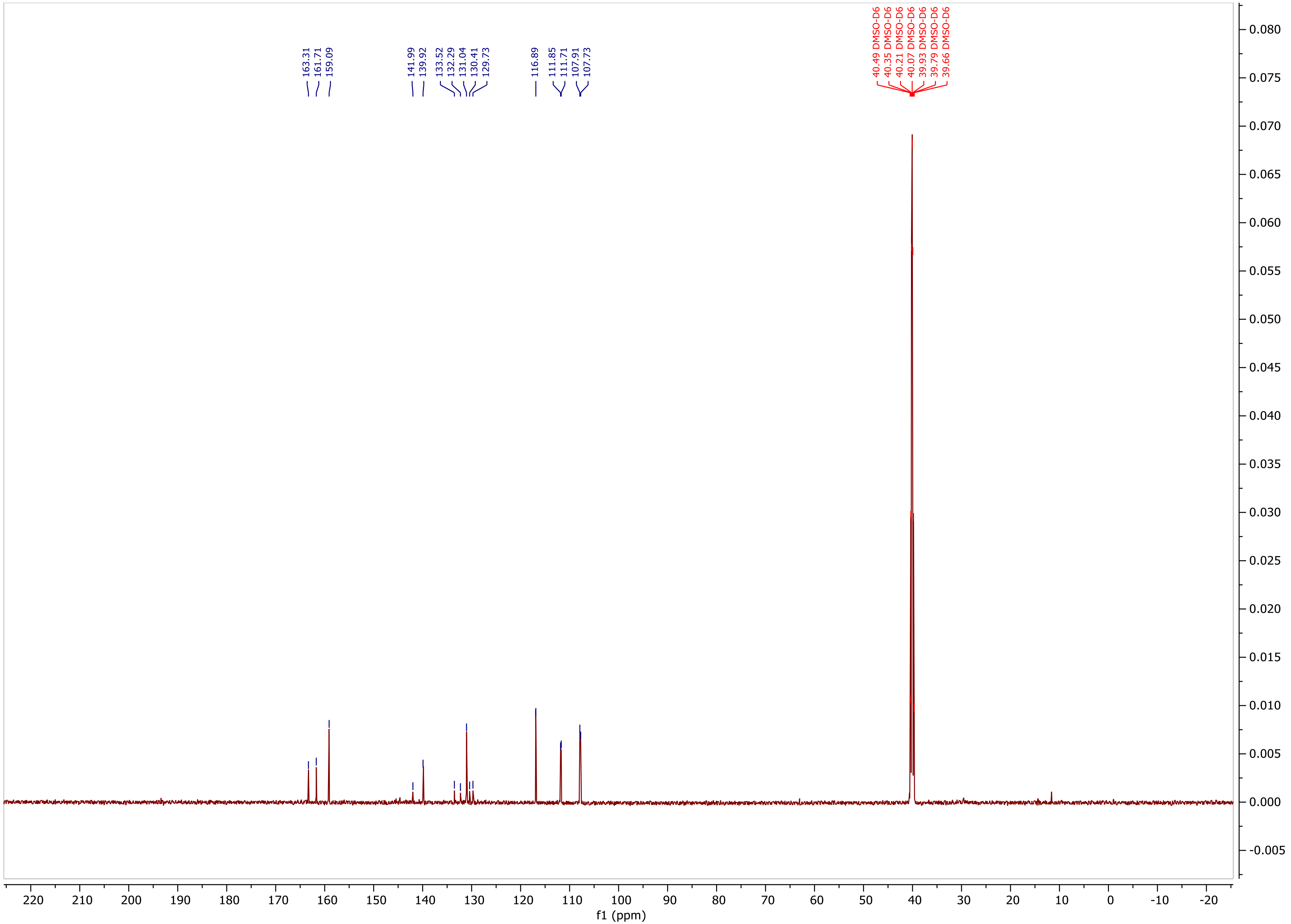

163.31
161.71
159.09
141.99
139.92
133.52
132.29
131.04
130.41
129.73
116.89
111.85
111.71
107.91
107.73
40.49 DMSO-D6
40.35 DMSO-D6
40.21 DMSO-D6
40.07 DMSO-D6
39.93 DMSO-D6
39.79 DMSO-D6
39.66 DMSO-D6
f1 (ppm)

Compound 9

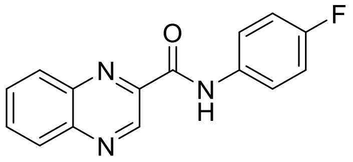

***N*-(4-fluorophenyl)quinoxaline-2-carboxamide**. M.p.: 178.8–180.1˚C. Yield: 71%. Light brown powder. ^{1}H-NMR (600 MHz, Chloroform-*d*) δ 9.83 (s, 1H, amide), 9.77 (s, 1H, pyrazine), 8.26–8.14 (m, 2H, aromatic), 7.95–7.85 (m, 2H, aromatic), 7.85–7.77 (m, 2H, aromatic), 7.16–7.09 (m, 2H, aromatic). ^{13}C-NMR (151 MHz, Chloroform-*d*) δ 160.86, 160.58, 158.63, 144.04, 143.78, 143.11, 139.95, 133.34, 131.88, 131.09, 129.60, 129.51, 121.57, 115.95, 115.77, 77.25, 77.00, 76.74. IR (ATR-Ge, cm^{-1}): 3355 (NH, CONH), 1681 (CO, CONH), 1570, 1534, 1495 (aromatic). Calculated for $C_{15}H_{10}FN_3O$ (267.26 g/mol): C, 67.41; H, 3.77%; N, 15.72%. CAS#875623-37-3.

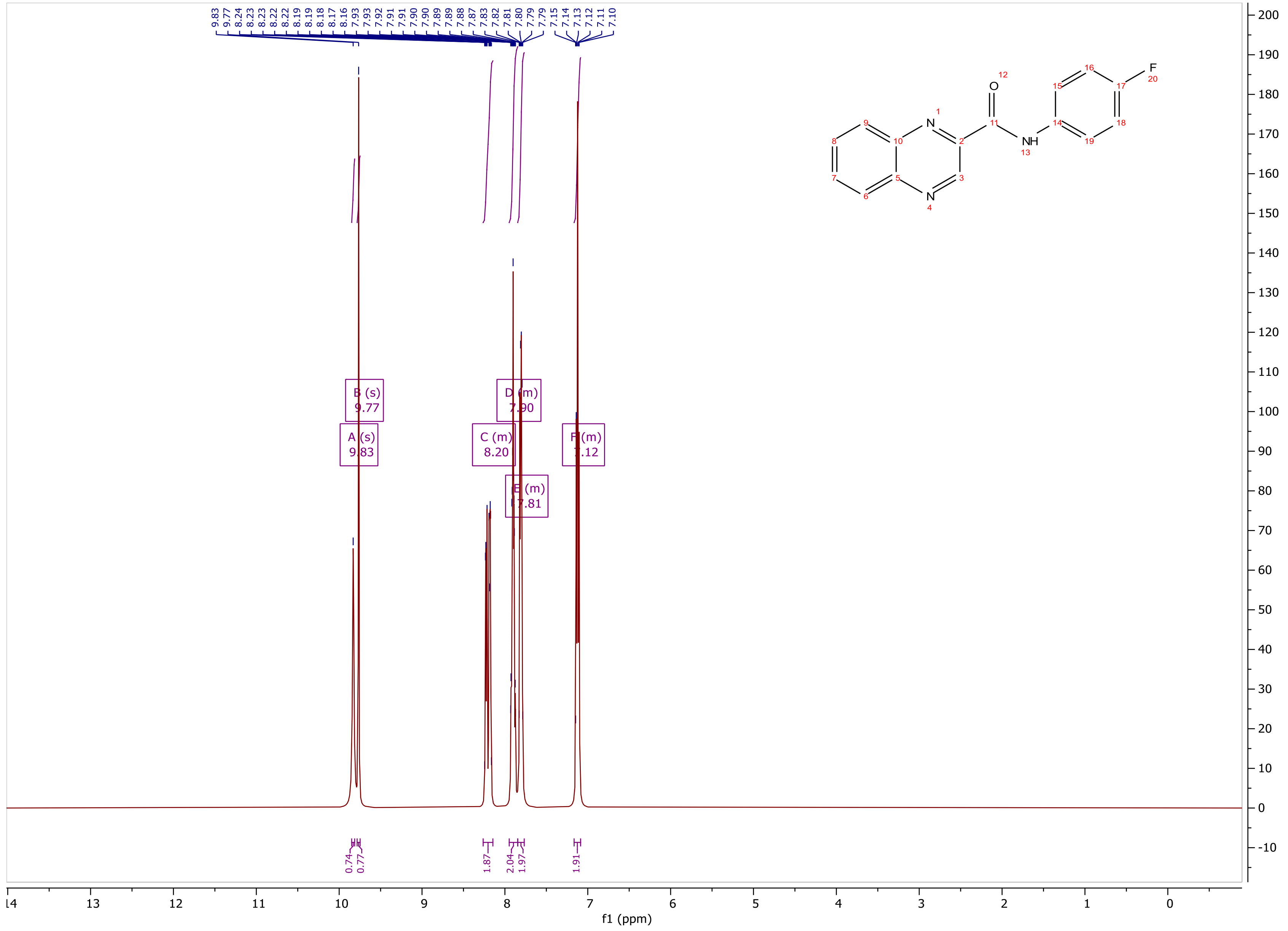

9.83
9.77
8.24
8.23
8.23
8.22
8.22
8.19
8.19
8.18
8.17
8.16
7.93
7.93
7.93
7.92
7.91
7.91
7.90
7.89
7.89
7.88
7.87
7.83
7.82
7.81
7.80
7.79
7.79
7.15
7.14
7.13
7.12
7.11
7.10
A (s)
9.83
B (s)
9.77
C (m)
8.20
D (m)
7.90
E (m)
7.81
F (m)
7.12
0.74
0.77
1.87
2.04
1.97
1.91
f1 (ppm)

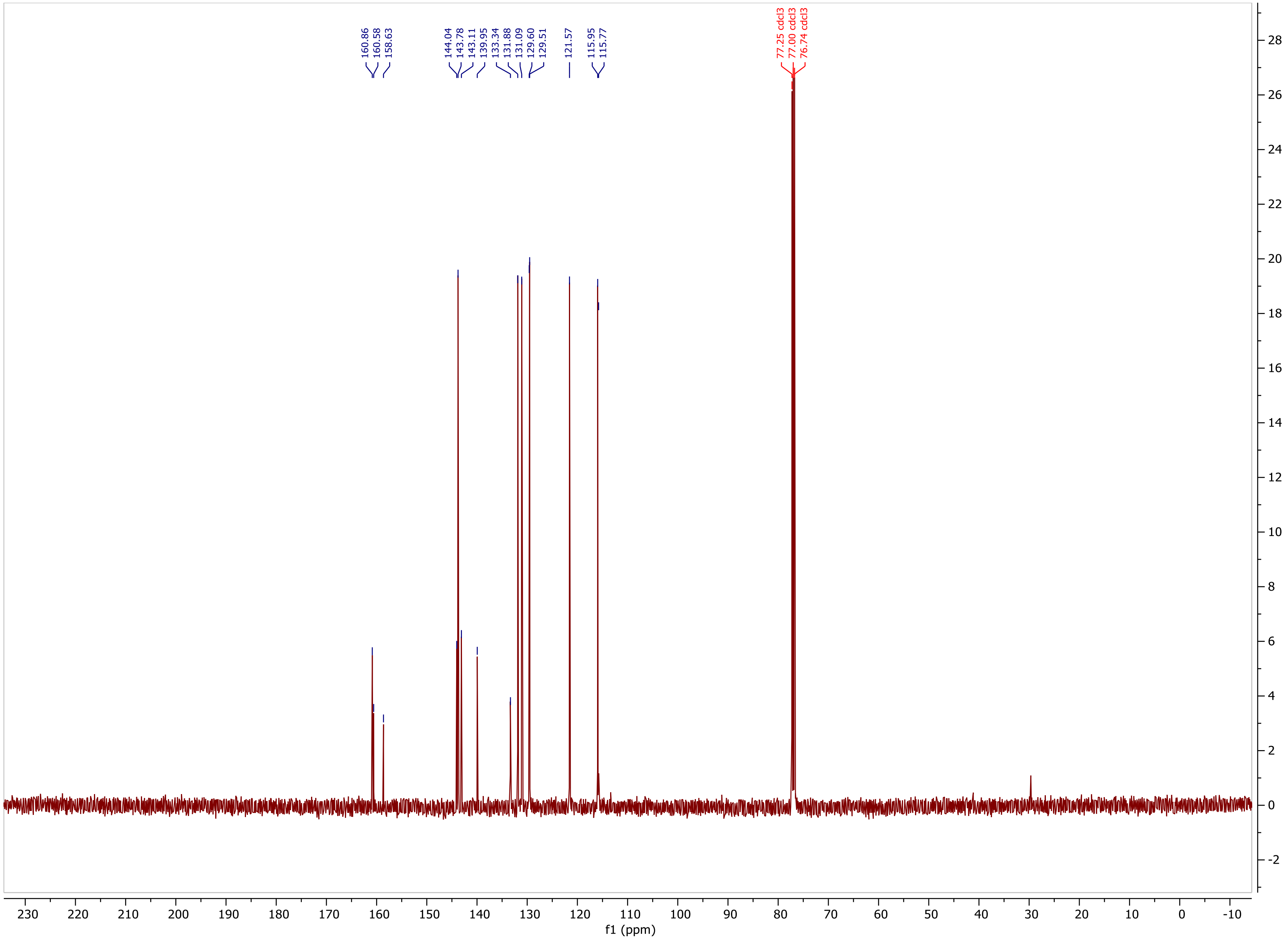
160.86
160.58
158.63
144.04
143.78
143.11
139.95
133.34
131.88
131.09
129.60
129.51
121.57
115.95
115.77
77.25 cdcl3
77.00 cdcl3
76.74 cdcl3
f1 (ppm)

Compound 10

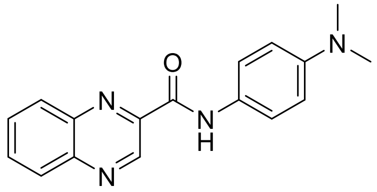

***N*-(4-(dimethylamino)phenyl)quinoxaline-2-carboxamide**. M.p.: 188–189˚C. Yield: 90%. Dark orange powder. ^{1}H-NMR (600 MHz, DMSO-d_6) δ 10.54 (s, 1H, amide), 9.52 (s, 1H, pyrazine), 8.30–8.23 (m, 1H, aromatic), 8.23–8.16 (m, 1H, aromatic), 8.02–7.95 (m, 2H, aromatic), 7.78–7.71 (m, 2H, aromatic), 6.78–6.72 (m, 2H, aromatic), 2.88 (s, 6H, methyl). ^{13}C-NMR (151 MHz, DMSO-d_6) δ 161.21, 147.78, 145.14, 144.04, 142.98, 139.84, 131.99, 131.41, 129.64, 129.25, 127.92, 121.93, 112.54, 40.50. IR (ATR-Ge, cm^{-1}): 3370 (NH, CONH), 1663 (CO, CONH), 1579, 1522, 1496 (aromatic). Calculated for $C_{17}H_{16}N_4O$ (292.34 g/mol): C, 69.85; H, 5.52%; N, 19.17%. Found: C, 70.01%; H, 5.49%; N, 19.22%. CAS# 1795178-34-5.

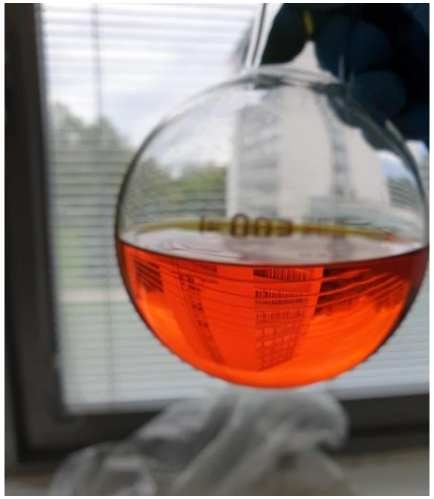

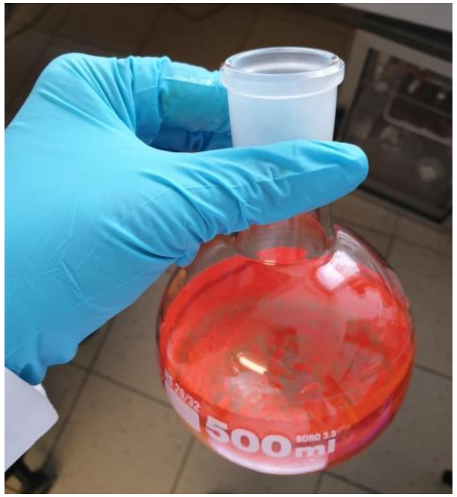

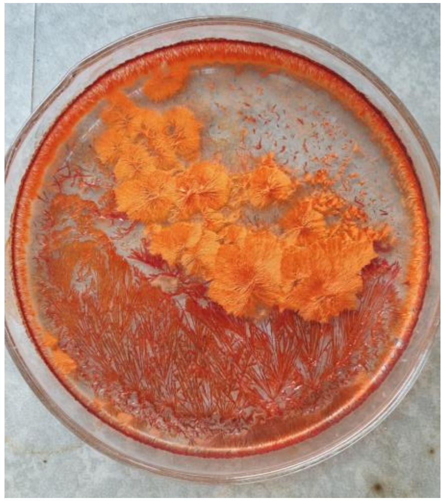

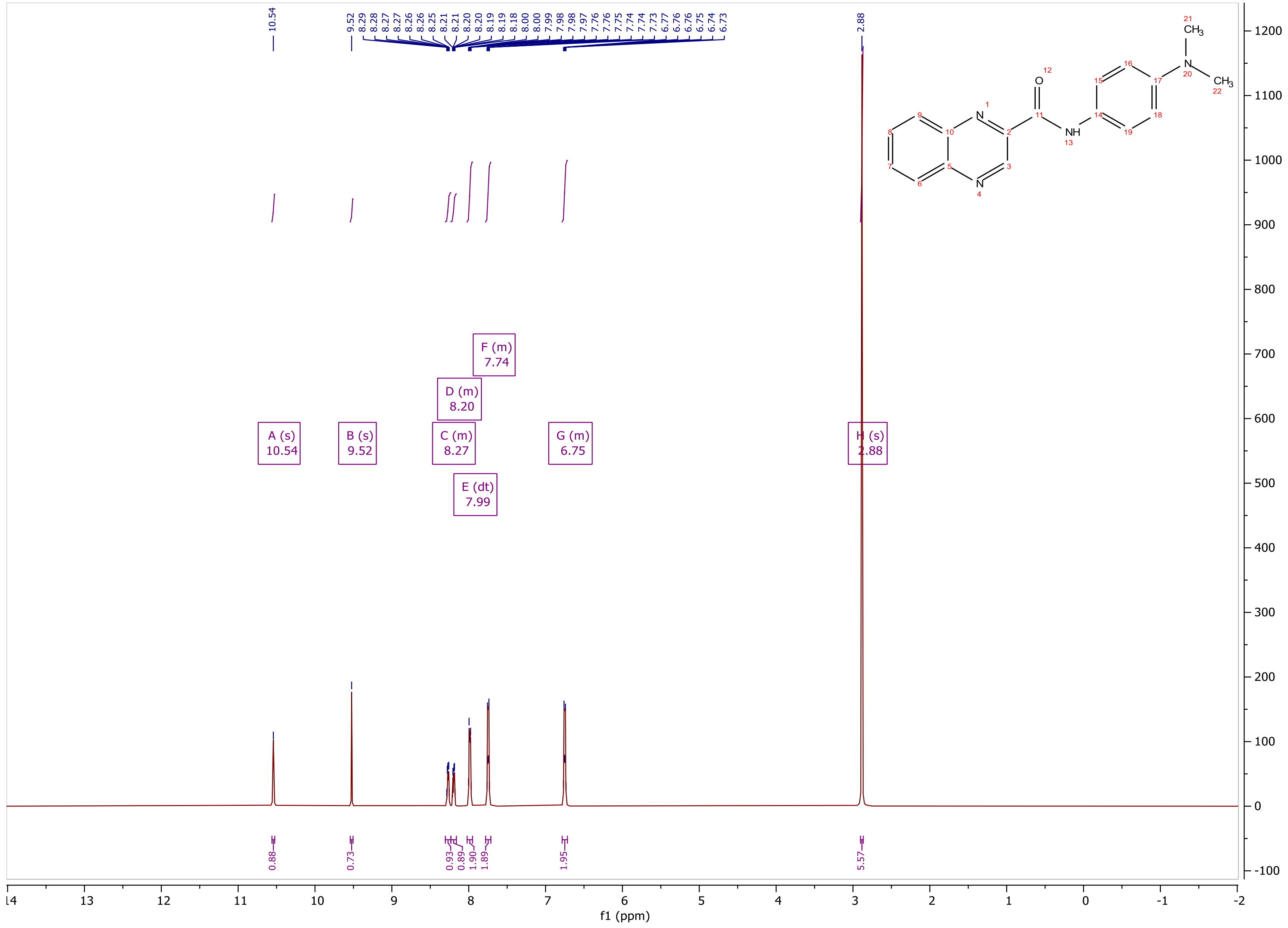

10.54
9.52
8.29 8.28 8.27 8.27 8.26 8.25 8.21 8.21 8.20 8.20 8.19 8.19 8.18 8.00 8.00 7.99 7.98 7.98 7.97 7.76 7.76 7.75 7.74 7.74 7.73 6.77 6.76 6.76 6.75 6.74 6.73
2.88
A (s) 10.54
B (s) 9.52
C (m) 8.27
D (m) 8.20
E (dt) 7.99
F (m) 7.74
G (m) 6.75
H (s) 2.88
0.88
0.73
0.93
0.89
1.90
1.89
1.95
5.57
f1 (ppm)

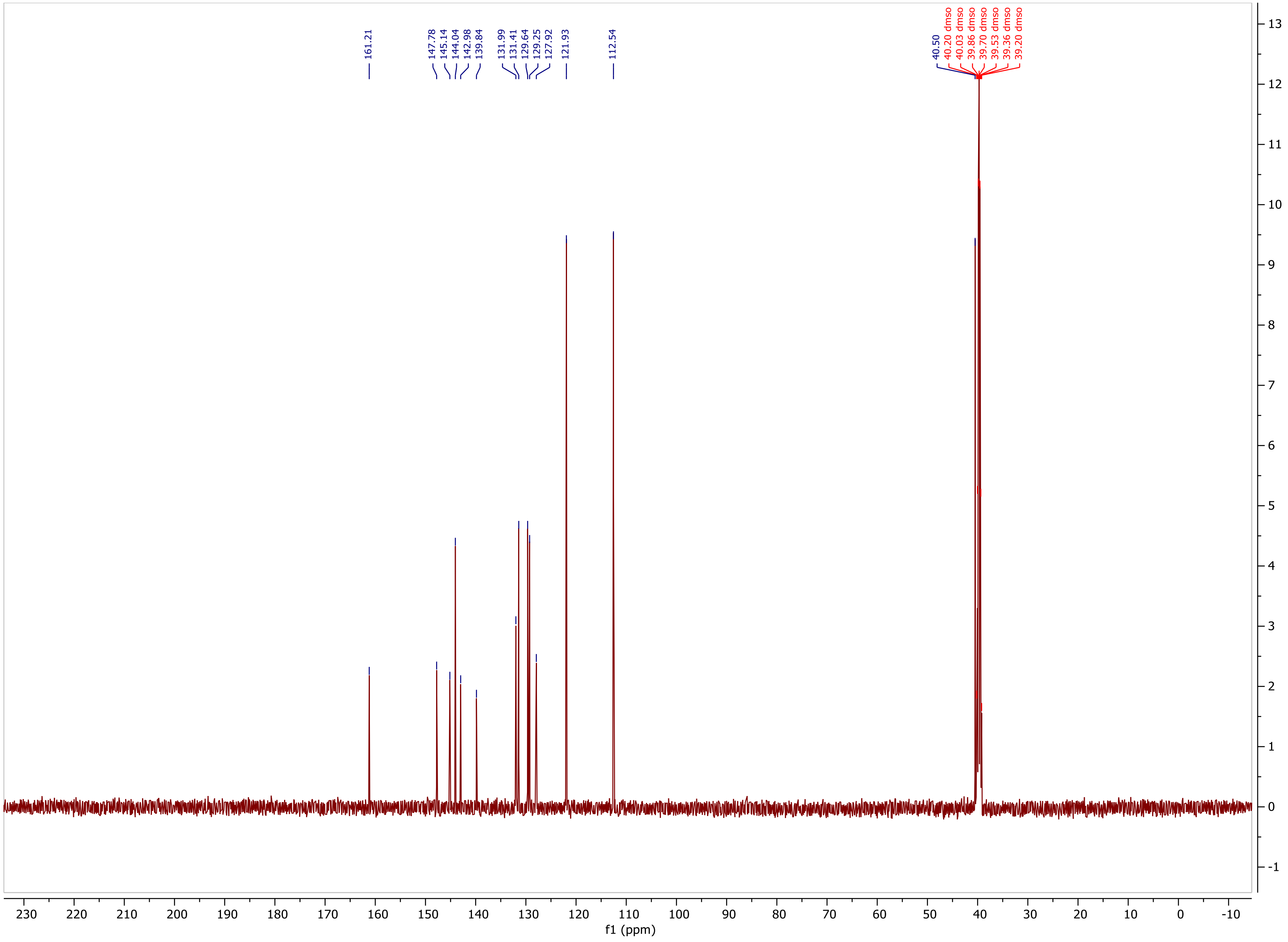
161.21
147.78
145.14
144.04
142.98
139.84
131.99
131.41
129.64
129.25
127.92
121.93
112.54
40.50
40.20 dmso
40.03 dmso
39.86 dmso
39.70 dmso
39.53 dmso
39.36 dmso
39.20 dmso
f1 (ppm)

Compound 11

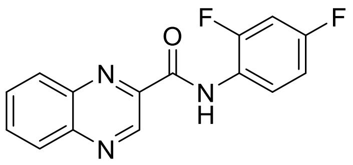

***N*-(2,4-difluorophenyl)quinoxaline-2-carboxamide**. M.p.: 178.8–179.9˚C. Yield: 47%. light brown powder. ^{1}H-NMR (600 MHz, DMSO-*d*$_6$) δ 9.59 (s, 1H, amide), 9.44 (s, 1H, pyrazine), 8.16–8.14 (m, 2H, aromatic), 7.95–7.93 (m, 2H, aromatic), 7.14–7.07 (m, 3H, aromatic). ^{13}C-NMR (151 MHz, DMSO-*d*$_6$) δ 162.17, 144.90, 144.13, 143.12, 139.83, 138.34, 132.27, 131.55, 129.74, 129.30, 128.94, 124.50, 120.75, 120.66. IR (ATR-Ge, cm^{-1}): 3352 (NH, CONH), 1694 (CO, CONH), 1556, 1538, 1520 (aromatic). Calculated for $C_{15}H_9F_2N_3O$ (285.25 g/mol): C, 63.16; H, 3.18%; N, 14.73%. CAS#878611-74-6.

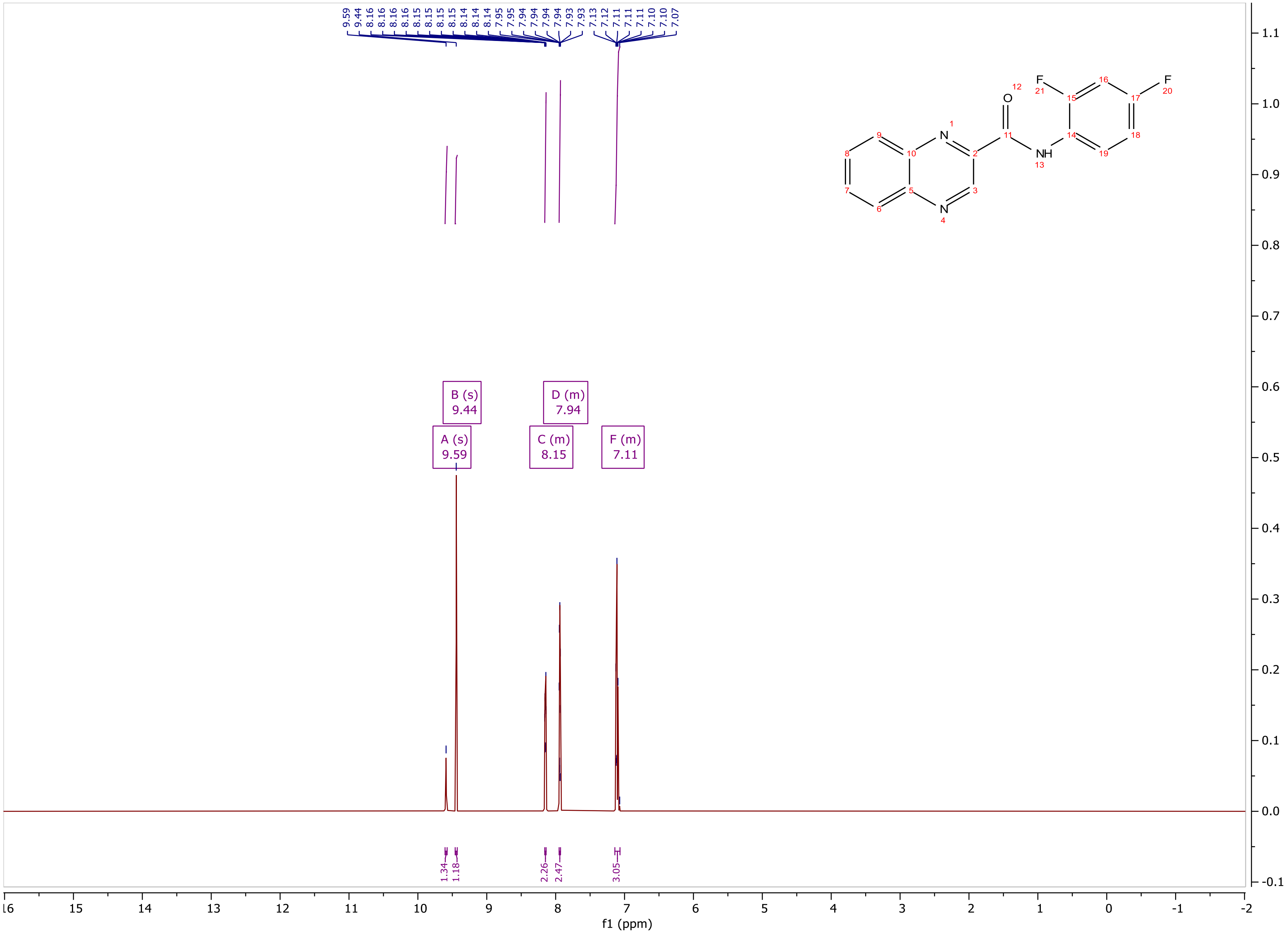

9.59
9.44
8.16
8.16
8.16
8.15
8.15
8.15
8.15
8.14
8.14
8.14
7.95
7.95
7.94
7.94
7.94
7.94
7.93
7.93
7.13
7.12
7.11
7.11
7.11
7.11
7.10
7.10
7.07
B (s)
9.44
D (m)
7.94
A (s)
9.59
C (m)
8.15
F (m)
7.11
1.34
1.18
2.26
2.47
3.05
f1 (ppm)
O
N
NH
F
F

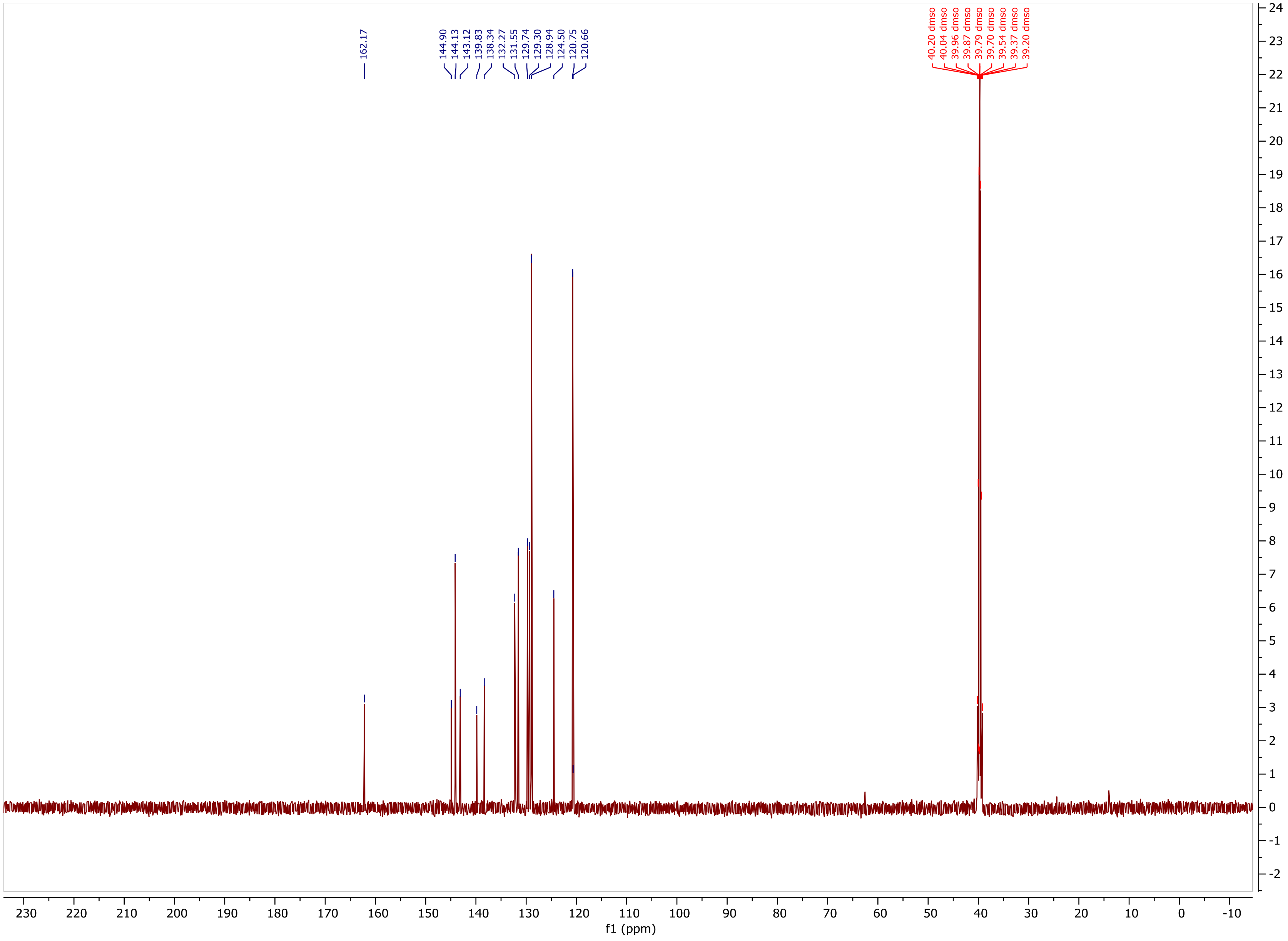
162.17
144.90
144.13
143.12
139.83
138.34
132.27
131.55
129.74
129.30
128.94
124.50
120.75
120.66
40.20 dmso
40.04 dmso
39.96 dmso
39.87 dmso
39.79 dmso
39.70 dmso
39.54 dmso
39.37 dmso
39.20 dmso
f1 (ppm)

Compound 12

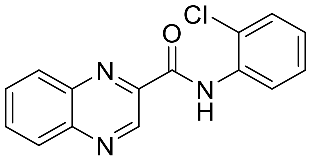

N-(2-chlorophenyl)quinoxaline-2-carboxamide. M.p.: 181.9–182.7˚C. Yield: 67%. Light beige powder. ^{1}H-NMR (600 MHz, Chloroform-*d*) δ 10.58 (s, 1H, amide), 9.77 (s, 1H, pyrazine), 8.71–8.65 (m, 1H, aromatic), 8.27–8.19 (m, 2H, aromatic), 7.95–7.86 (m, 2H, aromatic), 7.50–7.44 (m, 1H, aromatic), 7.41–7.34 (m, 1H, aromatic), 7.16–7.09 (m, 1H, aromatic). ^{13}C-NMR (151 MHz, Chloroform-*d*) δ 161.06, 144.11, 143.70, 143.18, 140.04, 134.29, 132.00, 131.06, 129.94, 129.52, 129.28, 127.86, 125.08, 123.56, 121.16. IR (ATR-Ge, cm^{-1}): 3326 (NH, CONH), 1692 (CO, CONH), 1559, 1539, 1519 (aromatic). Calculated for $C_{15}H_{10}ClN_3O$ (283.72 g/mol): C, 63.50; H, 3.55%; N, 14.81%. Found: C, 63.66%; H, 3.61%; N, 15.04%. CAS#901599-47-1.

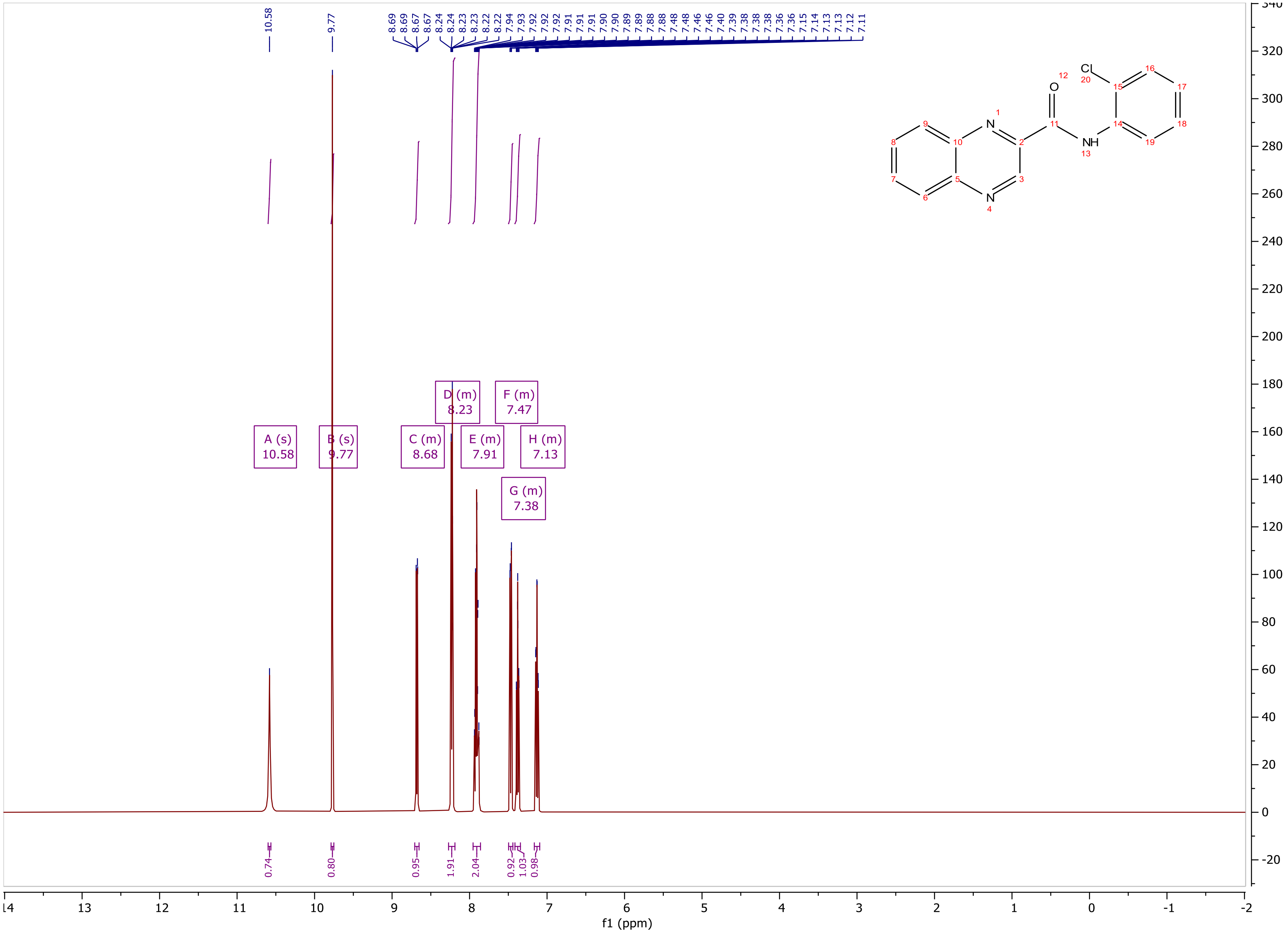

10.58
9.77
8.69
8.69
8.67
8.67
8.24
8.24
8.23
8.23
8.22
8.22
7.94
7.93
7.92
7.92
7.92
7.91
7.91
7.91
7.90
7.89
7.89
7.88
7.88
7.48
7.48
7.46
7.40
7.39
7.38
7.38
7.38
7.36
7.36
7.15
7.14
7.13
7.13
7.12
7.11
A (s) 10.58
B (s) 9.77
C (m) 8.68
D (m) 8.23
E (m) 7.91
F (m) 7.47
G (m) 7.38
H (m) 7.13
0.74
0.80
0.95
1.91
2.04
0.92
1.03
0.98
f1 (ppm)
O
Cl
N
NH

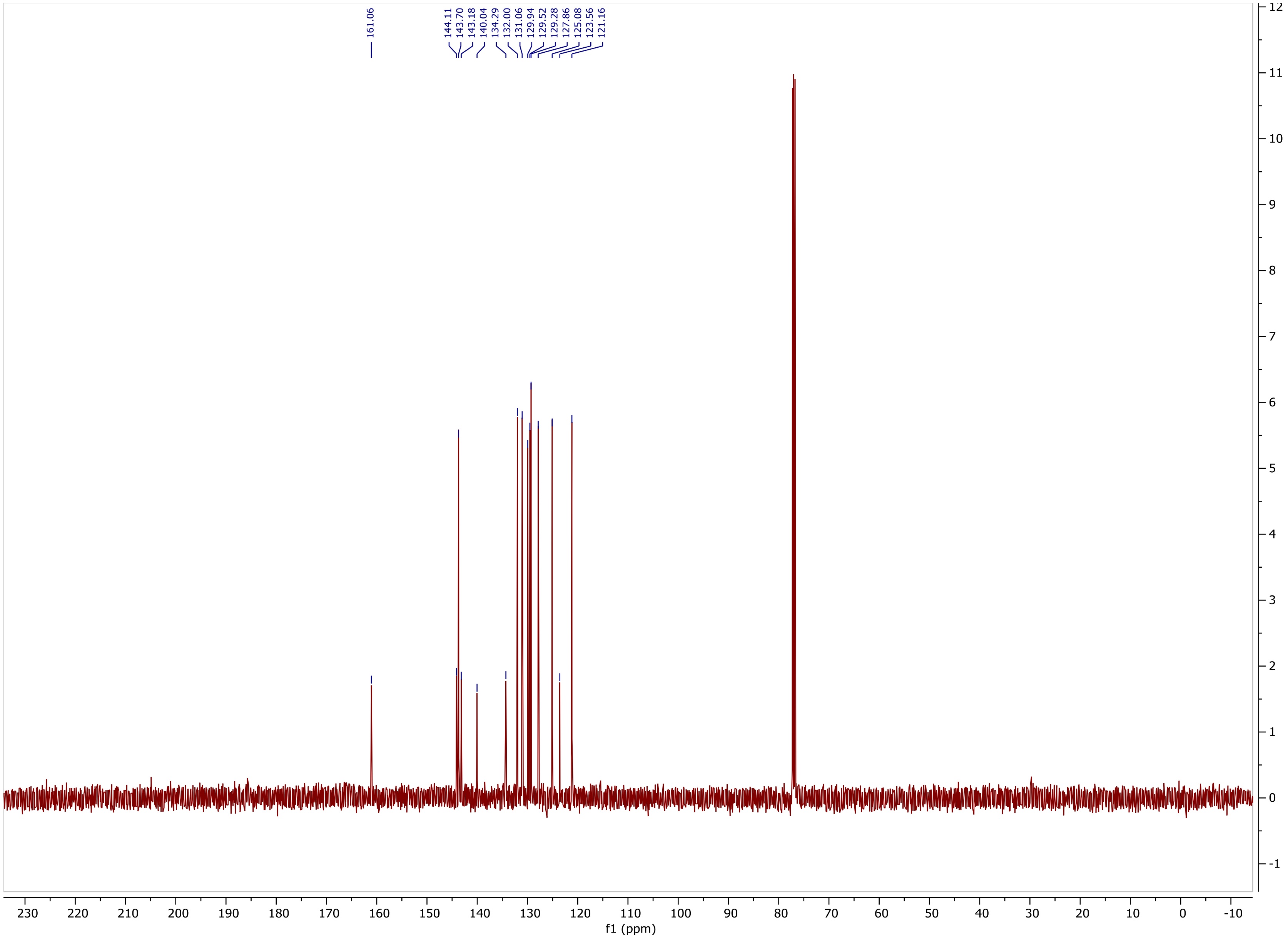
161.06
144.11
143.70
143.18
140.04
134.29
132.00
131.06
129.94
129.52
129.28
127.86
125.08
123.56
121.16
f1 (ppm)

Compound 13

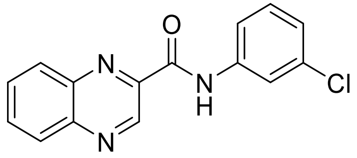

N-(3-chlorophenyl)quinoxaline-2-carboxamide. M.p.: 171.7–172.8˚C {in literature M.p. 166–168˚C [1]}. Yield: 69%. light beige powder. ^{1}H-NMR (500 MHz, Chloroform-*d*) δ 9.87 (s, 1H, amide), 9.77 (s, 1H, pyrazine), 8.27–8.16 (m, 2H, aromatic), 7.97–7.87 (m, 3H, aromatic), 7.76–7.68 (m, 1H, aromatic), 7.39–7.32 (m, 1H, aromatic), 7.21–7.15 (m, 1H, aromatic). ^{13}C-NMR (151 MHz, Chloroform-*d*) δ 161.02, 144.14, 143.77, 142.88, 139.94, 138.41, 134.89, 132.01, 131.18, 130.18, 129.64, 129.55, 124.85, 119.92, 117.82. IR (ATR-Ge, cm^{-1}): 3360 (NH, CONH), 1698 (CO, CONH), 1559, 1533, 1525 (aromatic). Calculated for $C_{15}H_{10}ClN_3O$ (283.72 g/mol): C, 63.50; H, 3.55%; N, 14.81%. Found: C, 63.45%; H, 3.52%; N, 14.99%. CAS#901599-57-3.

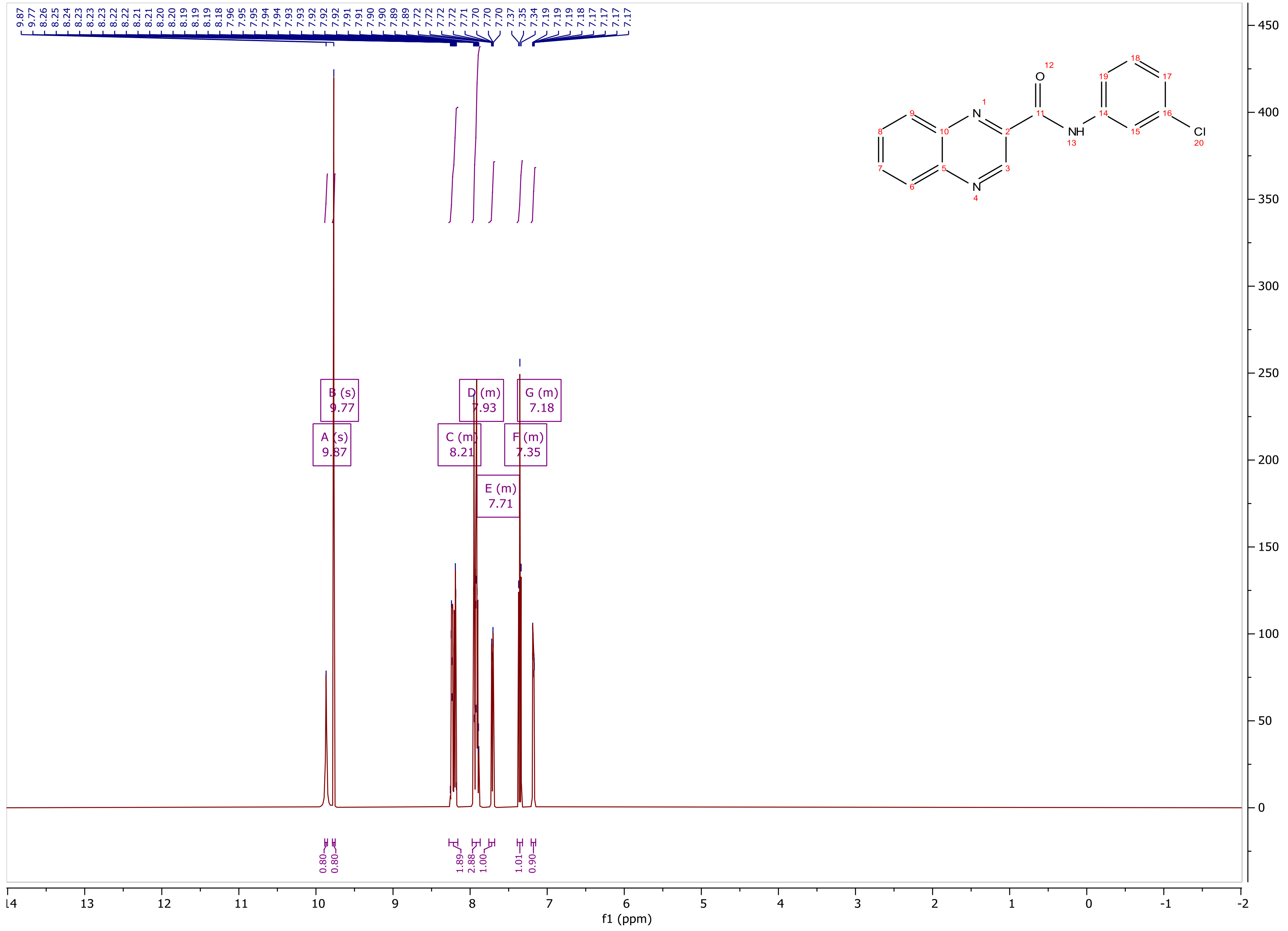

9.87 9.77 8.26 8.25 8.24 8.23 8.23 8.23 8.22 8.22 8.21 8.21 8.20 8.20 8.19 8.19 8.18 7.96 7.95 7.95 7.94 7.94 7.93 7.93 7.92 7.92 7.92 7.92 7.91 7.91 7.90 7.90 7.89 7.89 7.72 7.72 7.72 7.72 7.71 7.70 7.70 7.70 7.37 7.35 7.34 7.19 7.19 7.19 7.18 7.17 7.17 7.17 7.17
A (s) 9.87
B (s) 9.77
C (m) 8.21
D (m) 7.93
E (m) 7.71
F (m) 7.35
G (m) 7.18
0.80 0.80 1.89 2.88 1.00 1.01 0.90
NH
Cl
O
N
f1 (ppm)

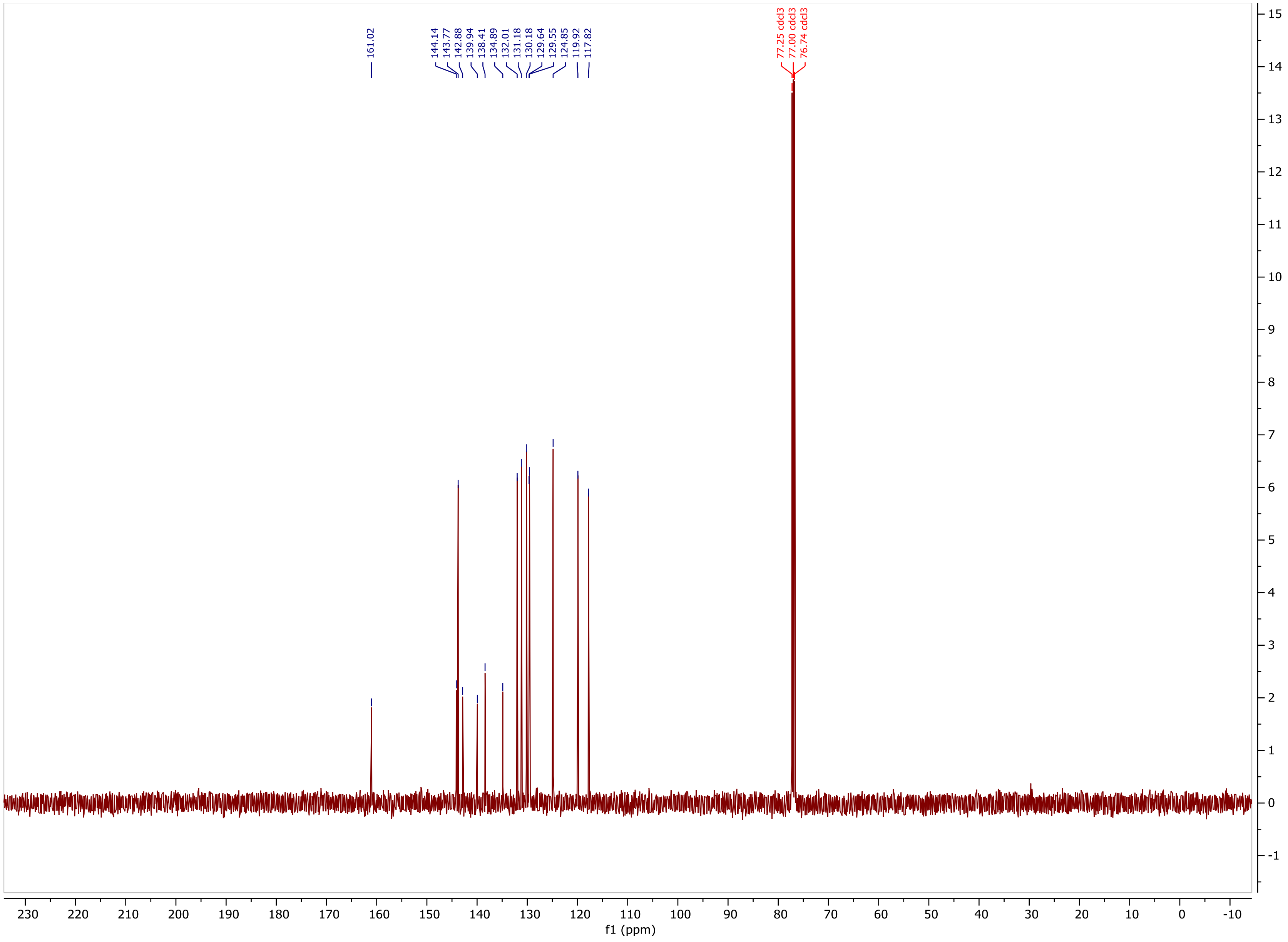
161.02
144.14
143.77
142.88
139.94
138.41
134.89
132.01
131.18
130.18
129.64
129.55
124.85
119.92
117.82
77.25 cdcl3
77.00 cdcl3
76.74 cdcl3
f1 (ppm)

Compound 14

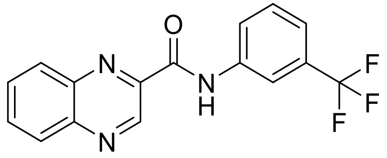

N-(3-(trifluoromethyl)phenyl)quinoxaline-2-carboxamide. M.p.: 198.2–200.2˚C. Yield: 88%. White solid powder. ^{1}H-NMR (500 MHz, Chloroform-*d*) δ 9.96 (s, 1H, amide), 9.76 (s, 1H, pyrazine), 8.26–8.15 (m, 2H, aromatic), 8.15–8.11 (m, 1H, aromatic), 8.08–8.01 (m, 1H, aromatic), 7.96–7.86 (m, 2H, aromatic), 7.58–7.51 (m, 1H, aromatic), 7.48–7.42 (m, 1H, aromatic). ^{13}C-NMR (126 MHz, DMSO-d_6): δ 163.06, 144.84, 144.52, 143.56, 140.17, 139.59, 132.79, 132.00 (q, J = 32.5 Hz), 130.50, 130.11, 129.90, 129.70, 125.57 (q, *J* = 272.4 Hz), 123.77, 121.11 (q, *J* = 3.9 Hz), 117.39 (q, *J* = 4.0 Hz). IR (ATR-Ge, cm^{-1}): 3347 (NH, CONH), 1687 (CO, CONH), 1603, 1574, 1539 (aromatic). Calculated for $C_{16}H_{10}F_3N_3O$ (317.27 g/mol): C, 60.57%; H, 3.18%; N, 13.24%. CAS# 689265-58-5.

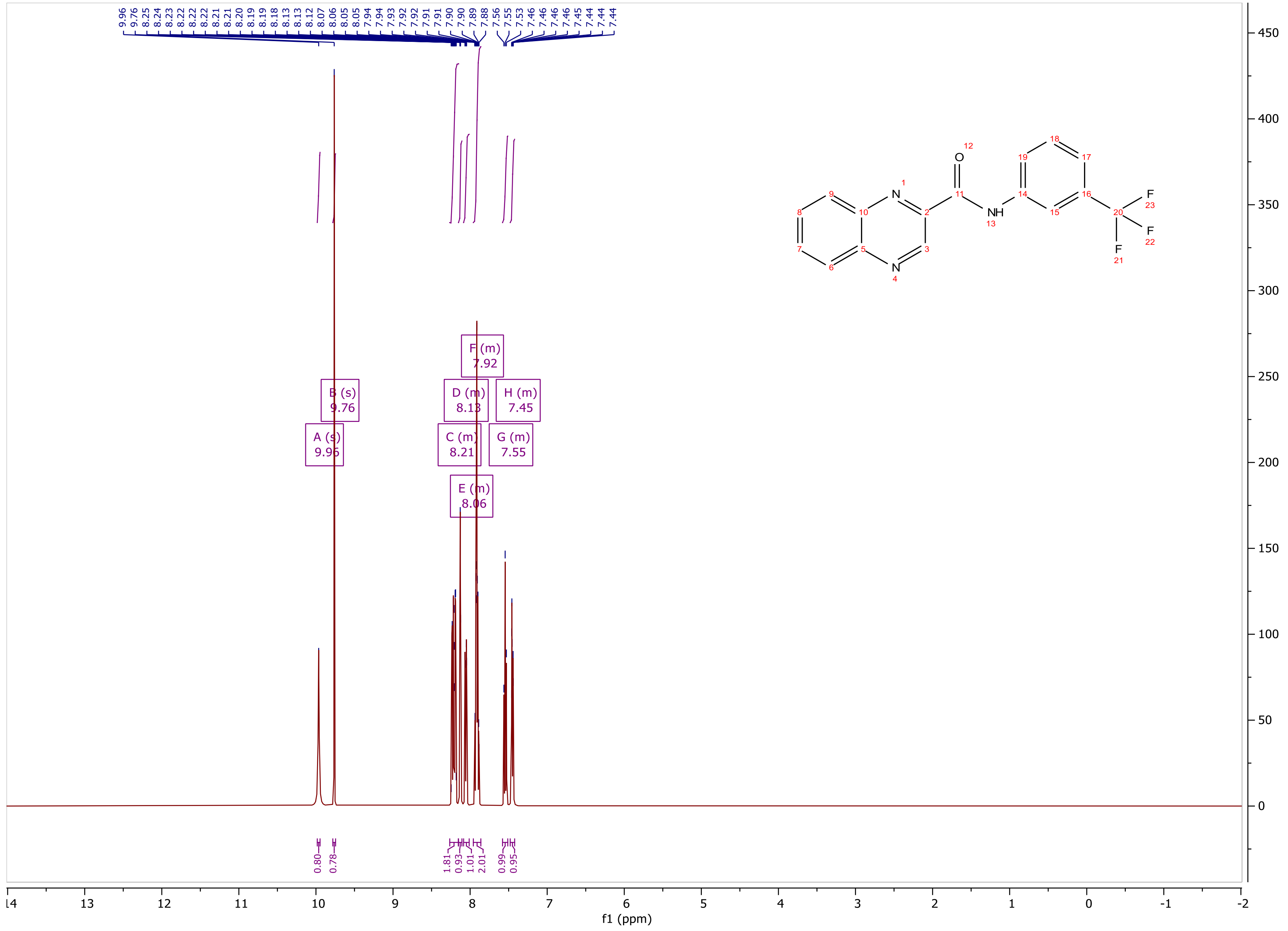

9.96
9.76
8.25
8.24
8.23
8.22
8.22
8.22
8.21
8.21
8.20
8.19
8.19
8.18
8.13
8.13
8.12
8.07
8.06
8.05
8.05
7.94
7.94
7.93
7.92
7.92
7.91
7.90
7.90
7.89
7.88
7.56
7.55
7.53
7.46
7.46
7.46
7.45
7.44
7.44
7.44
A (s) 9.96
B (s) 9.76
C (m) 8.21
D (m) 8.13
E (m) 8.06
F (m) 7.92
G (m) 7.55
H (m) 7.45
0.80
0.78
1.81
0.93
1.01
2.01
0.99
0.95
f1 (ppm)
O
N
NH
F
F
F

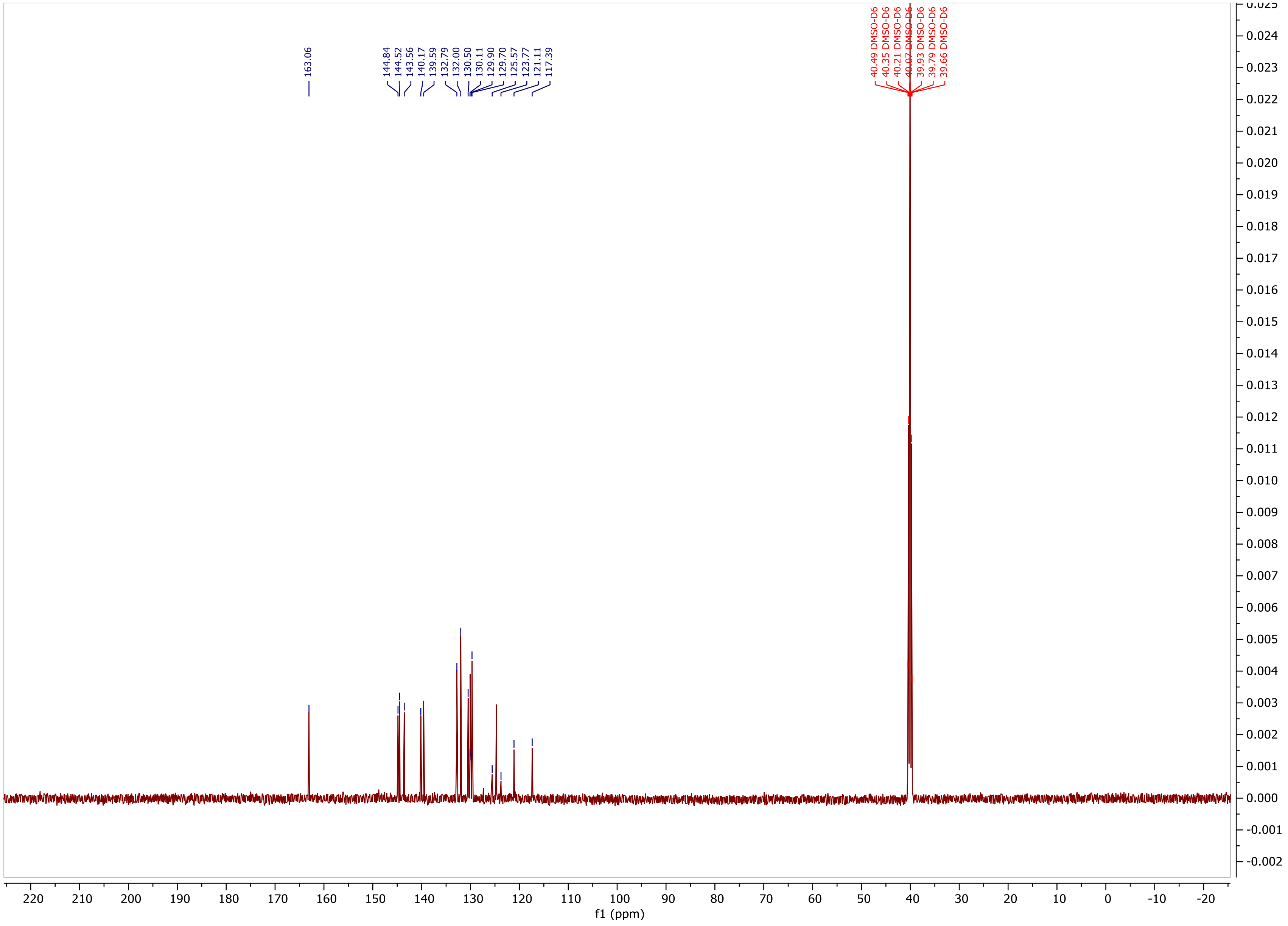

163.06
144.84
144.52
143.56
140.17
139.59
132.79
132.00
130.50
130.11
129.90
129.70
125.57
123.77
121.11
117.39
40.49 DMSO-D6
40.35 DMSO-D6
40.21 DMSO-D6
40.07 DMSO-D6
39.93 DMSO-D6
39.79 DMSO-D6
39.66 DMSO-D6
f1 (ppm)

Compound 15

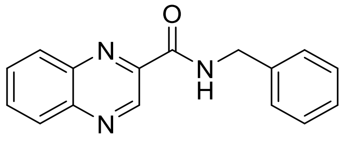

***N*-benzylquinoxaline-2-carboxamide**. M.p.: 202.2–204.8˚C. Yield: 61%. White crystals. ^{1}H-NMR (500 MHz, Chloroform-*d*) δ 9.73 (s, 1H, pyrazine), 8.34 (t, *J* = 6.6 Hz, 1H, amide), 8.22–8.17 (m, 1H, aromatic), 8.11–8.06 (m, 1H, aromatic), 7.90–7.79 (m, 2H, aromatic), 7.46–7.36 (m, 4H, aromatic), 7.36–7.28 (m, 1H, aromatic), 4.76 (d, *J* = 6.1 Hz, 2H, methylene). ^{13}C-NMR (126 MHz, DMSO-d_6) δ 163.14, 147.69, 145.01, 143.76, 143.53, 139.39, 128.42, 127.53, 126.97, 42.54. IR (ATR-Ge, cm^{-1}): 3370 (NH, CONH), 2931 (CH_2), 1670 (CO, CONH), 1585, 1524, 1516 (aromatic). Calculated for $C_{16}H_{13}N_3O$ (263.30 g/mol): C, 72.99%; H, 4.98%; N, 15.96%. Found: C, 72.62%; H, 4.88%; N, 15.83%. CAS# 7066-32-2.

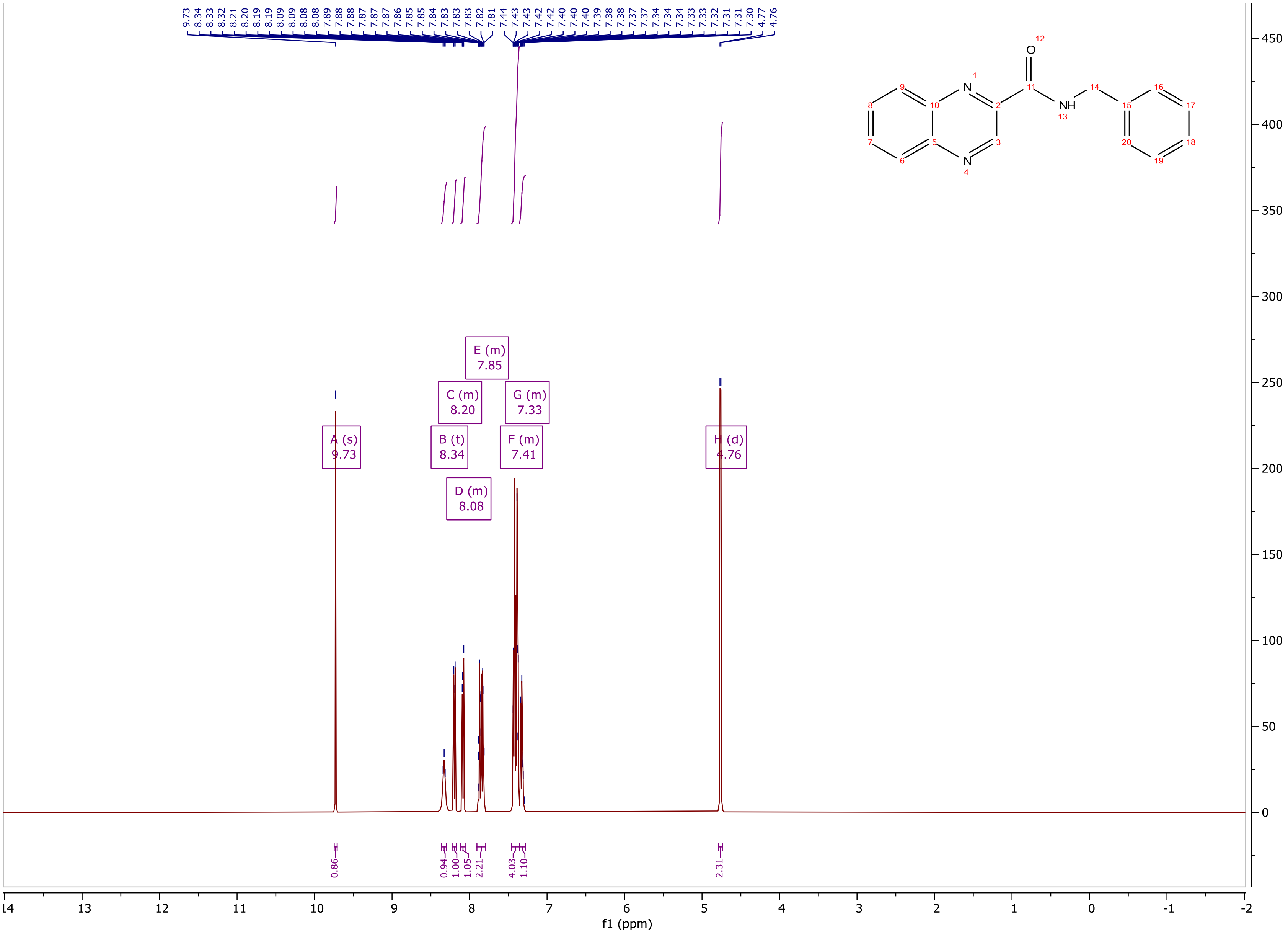

A (s) 9.73
B (t) 8.34
C (m) 8.20
D (m) 8.08
E (m) 7.85
F (m) 7.41
G (m) 7.33
H (d) 4.76
0.86
0.94
1.00
1.05
2.21
4.03
1.10
2.31
f1 (ppm)
O
N
NH
N

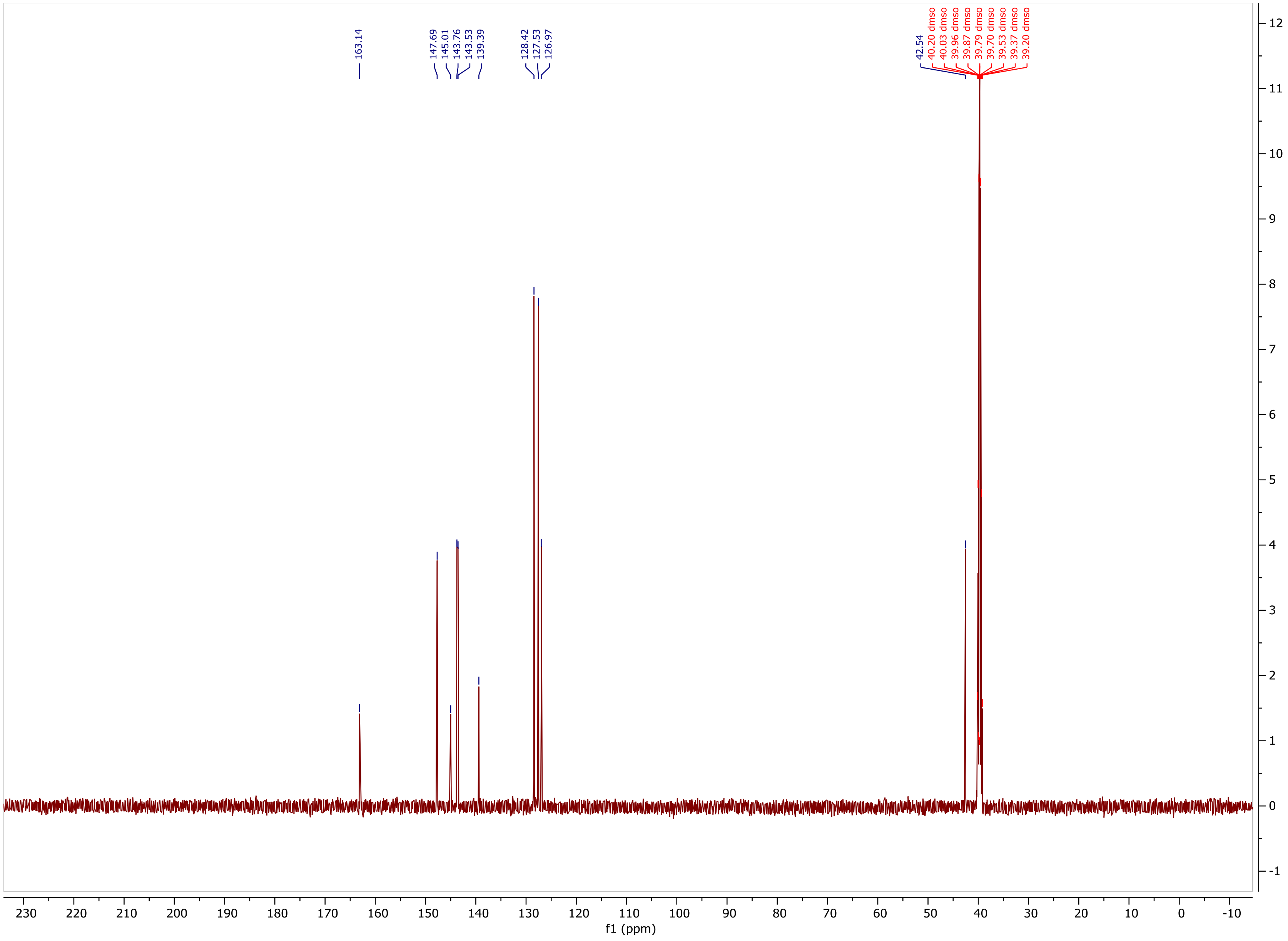
163.14
147.69
145.01
143.76
143.53
139.39
128.42
127.53
126.97
42.54
40.20 dmso
40.03 dmso
39.96 dmso
39.87 dmso
39.79 dmso
39.70 dmso
39.53 dmso
39.37 dmso
39.20 dmso
f1 (ppm)

Compound 16

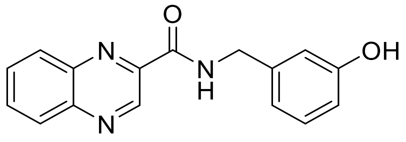

N-(3-hydroxybenzyl)quinoxaline-2-carboxamide. M.p.: 225.9–226.7˚C. Yield: 57%. Brown powder. ^{1}H-NMR (600 MHz, DMSO-d_6) δ 9.55 (t, J = 6.4 Hz, 1H, amide), 9.49 (s, 1H, pyrazine), 9.33 (s, 1H, hydroxy), 8.23–8.16 (m, 2H, aromatic), 8.02–7.94 (m, 2H, aromatic), 7.14–7.08 (m, 1H, aromatic), 6.82–6.77 (m, 2H, aromatic), 6.66–6.60 (m, 1H, aromatic), 4.49 (d, J = 6.4 Hz, 2H, methylene). ^{13}C-NMR (151 MHz, DMSO-d_6) δ 163.33, 157.57, 144.57, 143.96, 143.16, 140.77, 140.00, 132.09, 131.51, 129.61, 129.47, 129.31, 118.26, 114.46, 114.02, 42.62. IR (ATR-Ge, cm^{-1}): 3356 (NH, CONH), 2923 (CH_2), 1668 (CO, CONH), 1559, 1508, 1495 (aromatic). Calculated for $C_{16}H_{13}N_3O_2$ (279.30 g/mol): C, 68.81; H, 4.69%; N, 15.05%. Found: C, 68.70%; H, 4.68%; N, 15.10%. CAS# 1914001-45-8.

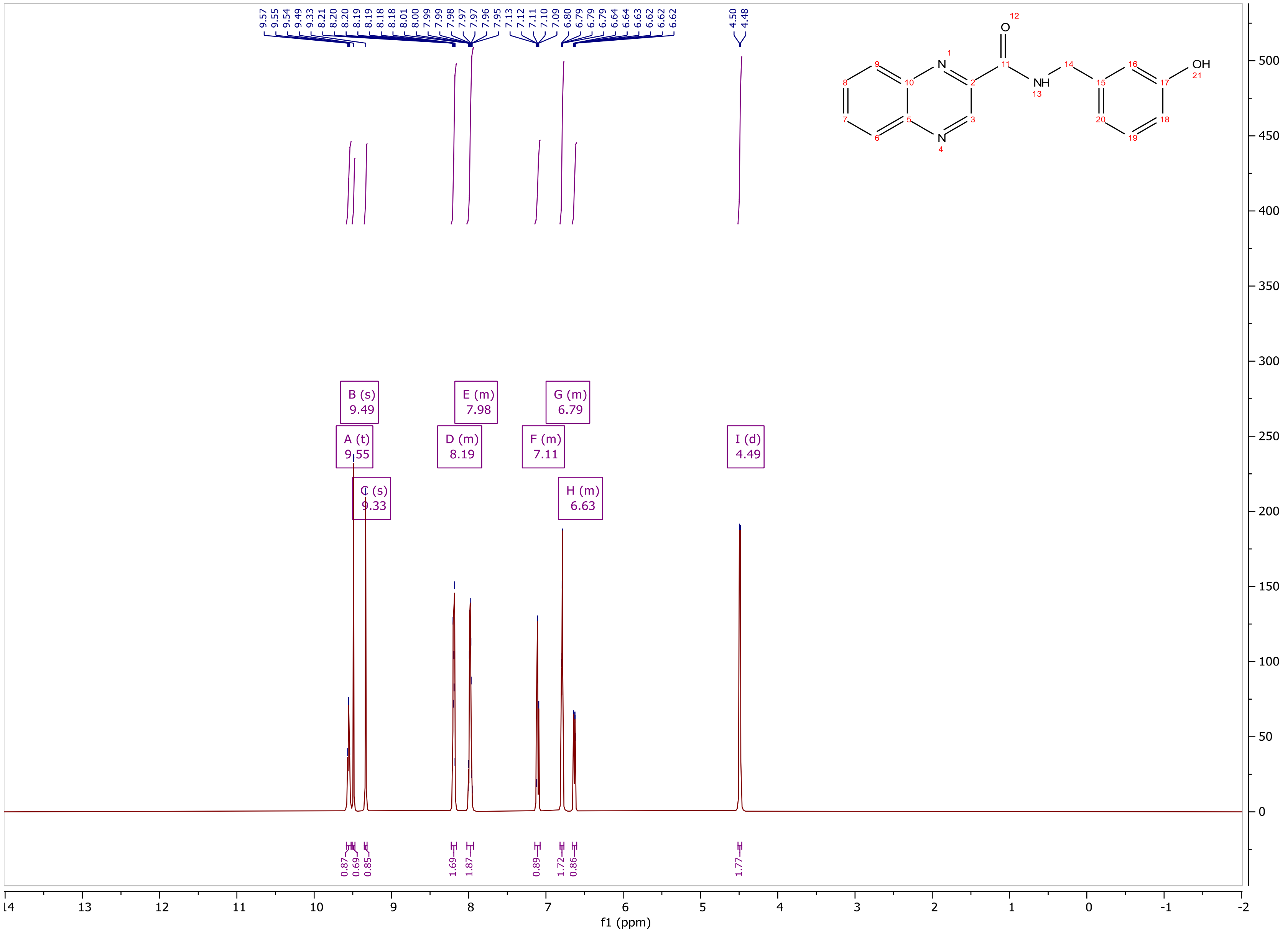
9.57
9.55
9.54
9.49
9.33
8.21
8.20
8.20
8.19
8.19
8.18
8.18
8.01
8.00
7.99
7.99
7.98
7.97
7.97
7.96
7.95
7.13
7.12
7.11
7.10
7.09
6.80
6.79
6.79
6.79
6.64
6.64
6.63
6.62
6.62
6.62
4.50
4.48
B (s)
9.49
A (t)
9.55
C (s)
9.33
E (m)
7.98
D (m)
8.19
G (m)
6.79
F (m)
7.11
H (m)
6.63
I (d)
4.49
0.87
0.69
0.85
1.69
1.87
0.89
1.72
0.86
1.77
f1 (ppm)
O
N
NH
OH

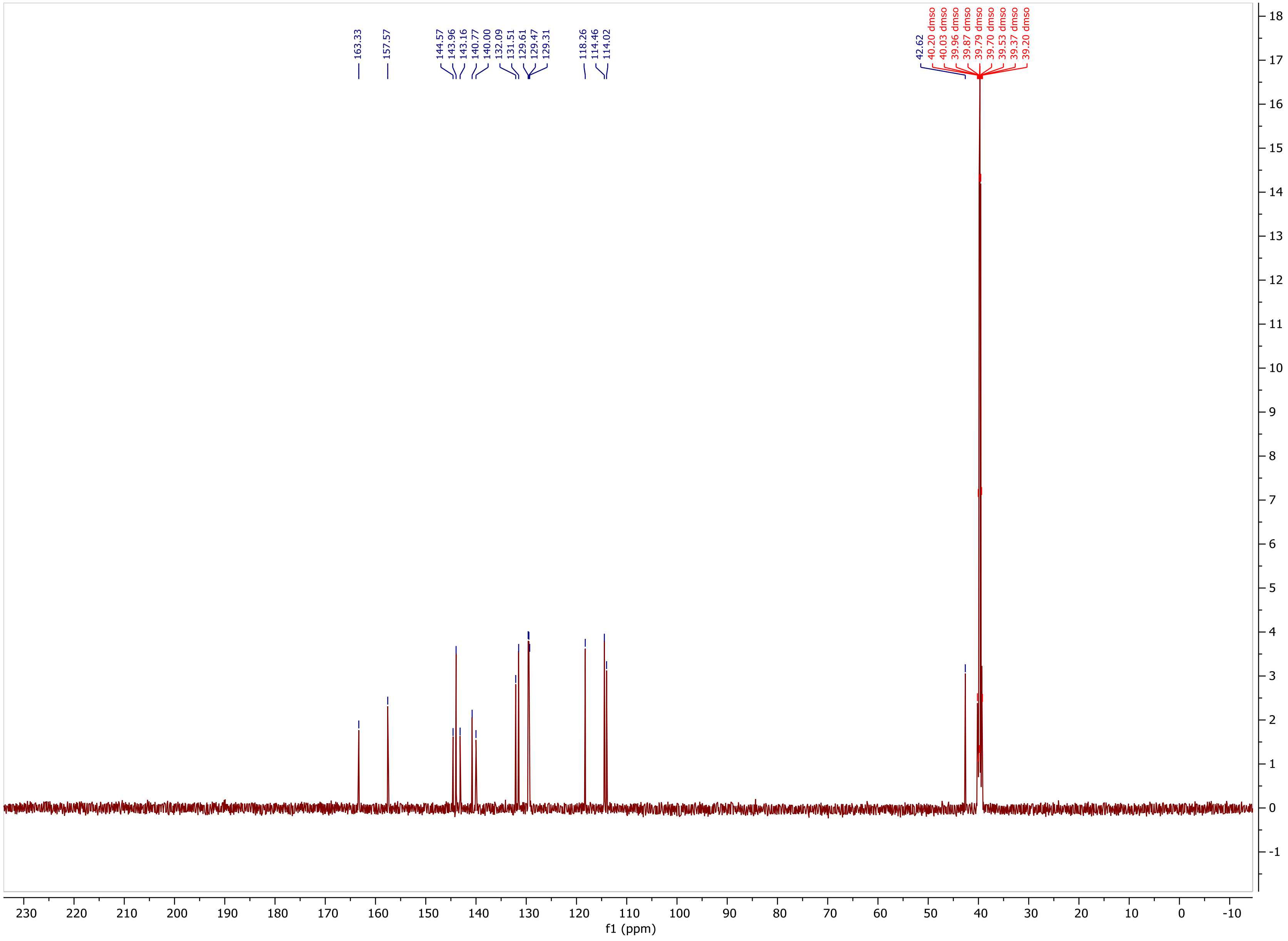
163.33
157.57
144.57
143.96
143.16
140.77
140.00
132.09
131.51
129.61
129.47
129.31
118.26
114.46
114.02
42.62
40.20 dmso
40.03 dmso
39.96 dmso
39.87 dmso
39.79 dmso
39.70 dmso
39.53 dmso
39.37 dmso
39.20 dmso
f1 (ppm)

Compound 17

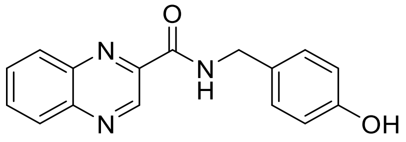

N-(4-hydroxybenzyl)quinoxaline-2-carboxamide. M.p.: 220.1–221.8˚C. Yield: 67%. Light beige powder. ^{1}H-NMR (600 MHz, DMSO-d_6) δ 9.67 (t, J = 6.4 Hz, 1H, amide), 9.54 (s, 1H, pyrazine), 9.47 (s, 1H, hydroxy), 8.22–8.14 (m, 2H, aromatic), 8.06–7.93 (m, 2H, aromatic), 7.53–7.47 (m, 2H, aromatic), 7.36–7.30 (m, 2H, aromatic), 4.61 (d, J = 6.3 Hz, 2H, methylene). ^{13}C-NMR (151 MHz, DMSO-d_6) δ 163.87, 163.05, 149.92, 145.90, 144.91, 144.37, 142.77, 141.30, 140.38, 137.96, 133.55, 132.44, 131.84, 130.70, 129.37, 122.20, 42.58. IR (ATR-Ge, cm-1): 3401 (NH, CONH), 2888 (CH_2), 1676 (CO, CONH), 1528, 1507, 1492 (aromatic). Calculated for $C_{16}H_{13}N_3O_2$ (279.30 g/mol): C, 68.81; H, 4.69%; N, 15.05%. Found: C, 68.91%; H, 4.71%; N, 15.15%. CAS#1223586-16-0.

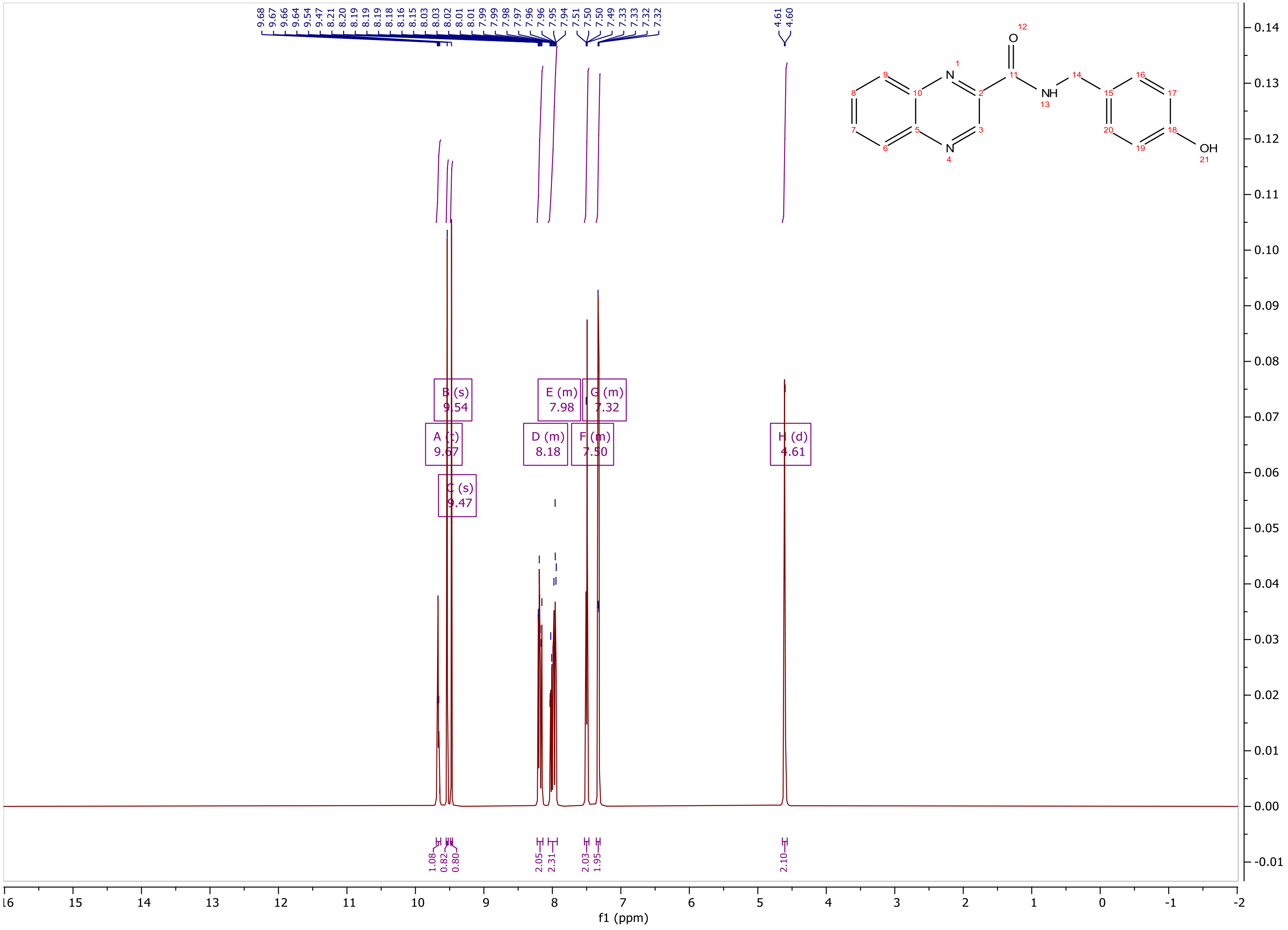

9.68
9.67
9.66
9.64
9.54
9.47
8.21
8.20
8.19
8.19
8.19
8.18
8.16
8.15
8.03
8.03
8.02
8.01
8.01
7.99
7.99
7.98
7.97
7.96
7.96
7.95
7.94
7.51
7.50
7.50
7.49
7.33
7.33
7.32
7.32
4.61
4.60
A (t) 9.67
B (s) 9.54
C (s) 9.47
D (m) 8.18
E (m) 7.98
F (m) 7.50
G (m) 7.32
H (d) 4.61
1.08
0.82
0.80
2.05
2.31
2.03
1.95
2.10
f1 (ppm)
NH
OH

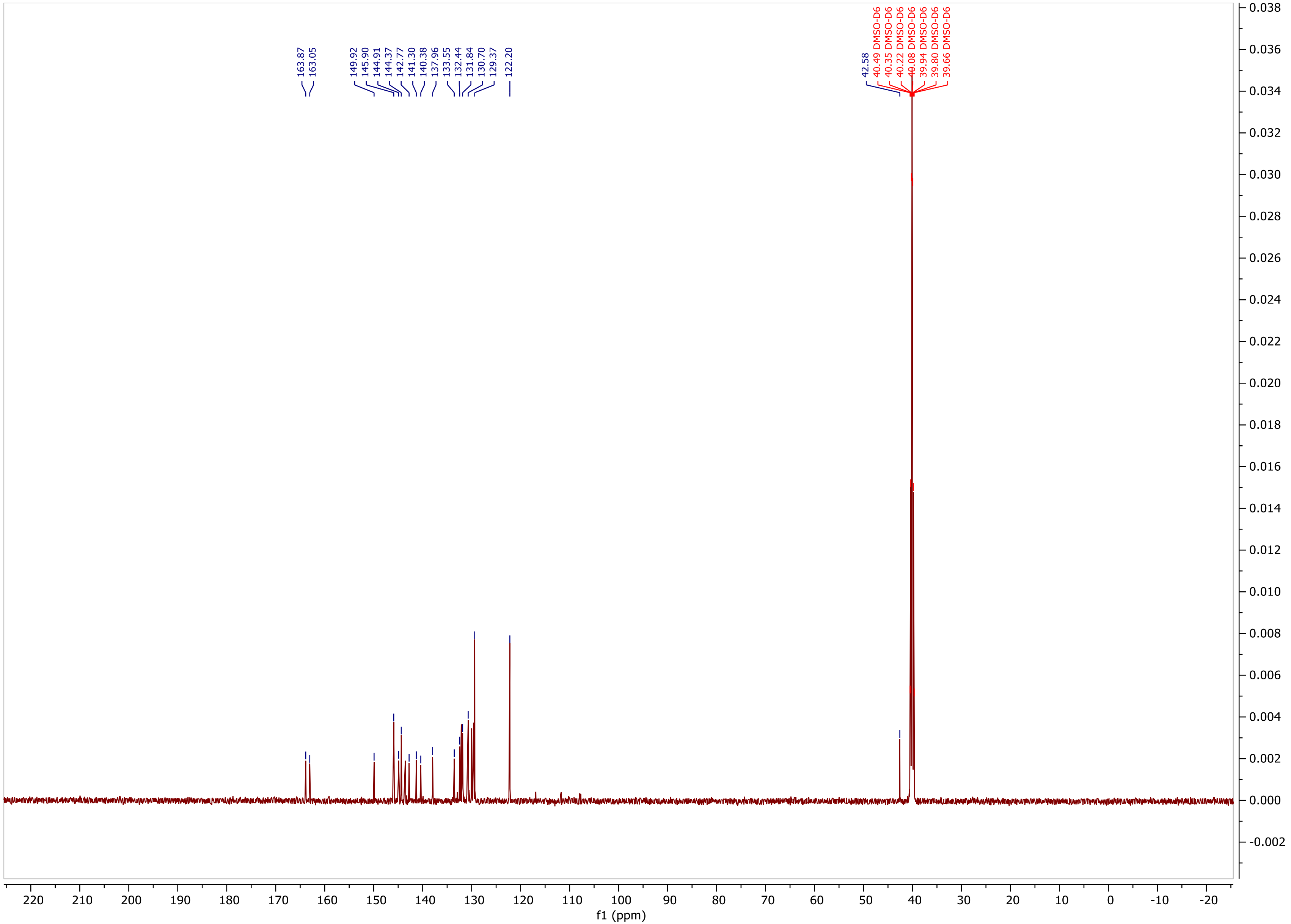
163.87
163.05
149.92
145.90
144.91
144.37
142.77
141.30
140.38
137.96
133.55
132.44
131.84
130.70
129.37
122.20
42.58
40.49 DMSO-D6
40.35 DMSO-D6
40.22 DMSO-D6
40.08 DMSO-D6
39.94 DMSO-D6
39.80 DMSO-D6
39.66 DMSO-D6
f1 (ppm)

Compound 18

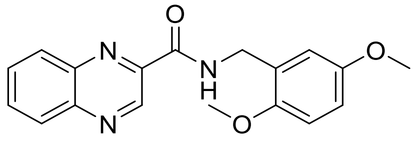

N-(2,5-dimethoxybenzyl)quinoxaline-2-carboxamide. M.p.: 245.5–252.8˚C. Yield: 24%. Beige solid powder. ^{1}H-NMR (500 MHz, DMSO-d_6) δ 9.47 (t, *J* = 6.4 Hz, 1H, amide), 9.15 (s, 1H, pyrazine), 8.22–8.16 (m, 3H, aromatic), 8.01–7.94 (m, 2H, aromatic), 7.19–7.13 (m, 1H, aromatic), 6.60–6.56 (m, 1H, aromatic), 6.50–6.44 (m, 1H, aromatic), 4.49 (d, *J* = 6.2 Hz, 2H, methylene), 3.84 (s, 3H, methoxy), 3.73 (s, 3H, methoxy). ^{13}C-NMR (126 MHz, DMSO-d_6) δ 163.13, 159.96, 157.87, 144.48, 143.85, 143.15, 139.93, 132.02, 131.42, 129.63, 129.24, 128.84, 118.59, 104.57, 98.46, 98.40, 55.66, 55.36, 37.66. IR (ATR-Ge, cm^{-1}): 3303 (NH, CONH), 2909 (CH_2), 1662 (CO, CONH), 1607, 1571, 1537 (aromatic). Calculated for $C_{18}H_{17}N_3O_3$ (323.35 g/mol): C, 66.86%; H, 5.30%; N, 13.00%. Found: C, 66.63%; H, 5.28%; N, 12.92%. CAS# 2183660-19-5.

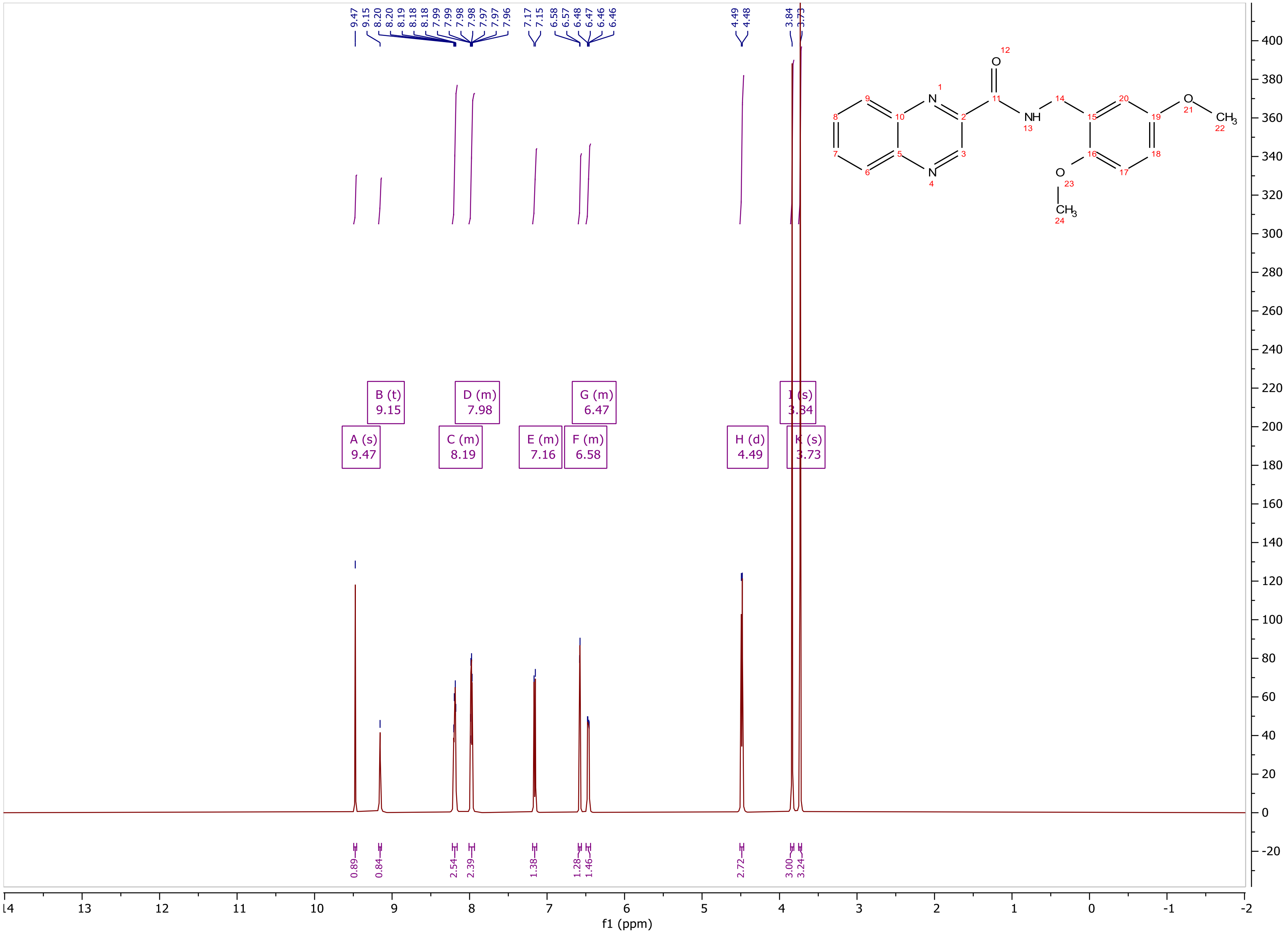

A (s)
9.47
B (t)
9.15
C (m)
8.19
D (m)
7.98
E (m)
7.16
F (m)
6.58
G (m)
6.47
H (d)
4.49
I (s)
3.84
K (s)
3.73
f1 (ppm)

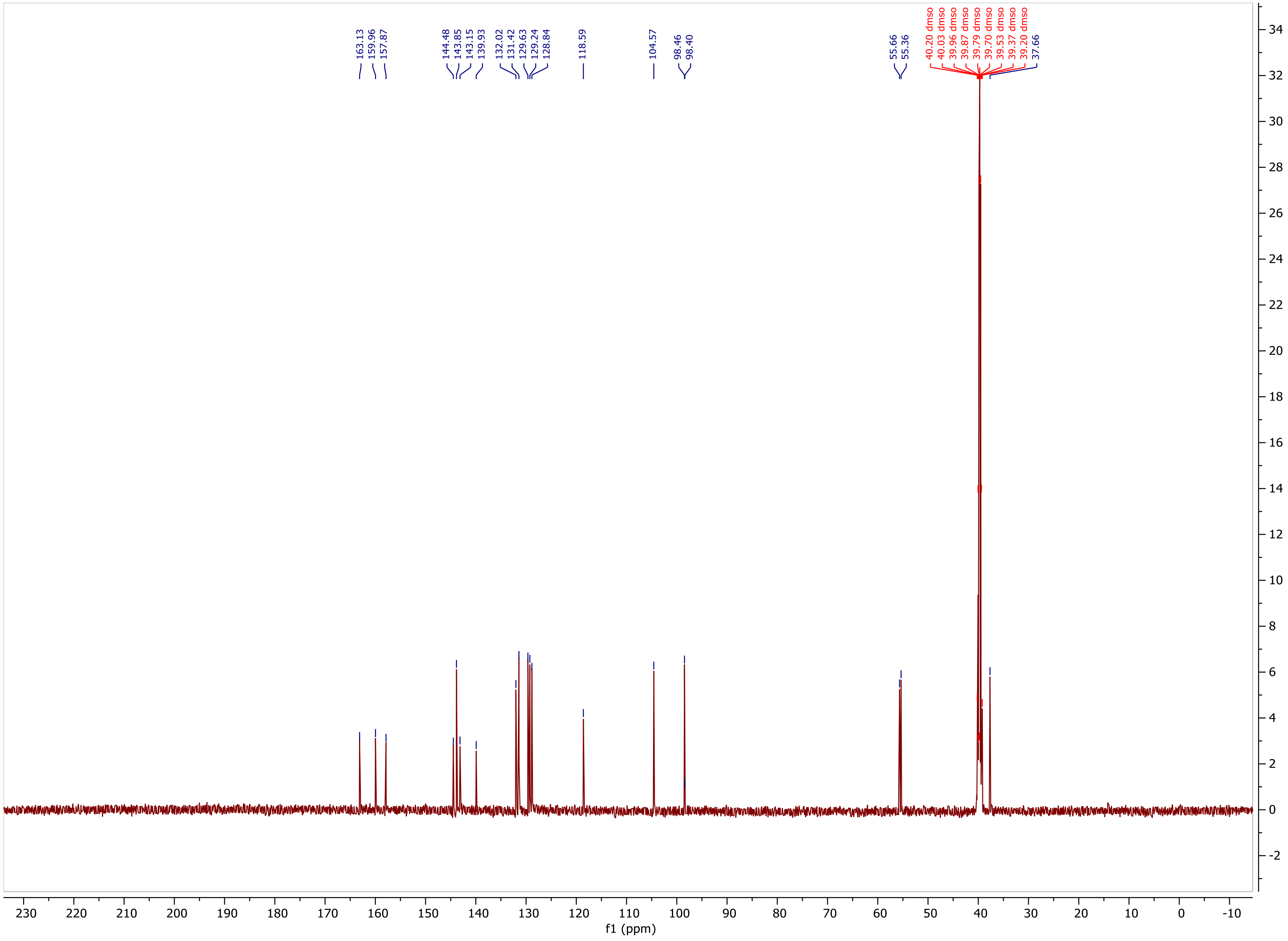

163.13
159.96
157.87
144.48
143.85
143.15
139.93
132.02
131.42
129.63
129.24
128.84
118.59
104.57
98.46
98.40
55.66
55.36
40.20 dmso
40.03 dmso
39.96 dmso
39.87 dmso
39.79 dmso
39.70 dmso
39.53 dmso
39.37 dmso
39.20 dmso
37.66
f1 (ppm)

Compound 19

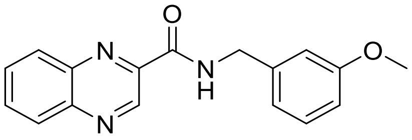

N-(3-methoxybenzyl)quinoxaline-2-carboxamide. M.p.: 220.0–221.8˚C. Yield: 49%. Beige solid powder. ^{1}H-NMR (600 MHz, DMSO-d_6) δ 9.62 (t, J = 6.0 Hz, 1H, amide), 9.45 (s, 1H, pryazine), 8.21–8.15 (m, 2H, aromatic), 8.00–7.93 (m, 2H, aromatic), 7.61–7.57 (m, 1H, aromatic), 7.42–7.33 (m, 3H, aromatic), 4.58 (d, J = 6.5, 2H, methylene), 3.29 (s, 3H, methoxy). ^{13}C-NMR (151 MHz, DMSO-$_{d6}$163.75, 159.83, 144.93, 144.34, 143.52, 141.31, 140.35, 132.42, 131.82, 129.97, 129.92, 129.66, 120.20, 113.84, 112.72, 55.52, 43.01. IR (ATR-Ge, cm^{-1}): 3203 (NH, CONH), 1678 (CH_2), 2920 (CO, CONH), 1620, 1595, 1551 (aromatic). Calculated for $C_{17}H_{15}N_3O_2$ (293.33 g/mol): C, 69.61%; H, 5.15%; N, 14.33%. Found: C, 69.44%; H, 5.12%; N, 14.01%. CAS# 2183616-53-5.

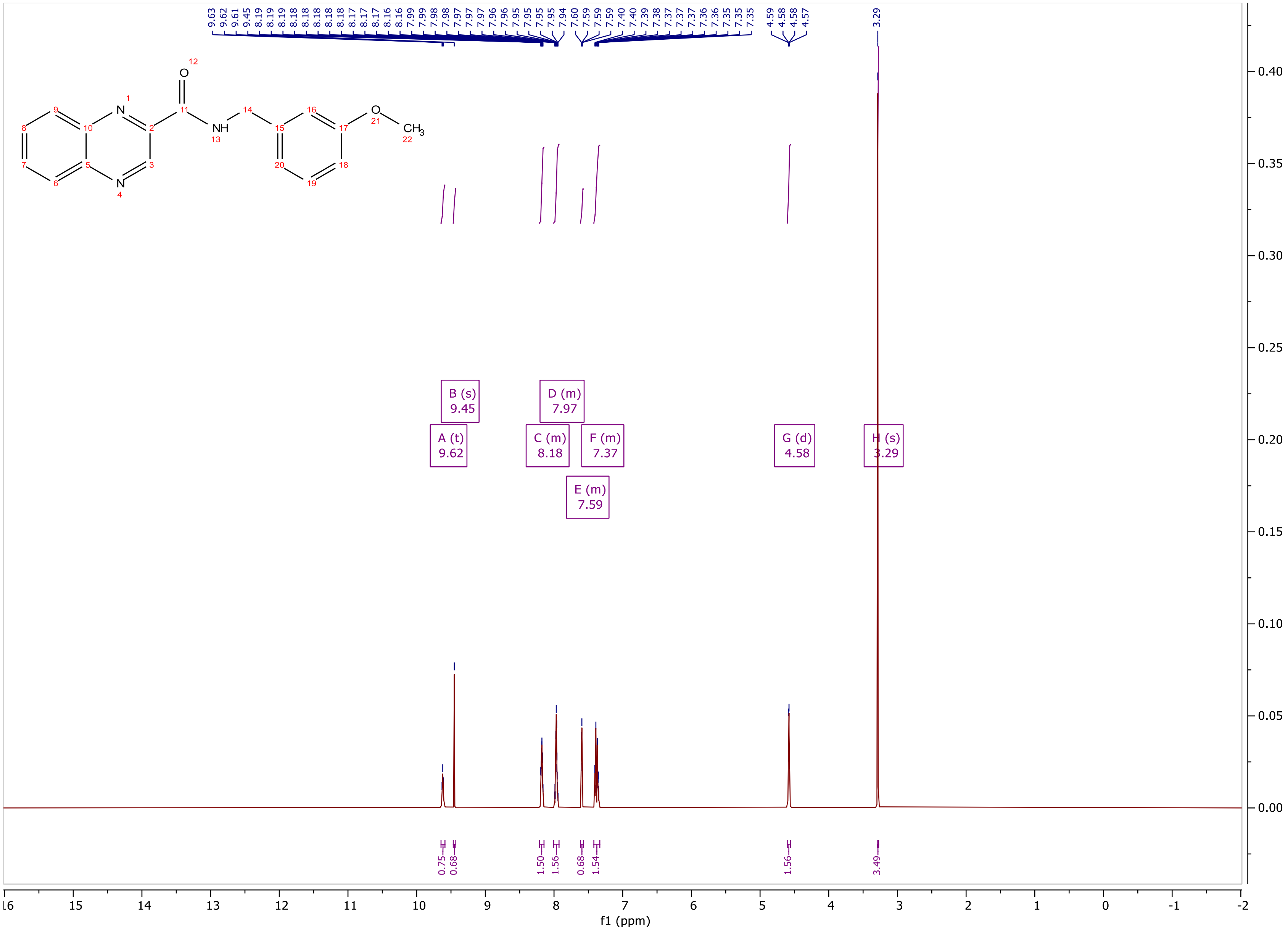

9.63 9.62 9.61 9.45 8.19 8.19 8.19 8.18 8.18 8.18 8.18 8.17 8.17 8.17 8.16 8.16 7.99 7.99 7.98 7.98 7.97 7.97 7.97 7.96 7.96 7.95 7.95 7.95 7.95 7.94 7.60 7.59 7.59 7.59 7.40 7.39 7.38 7.37 7.37 7.37 7.36 7.36 7.35 7.35 7.35 4.59 4.58 4.58 4.57 3.29
B (s) 9.45
D (m) 7.97
A (t) 9.62
C (m) 8.18
F (m) 7.37
E (m) 7.59
G (d) 4.58
H (s) 3.29
0.75 0.68 1.50 1.56 0.68 1.54 1.56 3.49
f1 (ppm)
NH
O
N
CH3

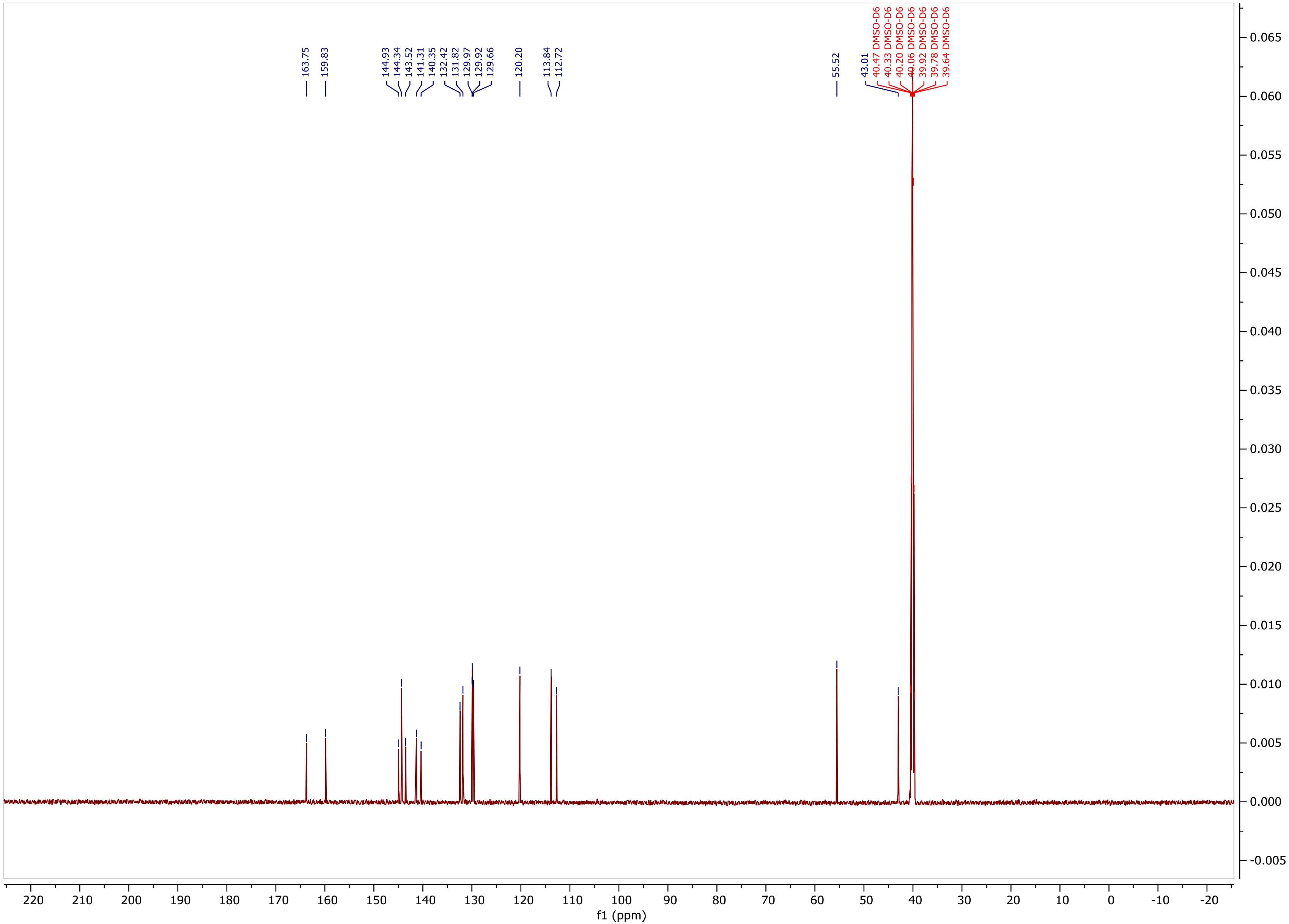
163.75
159.83
144.93
144.34
143.52
141.31
140.35
132.42
131.82
129.97
129.92
129.66
120.20
113.84
112.72
55.52
43.01
40.47 DMSO-D6
40.33 DMSO-D6
40.20 DMSO-D6
40.06 DMSO-D6
39.92 DMSO-D6
39.78 DMSO-D6
39.64 DMSO-D6
f1 (ppm)

Compound 20

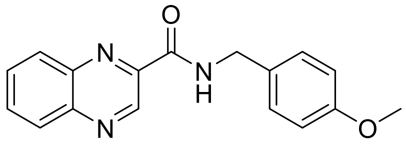

_N_-(4-methoxybenzyl)quinoxaline-2-carboxamide. M.p.: 222.4–222.8˚C. Yield: 58%. Light yellow solid powder. ^{1}H-NMR (500 MHz, DMSO-d_6) δ 9.50 (t, *J* = 6.4 Hz, 1H, amide), 9.48 (s, 1H, pyrazine), 8.22–8.15 (m, 2H, aromatic), 8.00–7.94 (m, 2H, aromatic), 7.34–7.29 (m, 2H, aromatic), 6.91–6.84 (m, 2H, aromatic), 4.50 (d, *J* = 6.3 Hz, 2H, methylene), 3.72 (s, 3H, methoxy). ^{13}C-NMR (126 MHz, DMSO-d_6) δ 163.19, 158.47, 144.60, 143.92, 143.12, 139.96, 132.00, 131.42, 131.33, 129.56, 129.26, 129.12, 129.00, 113.86, 55.21, 42.14. IR (ATR-Ge, cm^{-1}): 3303 (NH, CONH), 1662 (CH_2), 2909 (CO, CONH), 1614, 1585, 1571 (aromatic). Calculated for $C_{17}H_{15}N_3O_2$ (293.33 g/mol): C, 69.61%; H, 5.15%; N, 14.33%. Found: C, 69.29%; H, 5.14%; N, 13.98%. CAS# 287945-56-6.

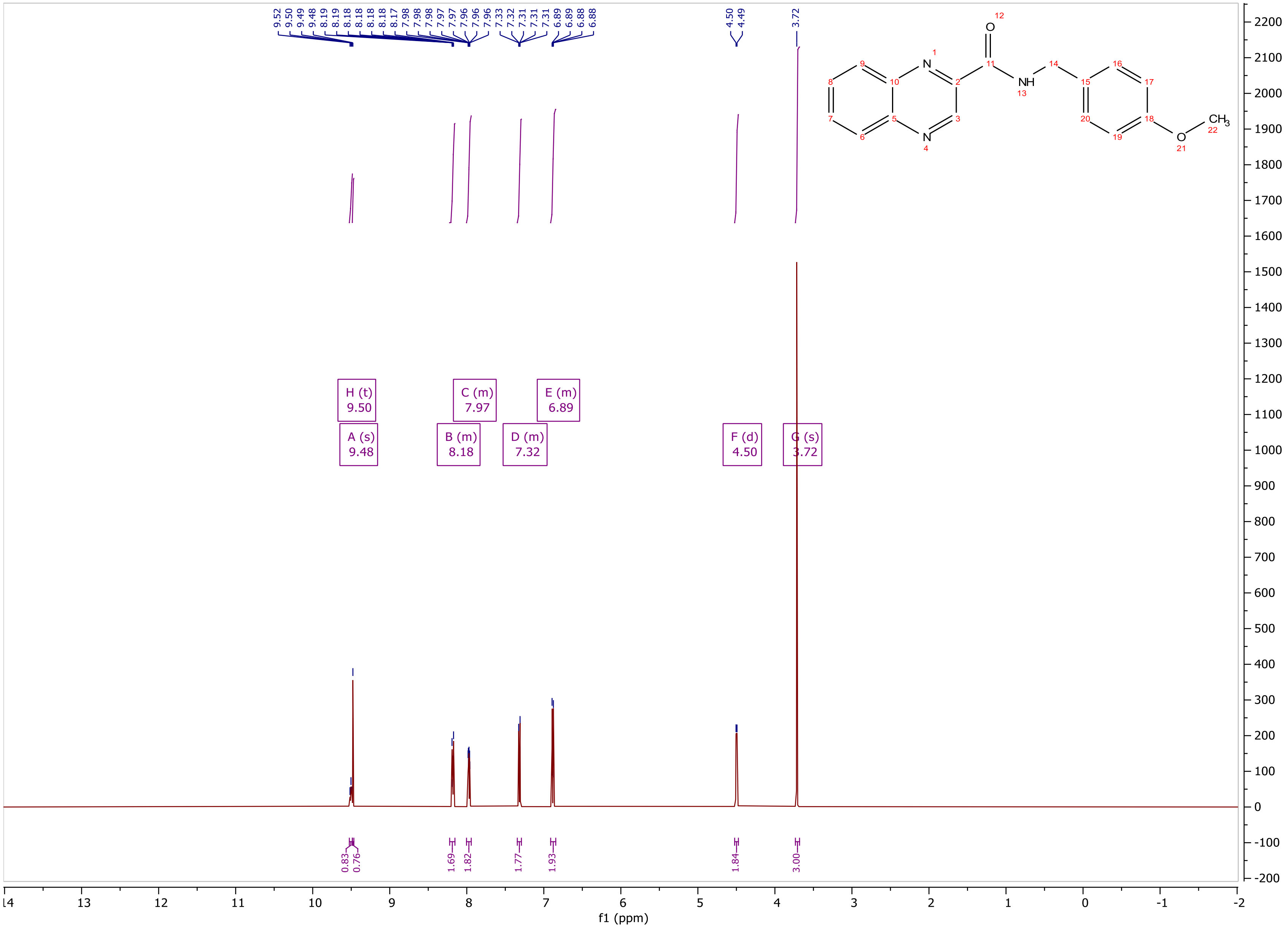

9.52 9.50 9.49 9.48 8.19 8.19 8.18 8.18 8.18 8.18 8.17 7.98 7.98 7.98 7.97 7.97 7.96 7.96 7.96 7.33 7.32 7.31 7.31 7.31 6.89 6.89 6.88 6.88
4.50 4.49
3.72
H (t) 9.50
A (s) 9.48
B (m) 8.18
C (m) 7.97
D (m) 7.32
E (m) 6.89
F (d) 4.50
G (s) 3.72
0.83 0.76 1.69 1.82 1.77 1.93 1.84 3.00
f1 (ppm)

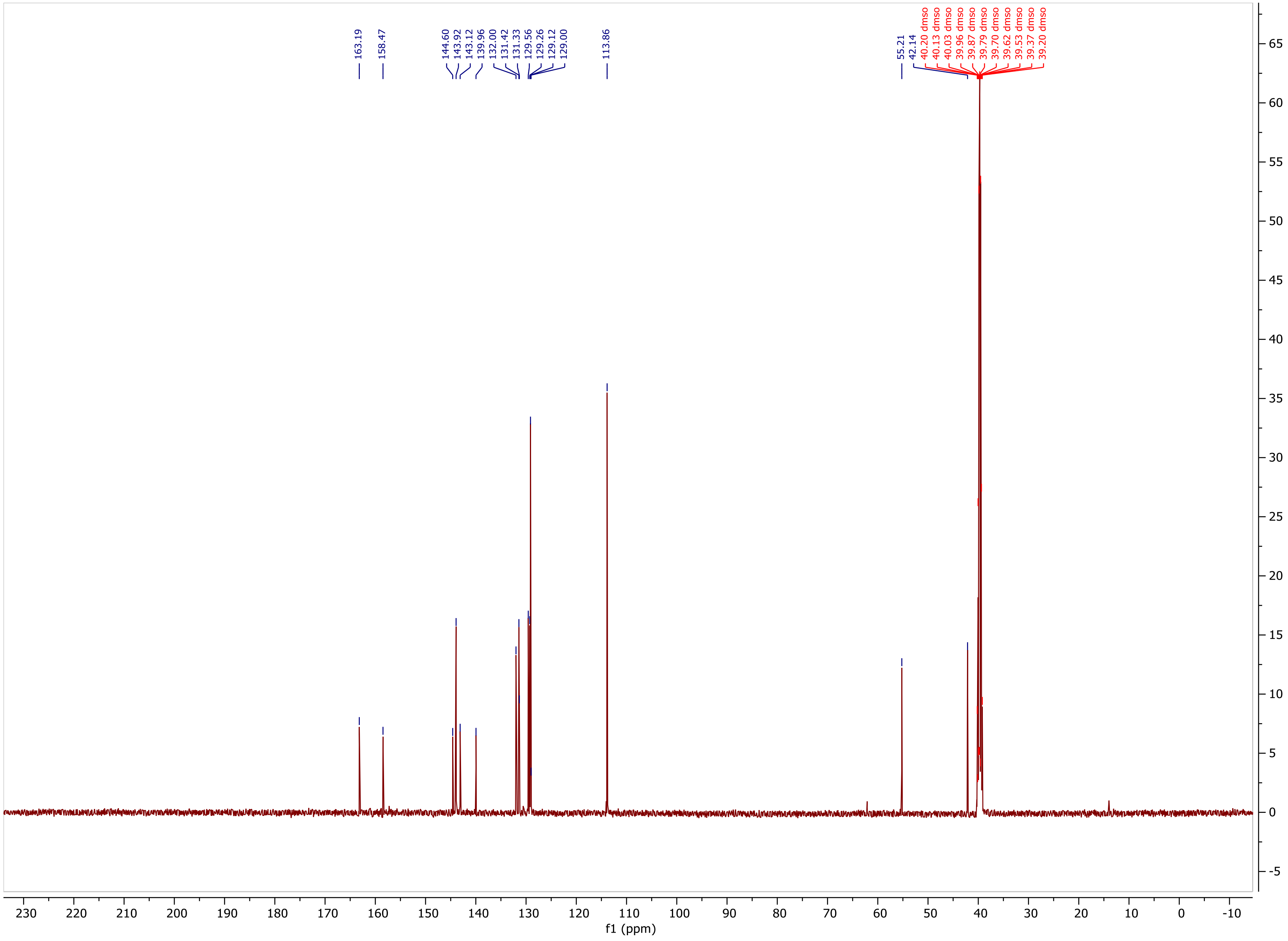

163.19
158.47
144.60
143.92
143.12
139.96
132.00
131.42
131.33
129.56
129.26
129.12
129.00
113.86
55.21
42.14
40.20 dmso
40.13 dmso
40.03 dmso
39.96 dmso
39.87 dmso
39.79 dmso
39.70 dmso
39.62 dmso
39.53 dmso
39.37 dmso
39.20 dmso
f1 (ppm)

Compound 21

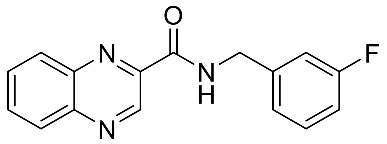

N-(3-fluorobenzyl)quinoxaline-2-carboxamide. M.p.: 201.3–205.1˚C. Yield: 51%. White powder. ^{1}H-NMR (600 MHz, DMSO-d_6) δ 9.63 (t, J = 6.4 Hz, 1H, amide), 9.45 (s, 1H, pyrazine), 8.19–8.12 (m, 2H, aromatic), 7.99–7.91 (m, 2H, aromatic), 7.37–7.30 (m, 1H, aromatic), 7.21–7.14 (m, 2H, aromatic), 7.06–7.00 (m, 1H, aromatic), 4.55 (d, J = 6.4 Hz, 2H, methylene). ^{13}C-NMR (151 MHz, DMSO-d_6) δ 163.91, 163.56, 161.95, 144.83, 144.35, 143.54, 142.77, 140.36, 132.43, 131.82, 129.96, 129.66, 124.01, 114.77, 114.63, 114.10, 42.61. IR (ATR-Ge, cm^{-1}): 3308 (NH, CONH), 2958 (CH_2), 1719 (CO, CONH), 1655, 1615, 1590 (aromatic). Calculated for $C_{16}H_{12}FN_3O$ (281.29 g/mol): C, 68.32%; H, 4.30%; N, 14.94%. CAS#: 1387973-47-8.

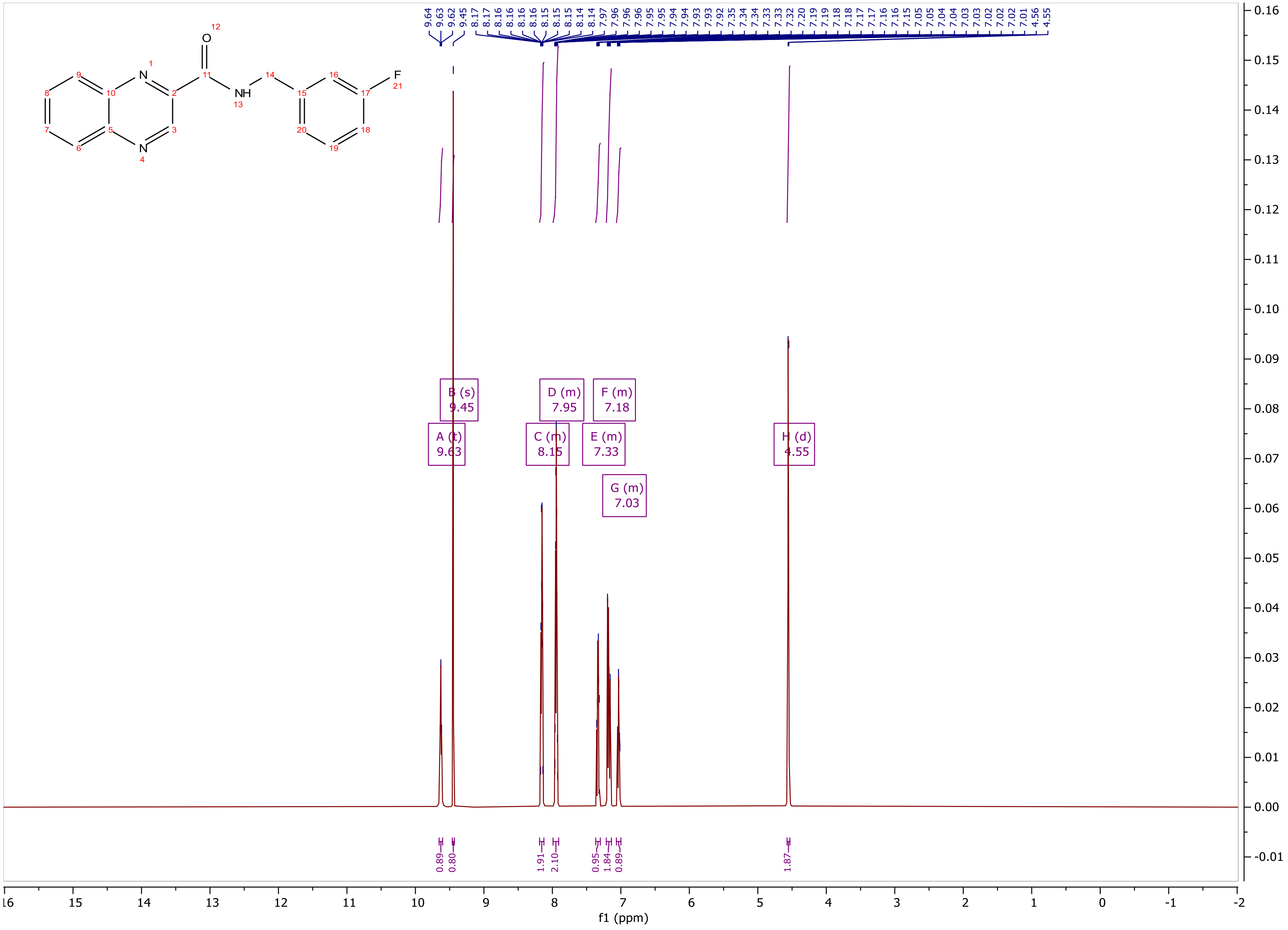

A (t)
9.63
B (s)
9.45
C (m)
8.15
D (m)
7.95
E (m)
7.33
F (m)
7.18
G (m)
7.03
H (d)
4.55
0.89
0.80
1.91
2.10
0.95
1.84
0.89
1.87
f1 (ppm)

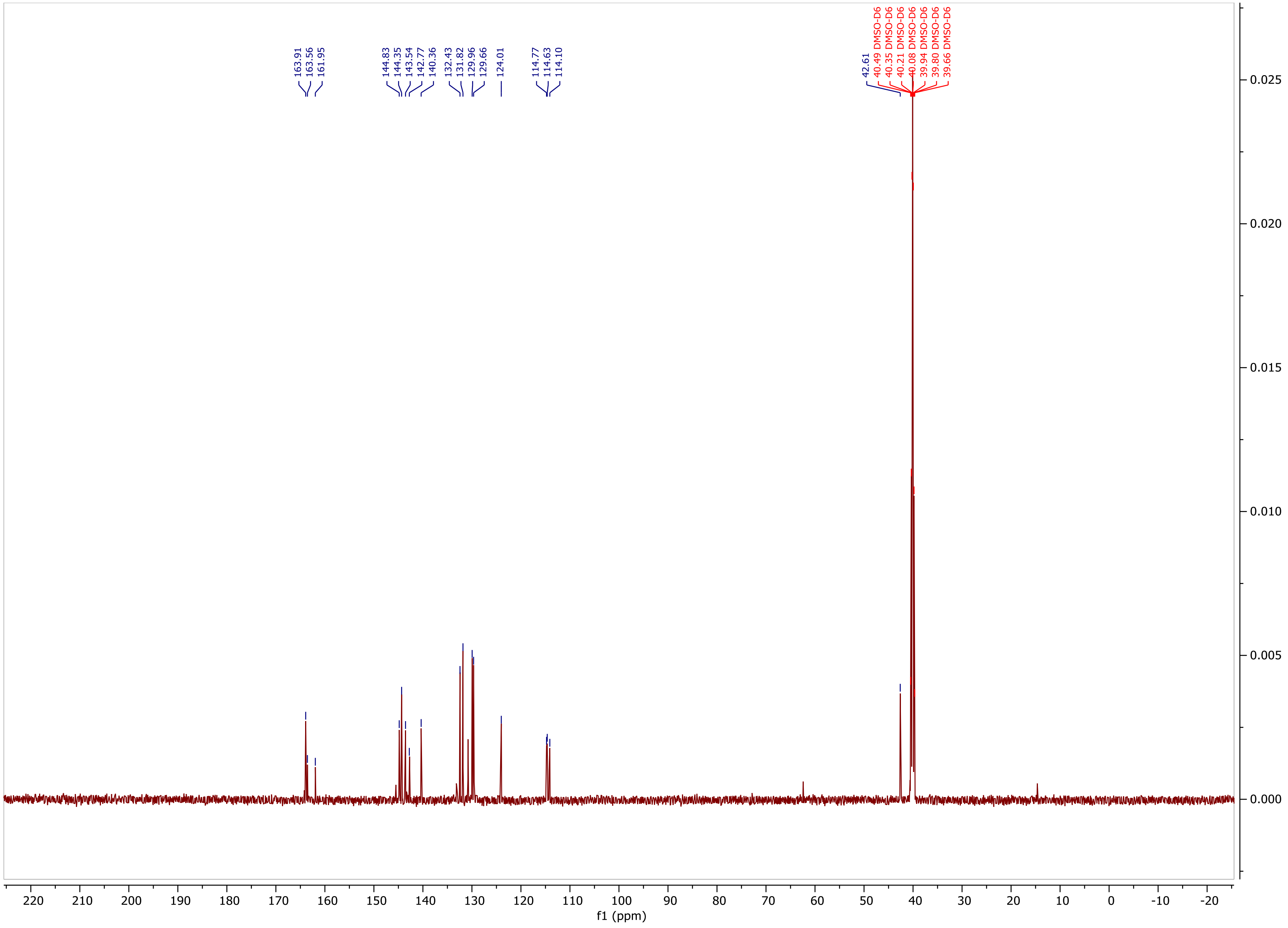

163.91
163.56
161.95
144.83
144.35
143.54
142.77
140.36
132.43
131.82
129.96
129.66
124.01
114.77
114.63
114.10
42.61
40.49 DMSO-D6
40.35 DMSO-D6
40.21 DMSO-D6
40.08 DMSO-D6
39.94 DMSO-D6
39.80 DMSO-D6
39.66 DMSO-D6
f1 (ppm)

Compound 22

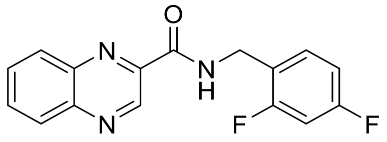

N-(2,4-difluorobenzyl)quinoxaline-2-carboxamide. M.p.: 199.4–201.2˚C. Yield: 51%. White powder. ^{1}H-NMR (600 MHz, DMSO-d_6) δ 9.59 (t, J = 6.4 Hz, 1H, amide), 9.44 (s, 1H, pyrazine), 8.19–8.12 (m, 2H, aromatic), 7.99–7.89 (m, 2H, aromatic), 7.40–7.38 (m, 1H, aromatic), 7.14–7.06 (m, 2H, aromatic), 4.52 (d, J = 6.4 Hz, 2H, methylene). ^{13}C-NMR (151 MHz, DMSO-d_6) δ 163.77, 162.59, 160.99, 144.88, 144.32, 143.52, 140.35, 135.97, 132.42, 131.83, 130.12, 129.94, 129.66, 115.62, 115.47, 42.38. IR (ATR-Ge, cm^{-1}): 3308 (NH, CONH), 2958 (CH_2), 1719 (CO, CONH), 1655, 1615, 1590 (aromatic). Calculated for $C_{16}H_{12}FN_3O$ (281.29 g/mol): C, 68.32%; H, 4.30%; N, 14.94%. CAS#: 1387973-47-8.

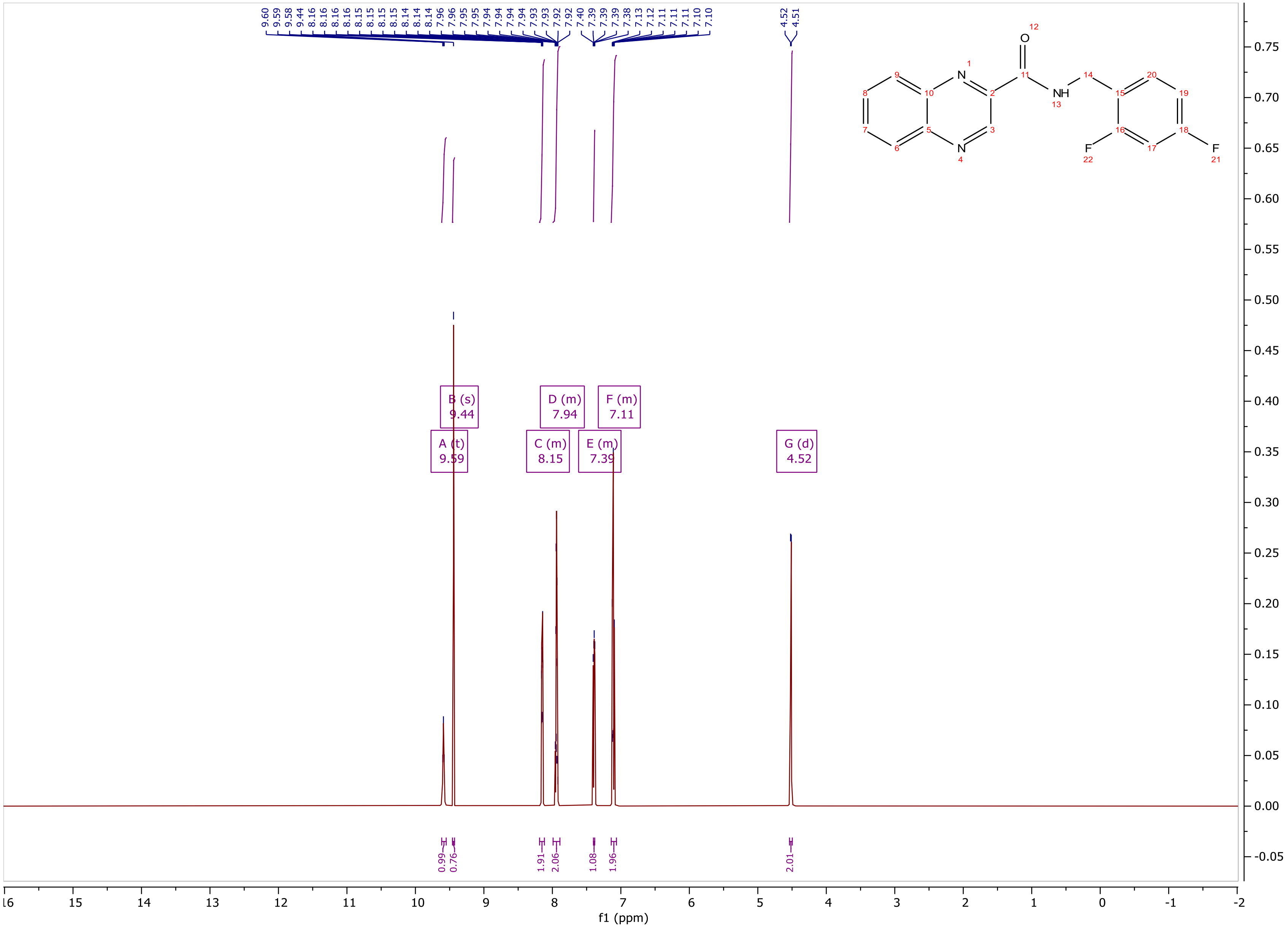

9.60
9.59
9.58
9.44
8.16
8.16
8.16
8.15
8.15
8.15
8.14
8.14
8.14
7.96
7.96
7.95
7.95
7.94
7.94
7.94
7.94
7.93
7.93
7.92
7.92
7.40
7.39
7.39
7.39
7.39
7.38
7.13
7.12
7.11
7.11
7.11
7.10
7.10
4.52
4.51
A (t) 9.59
B (s) 9.44
C (m) 8.15
D (m) 7.94
E (m) 7.39
F (m) 7.11
G (d) 4.52
0.99
0.76
1.91
2.06
1.08
1.96
2.01
f1 (ppm)
N
NH
O
F
F

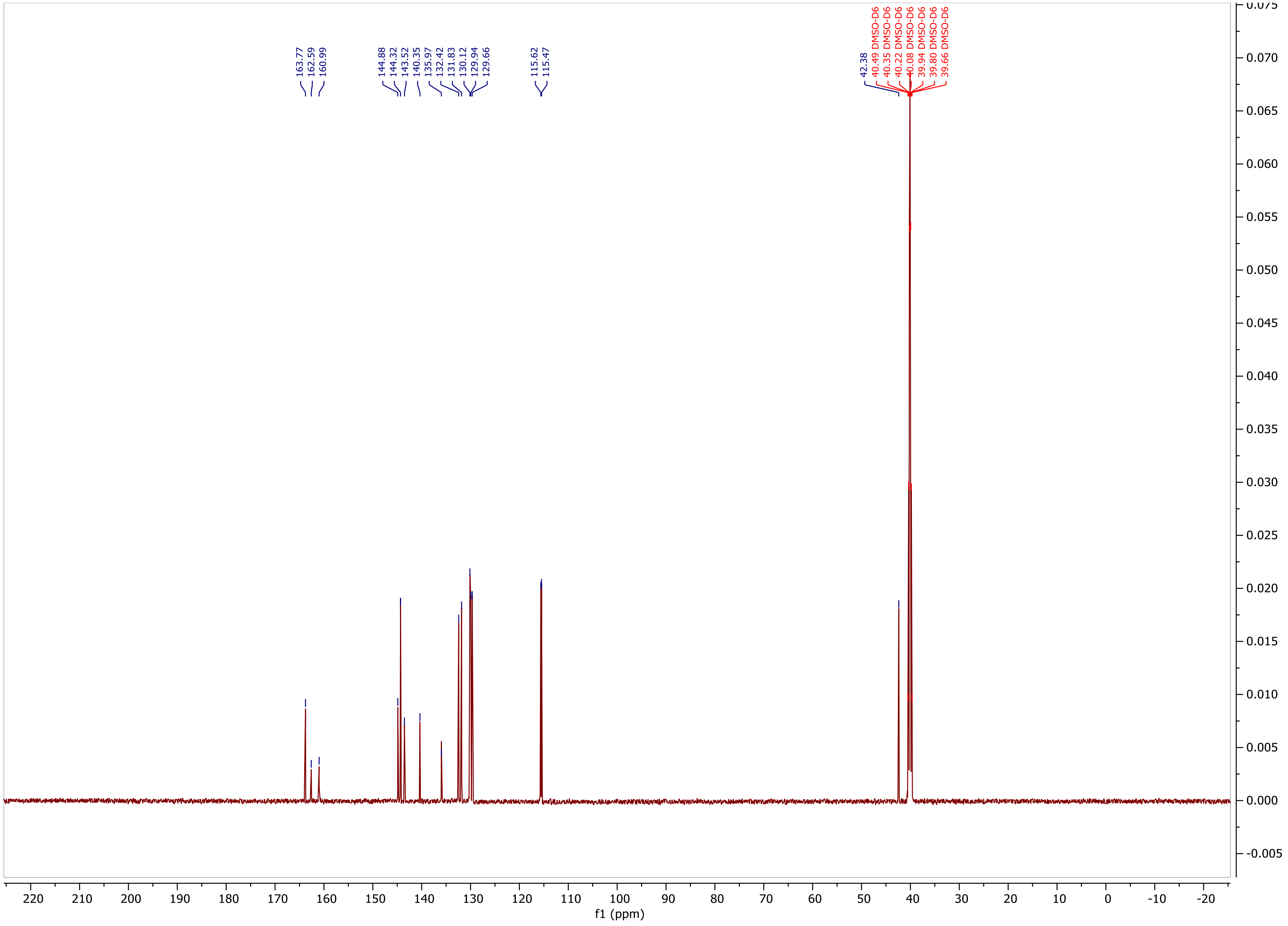

163.77
162.59
160.99
144.88
144.32
143.52
140.35
135.97
132.42
131.83
130.12
129.94
129.66
115.62
115.47
42.38
40.49 DMSO-D6
40.35 DMSO-D6
40.22 DMSO-D6
40.08 DMSO-D6
39.94 DMSO-D6
39.80 DMSO-D6
39.66 DMSO-D6
f1 (ppm)

Compound 23

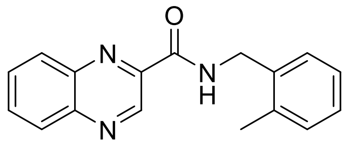

_N_-(2-methylbenzyl)quinoxaline-2-carboxamide. M.p.: 212.2–214.2˚C. Yield: 34%. Pale yellow powder. ^{1}H-NMR (500 MHz, DMSO-d_6) δ 9.49 (s, 1H, pyrazine), 9.43 (t, J = 6.2 Hz, 1H, amide), 8.24–8.15 (m, 2H, aromatic), 8.03–7.93 (m, 2H, aromatic), 7.34–7.28 (m, 1H, aromatic), 7.21–7.10 (m, 3H, aromatic), 4.56 (d, J = 6.2 Hz, 2H, methylene), 2.36 (s, 3H, methyl). ^{13}C-NMR (126 MHz, DMSO-d_6) δ 163.34, 144.56, 143.94, 143.14, 139.98, 136.91, 135.63, 132.02, 131.42, 130.07, 129.60, 129.26, 127.67, 127.03, 125.93, 40.58, 18.95. IR (ATR-Ge, cm^{-1}): 3314 (NH, CONH), 2916 (CH_2), 1656 (CO, CONH), 1627, 1591, 1576 (aromatic). Calculated for $C_{17}H_{15}N_3O$ (277.33 g/mol): C, 73.63%; H, 5.45%; N, 15.15%. Found: C, 73.38%; H, 5.23%; N, 14.95%. CAS# 930513-22-7.

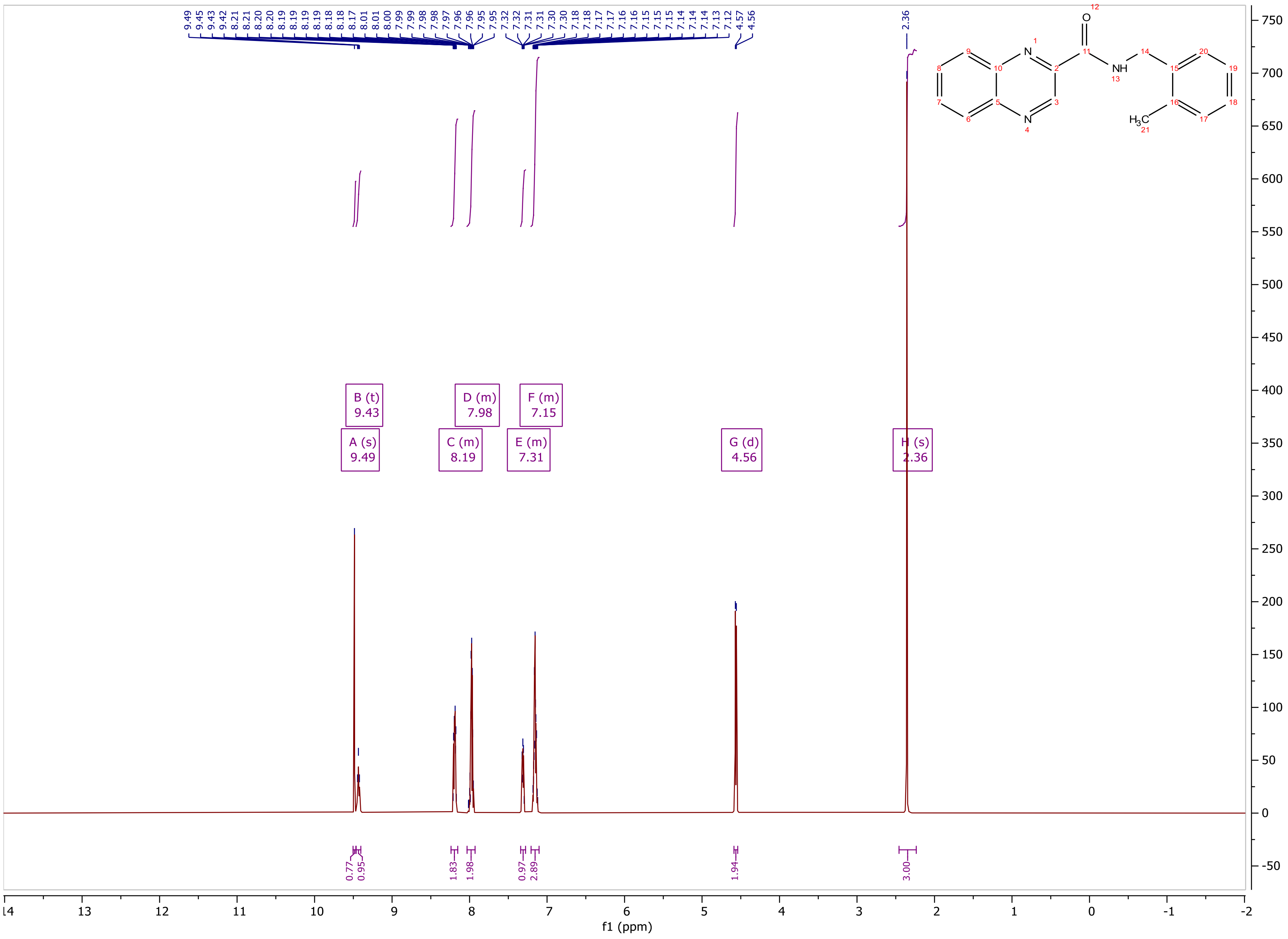

9.49 9.45 9.43 9.42 8.21 8.21 8.20 8.20 8.19 8.19 8.19 8.19 8.18 8.18 8.17 8.01 8.01 8.00 7.99 7.99 7.98 7.98 7.97 7.96 7.96 7.95 7.95 7.32 7.32 7.31 7.31 7.30 7.30 7.18 7.17 7.17 7.16 7.15 7.15 7.15 7.14 7.14 7.13 7.12 4.57 4.56
2.36
A (s) 9.49
B (t) 9.43
C (m) 8.19
D (m) 7.98
E (m) 7.31
F (m) 7.15
G (d) 4.56
H (s) 2.36
0.77 0.95 1.83 1.98 0.97 2.89 1.94 3.00
f1 (ppm)
NH
H_3C

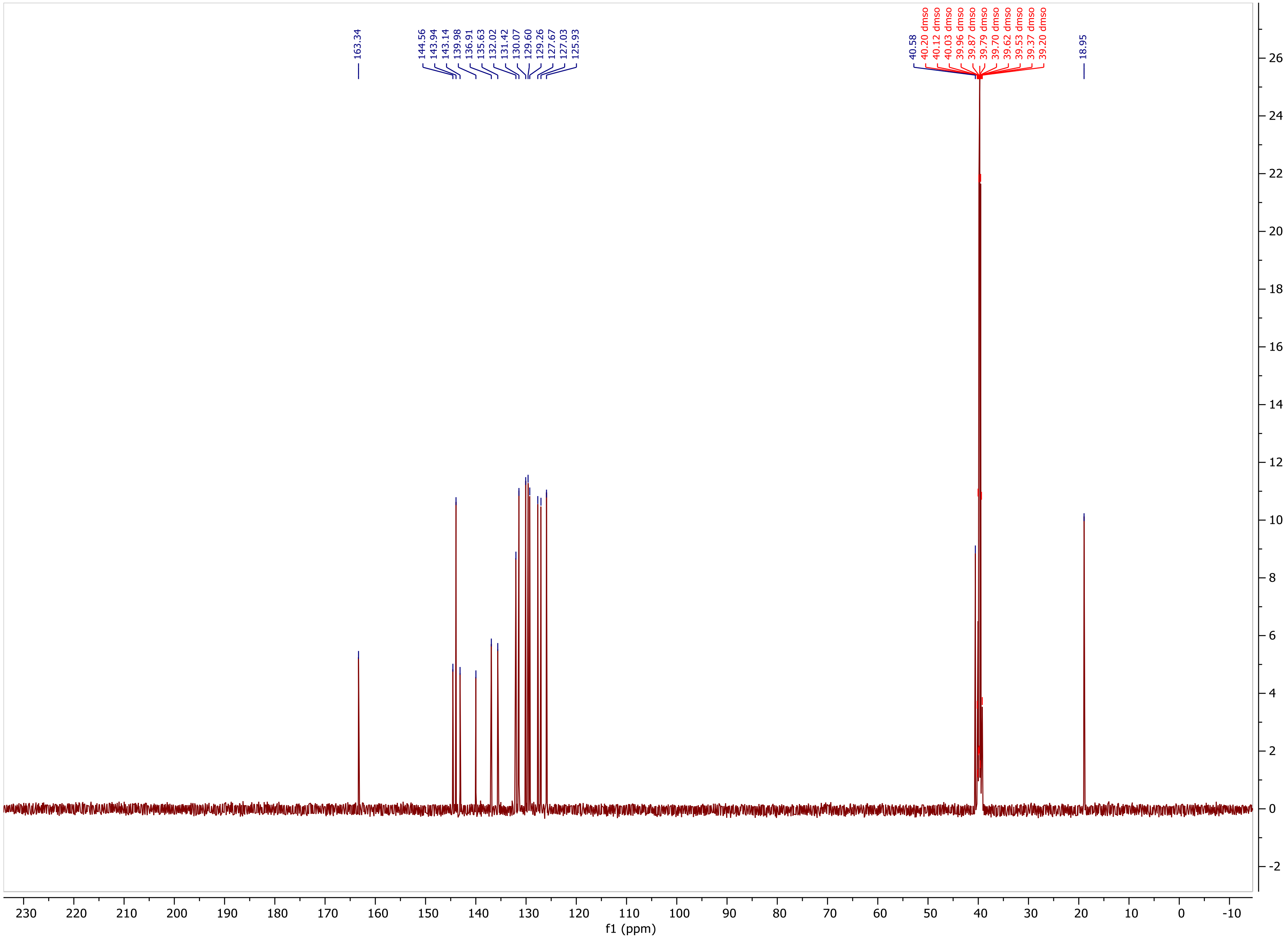

163.34
144.56
143.94
143.14
139.98
136.91
135.63
132.02
131.42
130.07
129.60
129.26
127.67
127.03
125.93
40.58
40.20 dmso
40.12 dmso
40.03 dmso
39.96 dmso
39.87 dmso
39.79 dmso
39.70 dmso
39.62 dmso
39.53 dmso
39.37 dmso
39.20 dmso
18.95
f1 (ppm)

Compound 24

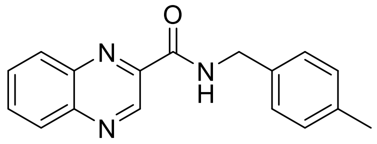

N-(4-methylbenzyl)quinoxaline-2-carboxamide. M.p.: 205.4–207.2˚C. Yield: 56%. White powder. ^{1}H-NMR (500 MHz, DMSO-d_6) δ 9.50 (s, 1H, pyrazine), 9.41 (t, J = 6.2 Hz, 1H, amide), 8.21 (m, 2H, aromatic), 8.03–7.93 (m, 2H, aromatic), 7.36–7.28 (m, 2H, aromatic), 7.21–7.10 (m, 2H, aromatic), 4.57 (d, J = 6.2 Hz, 2H, methylene), 2.37 (s, 3H, methyl).^{13}C-NMR (126 MHz, Chloroform-d) δ 163.11, 143.94, 143.90, 143.39, 140.21, 137.42, 134.77, 131.50, 130.74, 129.56, 129.49, 129.44, 127.96, 43.35, 21.08. IR (ATR-Ge, cm^{-1}): 3348 (NH, CONH), 2926 (CH_2), 1671 (CO, CONH), 1574, 1533, 1494 (aromatic). Calculated for $C_{17}H_{15}N_3O$ (277.33 g/mol): C, 73.63%; H, 5.45%; N, 15.15%. Found: C, 73.01%; H, 5.35%; N, 14.83%. CAS# 878922-59-9.

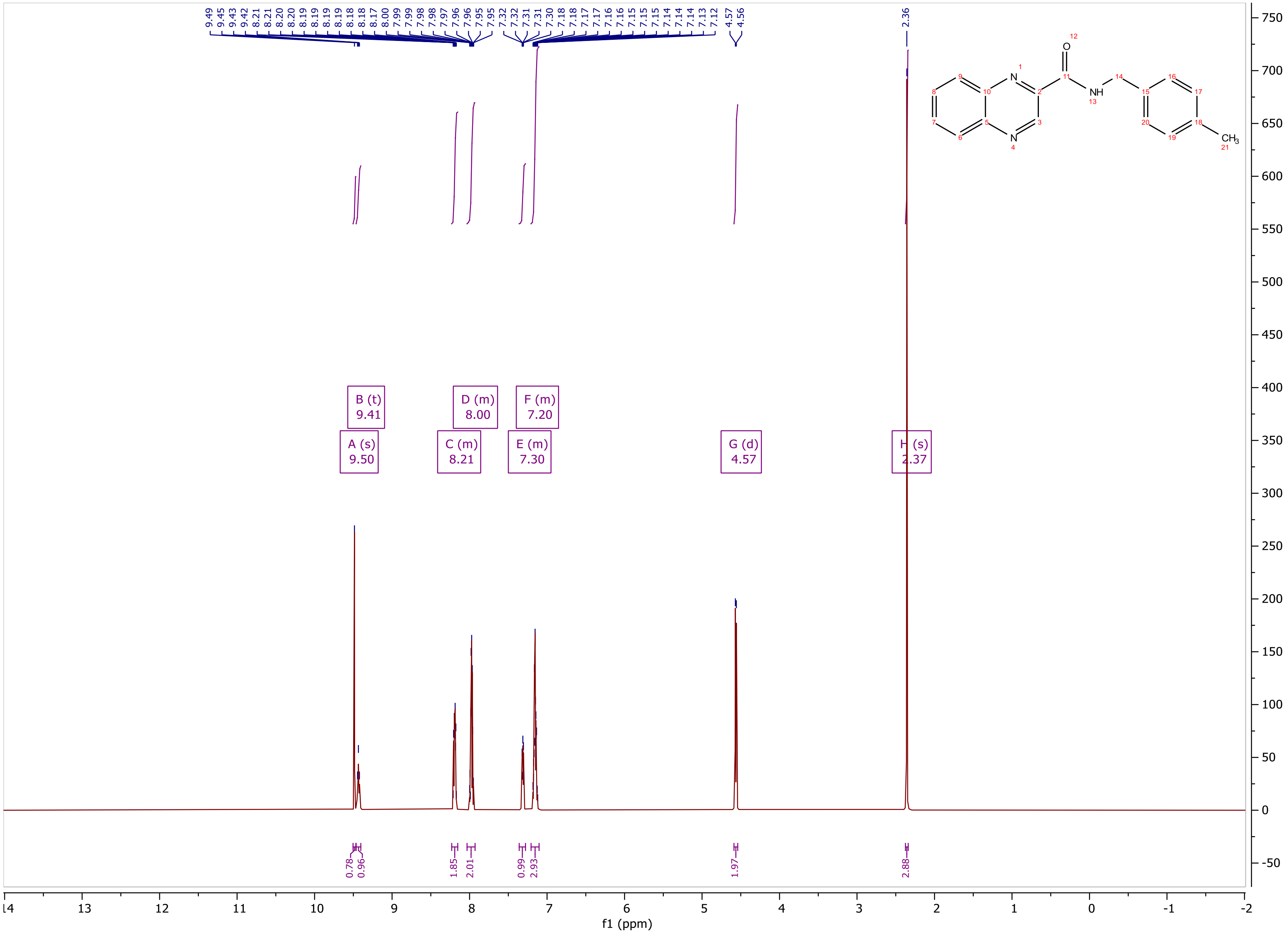
9.49 9.45 9.43 9.42 8.21 8.21 8.20 8.20 8.19 8.19 8.19 8.19 8.18 8.18 8.17 8.00 7.99 7.99 7.98 7.98 7.97 7.96 7.96 7.95 7.95 7.32 7.32 7.31 7.31 7.30 7.18 7.18 7.17 7.17 7.16 7.15 7.15 7.15 7.14 7.14 7.14 7.13 7.12 4.57 4.56
2.36
B (t) 9.41
D (m) 8.00
F (m) 7.20
A (s) 9.50
C (m) 8.21
E (m) 7.30
G (d) 4.57
H (s) 2.37
0.78 0.96 1.85 2.01 0.99 2.93 1.97 2.88
f1 (ppm)
O
N
NH
N
CH3

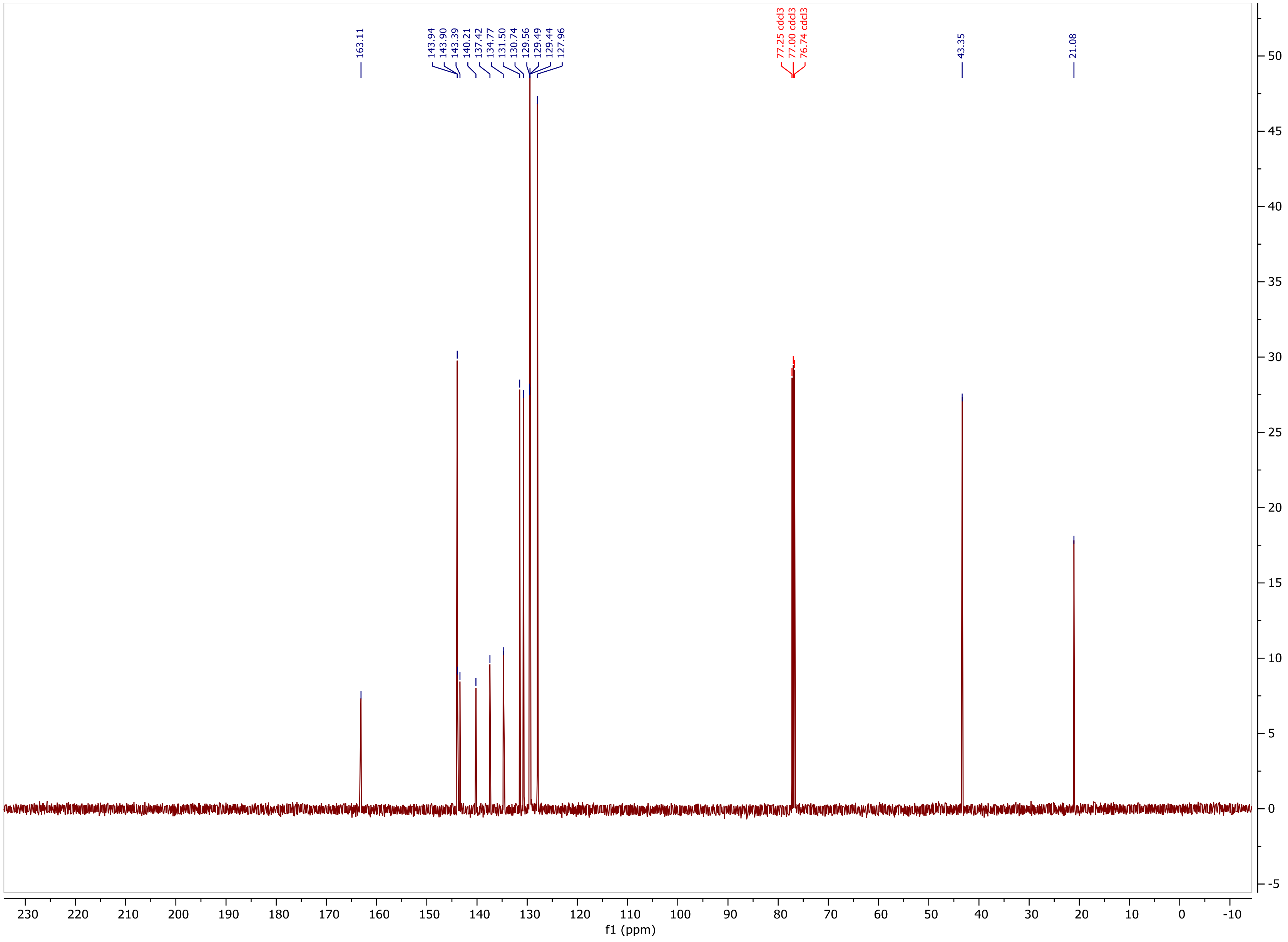
163.11
143.94
143.90
143.39
140.21
137.42
134.77
131.50
130.74
129.56
129.49
129.44
127.96
77.25 cdcl3
77.00 cdcl3
76.74 cdcl3
43.35
21.08
f1 (ppm)

Compound 25

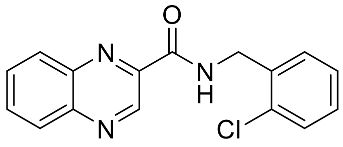

N-(2-chlorobenzyl)quinoxaline-2-carboxamide. M.p.: 204.4–206.7˚C. Yield: 28%. Light yellow powder. ^{1}H-NMR (500 MHz, DMSO-d_6) δ 9.46 (t, *J* = 6.2 Hz, 1H, amide), 9.21 (s, 1H, pyrazine), 8.93–8.86 (m, 1H, aromatic), 8.79–8.73 (m, 1H, aromatic), 7.53–7.39 (m, 2H, aromatic), 7.39–7.24 (m, 4H, aromatic), 4.59 (d, *J* = 6.2 Hz, 2H, methylene).^{13}C-NMR (126 MHz, DMSO-d_6) δ 163.42, 147.84, 144.77, 143.78, 143.60, 136.10, 131.98, 129.25, 128.74, 128.61, 127.32, 40.47. IR (ATR-Ge, cm^{-1}): 3378 (NH, CONH), 3098 (CH_2), 1715 (CO, CONH), 1663, 1581, 1523 (aromatic). Calculated for $C_{16}H_{12}ClN_3O$ (297.74 g/mol): C, 64.54%; H, 4.06%; N, 14.11%. Found: C, 64.17%; H, 4.02%; N, 13.95%. CAS# 878110-49-7.

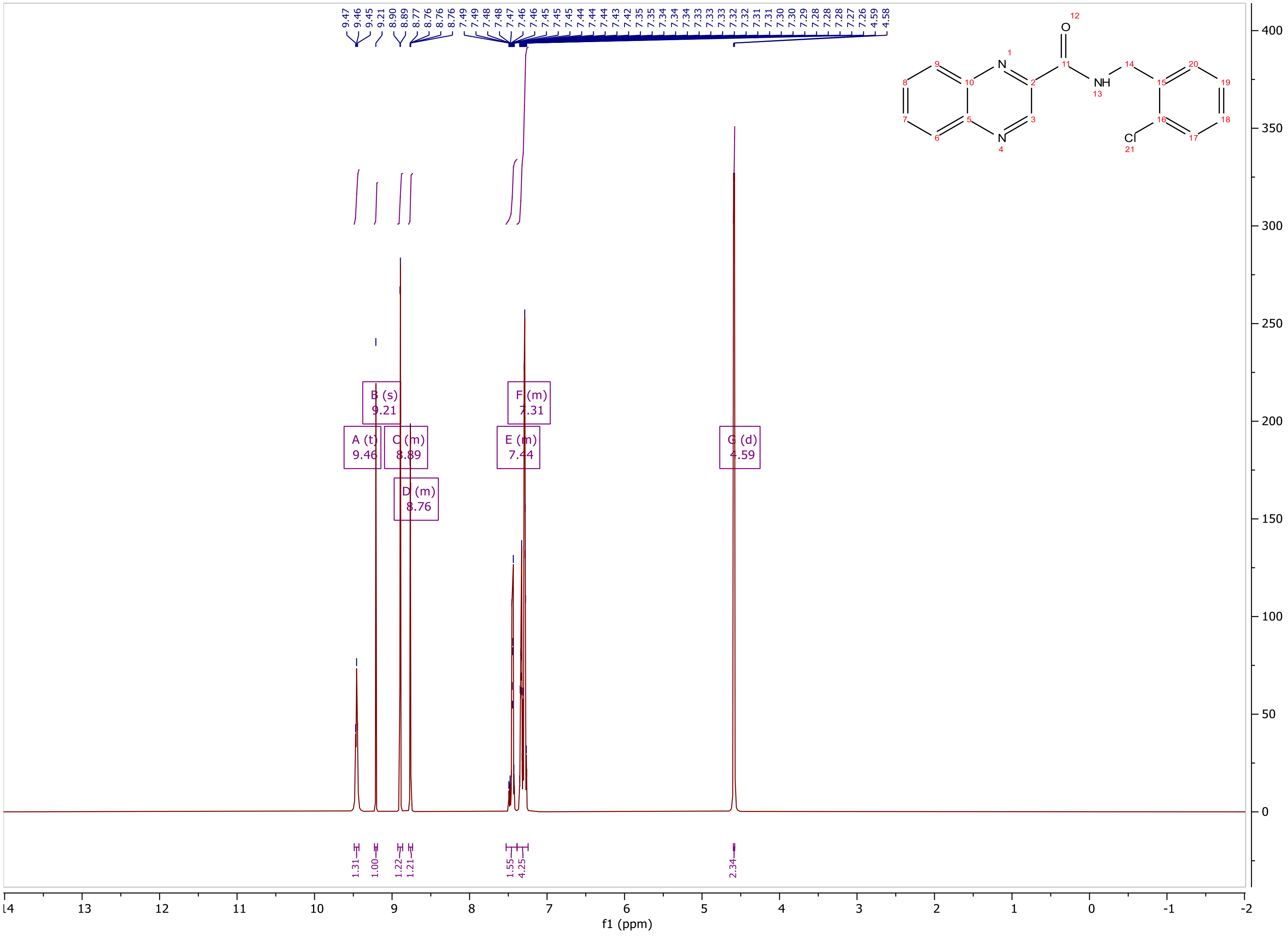

9.47
9.46
9.45
9.21
8.90
8.89
8.77
8.76
8.76
8.76
7.49
7.49
7.48
7.48
7.47
7.46
7.46
7.45
7.45
7.45
7.44
7.44
7.44
7.43
7.42
7.35
7.35
7.34
7.34
7.34
7.33
7.33
7.33
7.32
7.32
7.31
7.31
7.30
7.30
7.29
7.28
7.28
7.28
7.27
7.26
4.59
4.58
A (t)
9.46
B (s)
9.21
C (m)
8.89
D (m)
8.76
E (m)
7.44
F (m)
7.31
G (d)
4.59
1.31
1.00
1.22
1.21
1.55
4.25
2.34
f1 (ppm)
O
N
NH
N
Cl

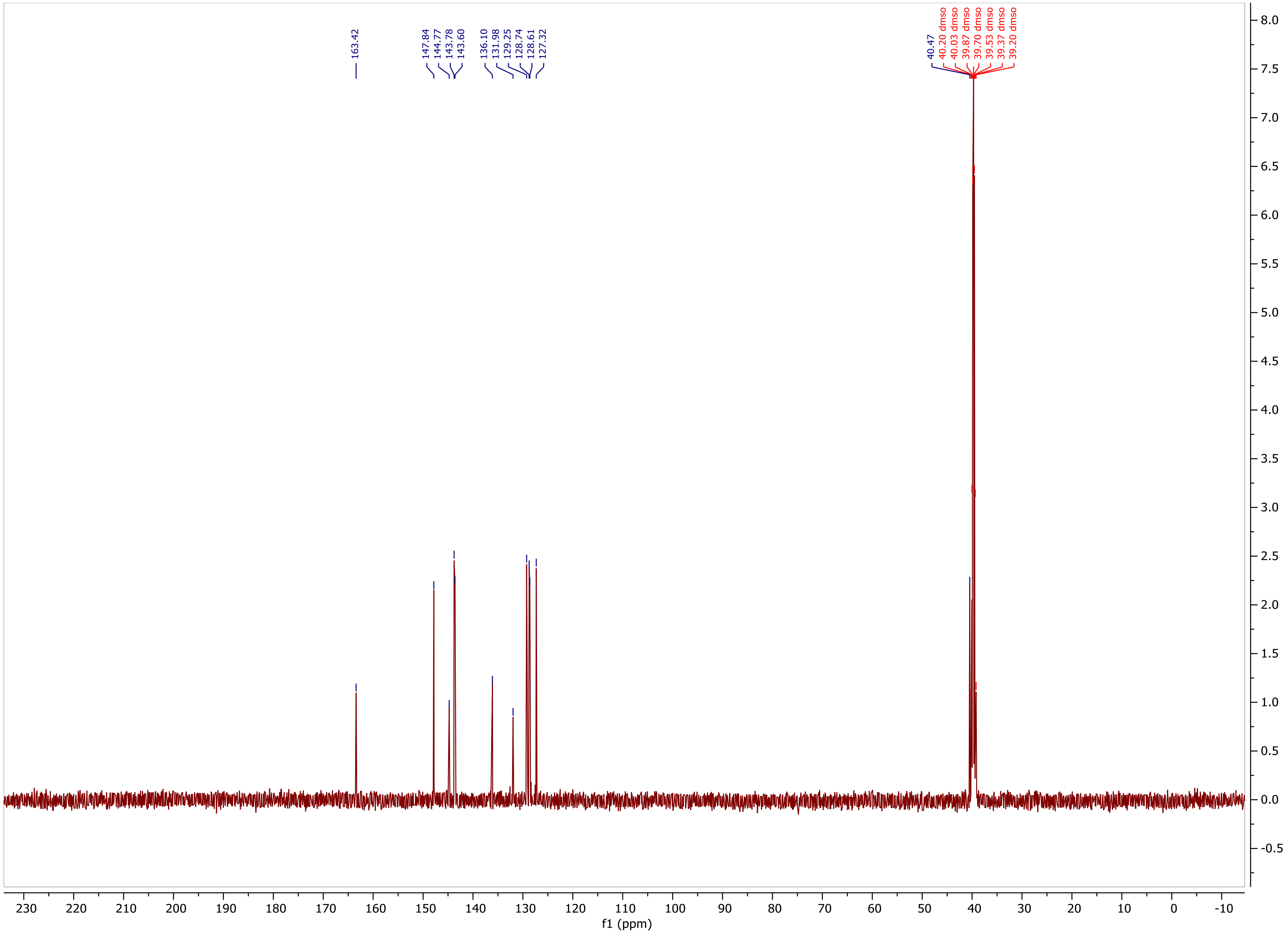

163.42
147.84
144.77
143.78
143.60
136.10
131.98
129.25
128.74
128.61
127.32
40.47
40.20 dmso
40.03 dmso
39.87 dmso
39.70 dmso
39.53 dmso
39.37 dmso
39.20 dmso
f1 (ppm)

Compound **26**

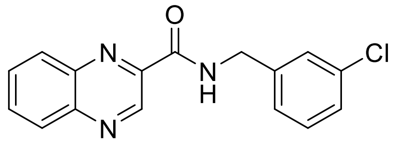

***N*-(3-chlorobenzyl)quinoxaline-2-carboxamide**. M.p.: 205.3–207.2˚C. Yield: 53%. Light yellow crystals. ^{1}H-NMR (500 MHz, DMSO-d_6) δ 9.81 (t, *J* = 6.4 Hz, 1H, amide), 9.48 (s, 1H, pyrazine), 8.22–8.18 (m, 2H, aromatic), 8.15–8.07 (m, 1H, aromatic), 8.02–7.98 (m, 2H, aromatic), 7.88–7.83 (m, 1H, aromatic), 7.67–7.60 (m, 2H, aromatic), 4.69 (d, *J* = 6.3 Hz, 2H, methylene). ^{13}C-NMR (151 MHz, DMSO-d_6) δ 163.91, 144.80, 144.35, 143.55, 142.35, 140.36, 133.50, 132.45, 131.83, 130.74, 129.96, 129.67, 127.90, 127.39, 126.77, 42.58. IR (ATR-Ge, cm^{-1}): 3389 (NH, CONH), 2938 (CH_2), 1673 (CO, CONH), 1599, 1574, 1521 (aromatic). Calculated for $C_{16}H_{12}ClN_3O$ (297.74 g/mol): C, 64.54%; H, 4.06%; N, 14.11%. Found: C, 64.37%; H, 4.06%; N, 13.95%. CAS# 1794995-19-9.

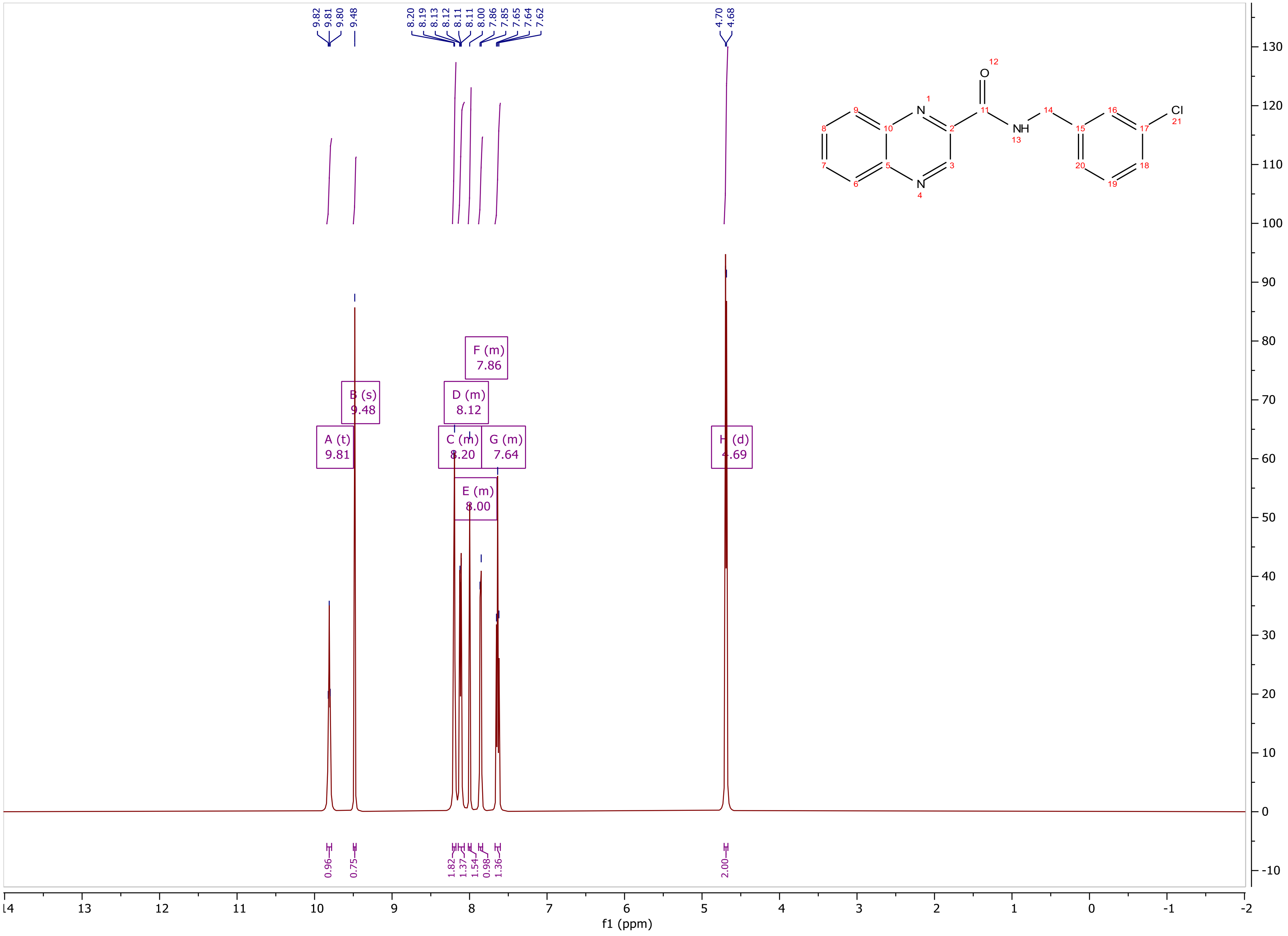

9.82
9.81
9.80
9.48
8.20
8.19
8.13
8.12
8.11
8.11
8.00
7.86
7.85
7.65
7.64
7.62
4.70
4.68
A (t)
9.81
B (s)
9.48
C (m)
8.20
D (m)
8.12
E (m)
8.00
F (m)
7.86
G (m)
7.64
H (d)
4.69
0.96
0.75
1.82
1.37
1.54
0.98
1.36
2.00
O
N
NH
N
Cl
f1 (ppm)

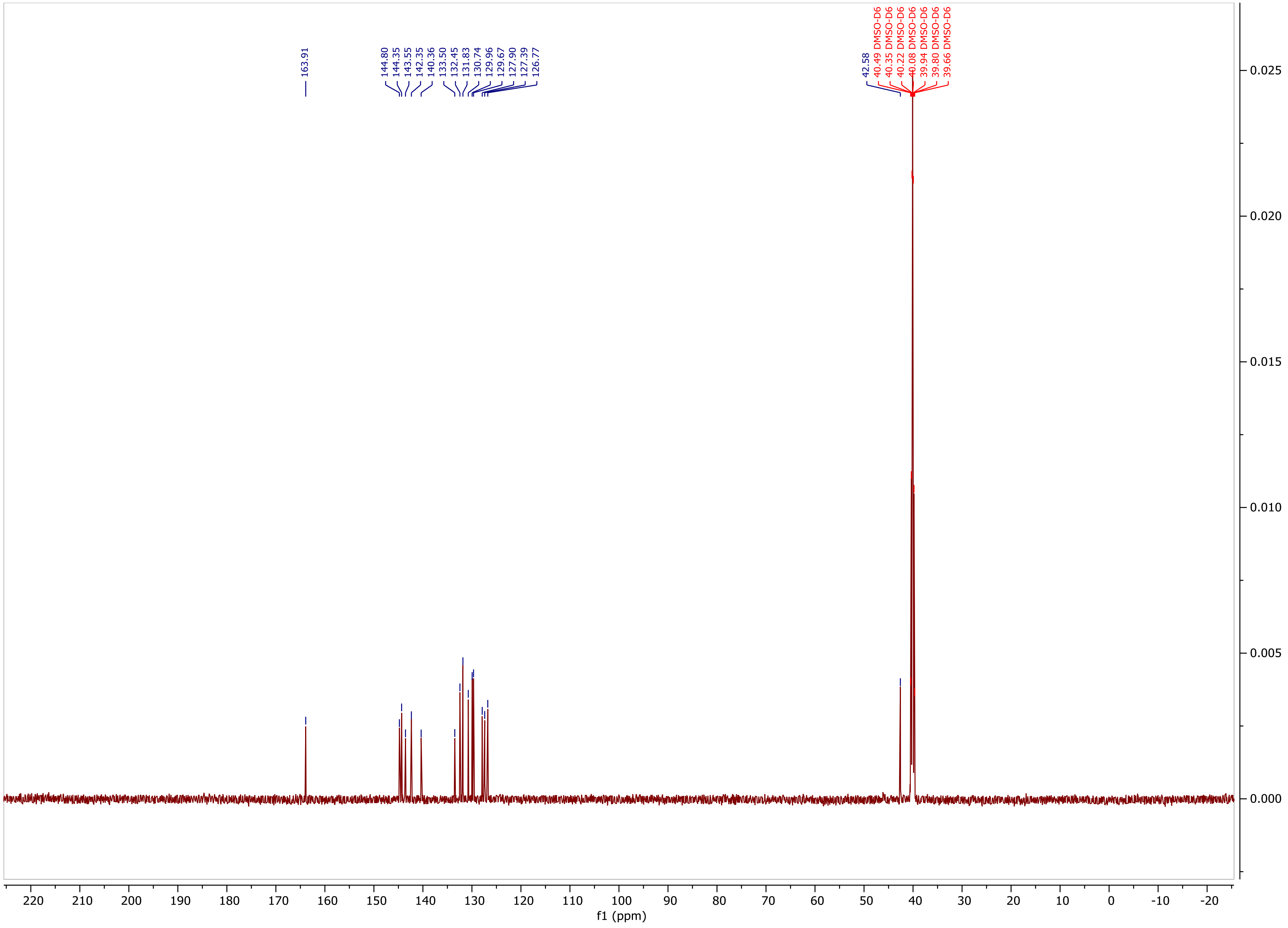
163.91
144.80
144.35
143.55
142.35
140.36
133.50
132.45
131.83
130.74
129.96
129.67
127.90
127.39
126.77
42.58
40.49 DMSO-D6
40.35 DMSO-D6
40.22 DMSO-D6
40.08 DMSO-D6
39.94 DMSO-D6
39.80 DMSO-D6
39.66 DMSO-D6
f1 (ppm)

Compound 27

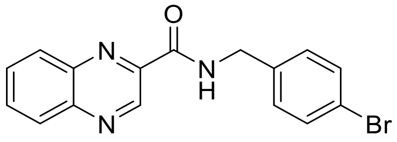

N-(4-bromobenzyl)quinoxaline-2-carboxamide. M.p.: 203.4–204.1˚C. Yield: 40%. Beige powder. ^{1}H-NMR (600 MHz, DMSO-d_6) δ 9.66 (t, *J* = 6.4 Hz, 1H, amide), 9.48 (s, 1H, pyrazine), 8.23–8.15 (m, 2H, aromatic), 8.01–7.94 (m, 2H, aromatic), 7.54–7.48 (m, 2H, aromatic), 7.37–7.32 (m, 2H, aromatic), 4.53 (d, *J* = 6.3 Hz, 2H, methylene). ^{13}C-NMR (151 MHz, DMSO-d_6) δ 163.47, 144.44, 143.93, 143.16, 139.97, 138.86, 132.08, 131.48, 131.32, 129.93, 129.57, 129.29, 120.08, 42.13. IR (ATR-Ge, cm^{-1}): 3370 (NH, CONH), 2980 (CH_2), 1670 (CO, CONH), 1575, 1530, 1487 (aromatic). Calculated for $C_{16}H_{12}BrN_3O$ (342.20 g/mol): C, 56.16; H, 3.53%; N, 12.28%. Found: C, 56.23%; H, 3.50%; N, 12.22%. CAS# 1240104-06-6.

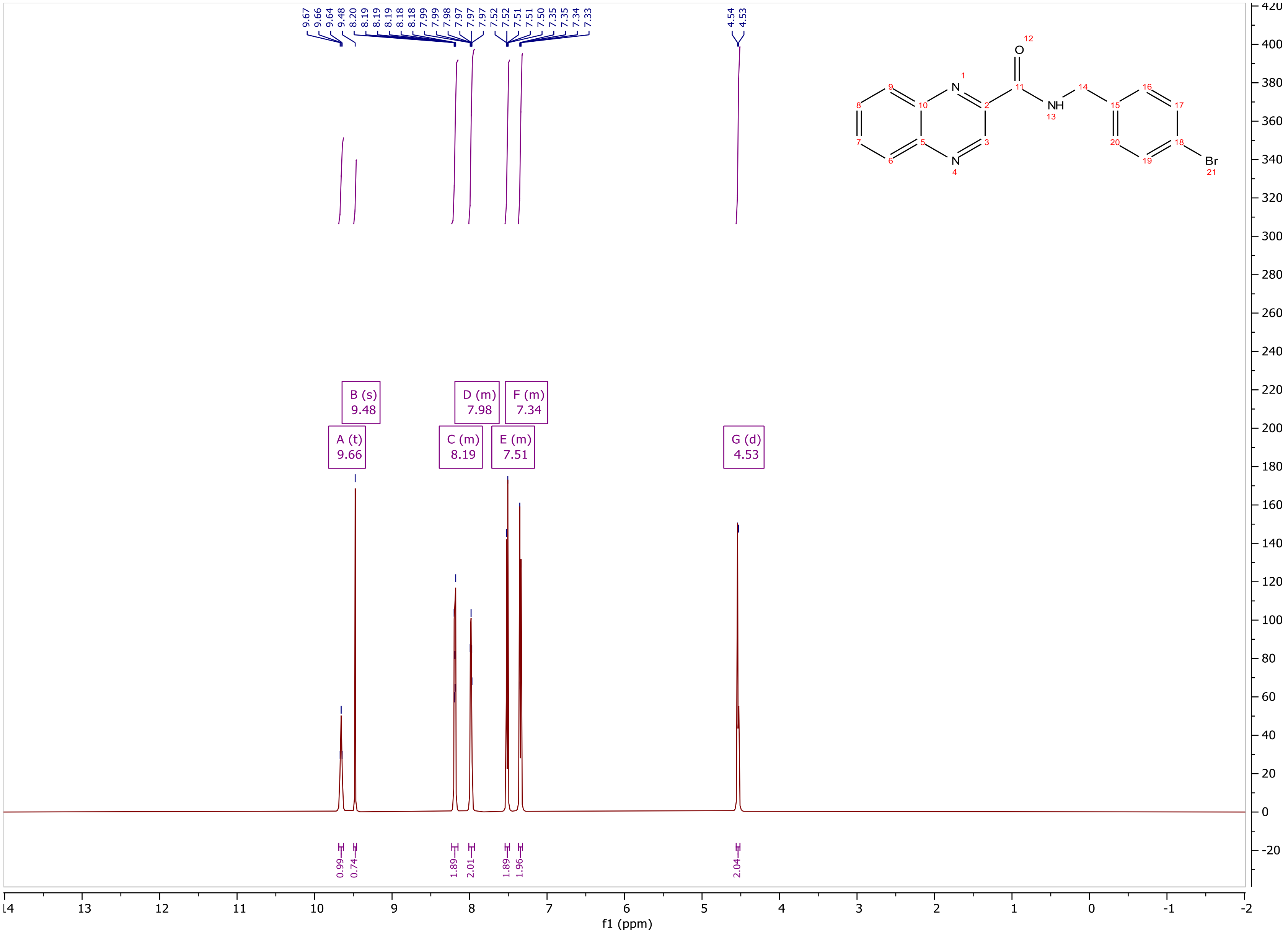

9.67
9.66
9.64
9.48
8.20
8.19
8.19
8.19
8.18
8.18
7.99
7.99
7.98
7.97
7.97
7.97
7.52
7.52
7.51
7.51
7.50
7.35
7.35
7.35
7.34
7.33
4.54
4.53
A (t) 9.66
B (s) 9.48
C (m) 8.19
D (m) 7.98
E (m) 7.51
F (m) 7.34
G (d) 4.53
0.99
0.74
1.89
2.01
1.89
1.96
2.04
f1 (ppm)
O
N
NH
N
Br

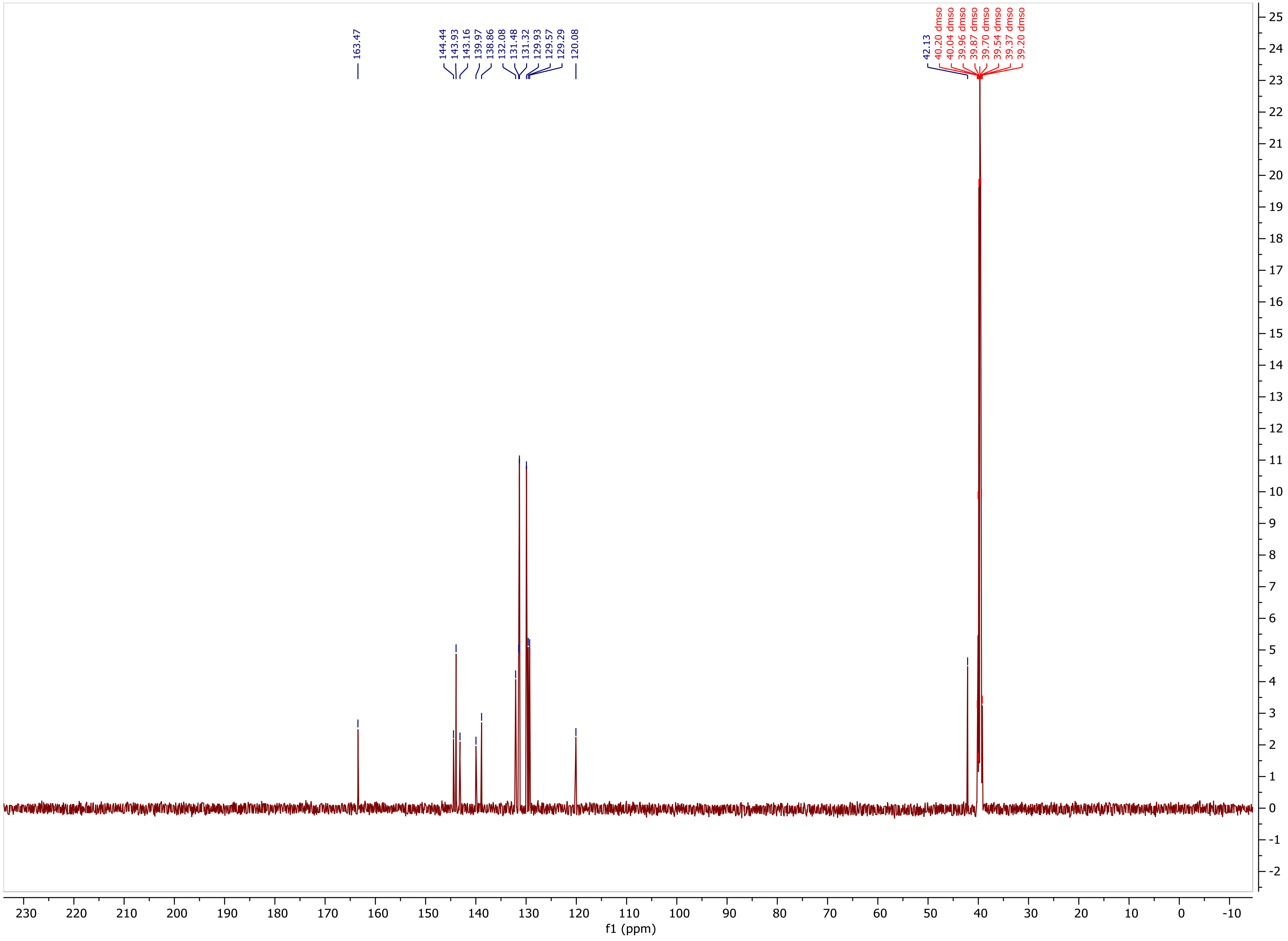

163.47
144.44
143.93
143.16
139.97
138.86
132.08
131.48
131.32
129.93
129.57
129.29
120.08
42.13
40.20 dmso
40.04 dmso
39.96 dmso
39.87 dmso
39.70 dmso
39.54 dmso
39.37 dmso
39.20 dmso
f1 (ppm)

Compound 28

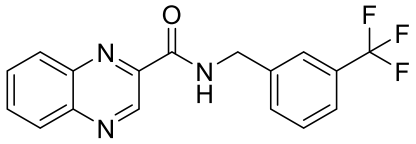

N-(3-(trifluoromethyl)benzyl)quinoxaline-2-carboxamide. M.p.: 211.3–213.4˚C. Yield: 47%. Pale yellow powder. ^{1}H-NMR (500 MHz, DMSO-d_6) δ 9.74 (t, J = 6.4 Hz, 1H, amide), 9.48 (s, 1H, pyrazine), 8.22–8.17 (m, 2H, aromatic), 8.01–7.95 (m, 2H, aromatic), 7.77–7.73 (m, 1H, aromatic), 7.73–7.68 (m, 1H, aromatic), 7.67–7.54 (m, 2H, aromatic), 4.66 (d, J = 6.3 Hz, 2H, methylene). ^{13}C-NMR (126 MHz, DMSO-$_{d6}$): δ 163.60, 144.39, 143.95, 143.18, 140.89, 139.97, 132.07, 131.90, 131.46, 129.56, 129.28, 129.18 (q, J = 31.7Hz), 124.42 (q, J = 272.4 Hz), 124.29 (q, J = 3.7 Hz), 123.82 (q, J = 3.5 Hz), 42.35. IR (ATR-Ge, cm^{-1}): 3362 (NH, CONH), 2921 (CH_2), 1663 (CO, CONH), 1615, 1596, 1571 (aromatic). Calculated for $C_{17}H_{12}F_3N_3O$ (331.30 g/mol): C, 61.63%; H, 3.56%; N, 12.68%.

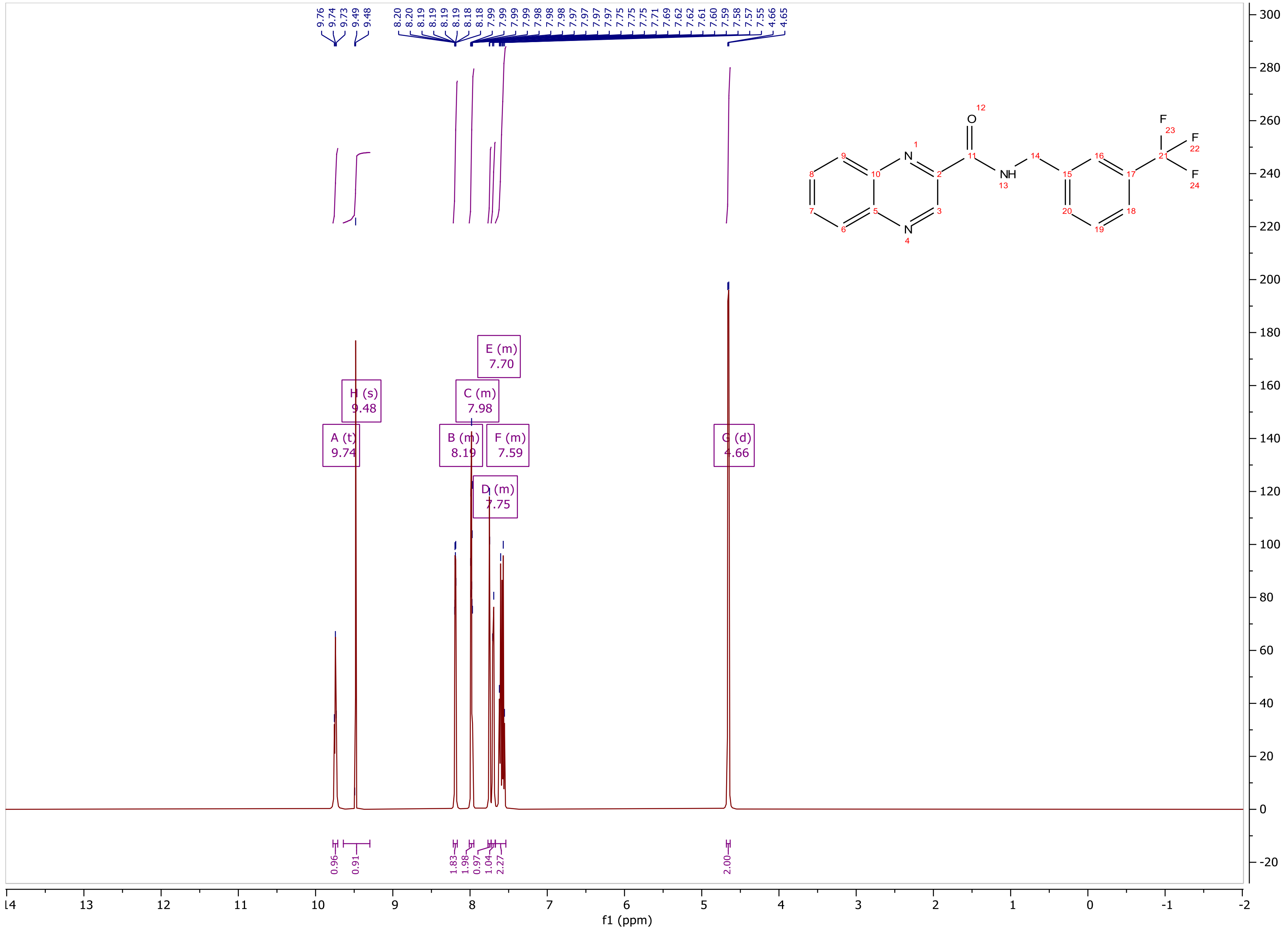

A (t)
9.74
H (s)
9.48
B (m)
8.19
C (m)
7.98
D (m)
7.75
E (m)
7.70
F (m)
7.59
G (d)
4.66
f1 (ppm)

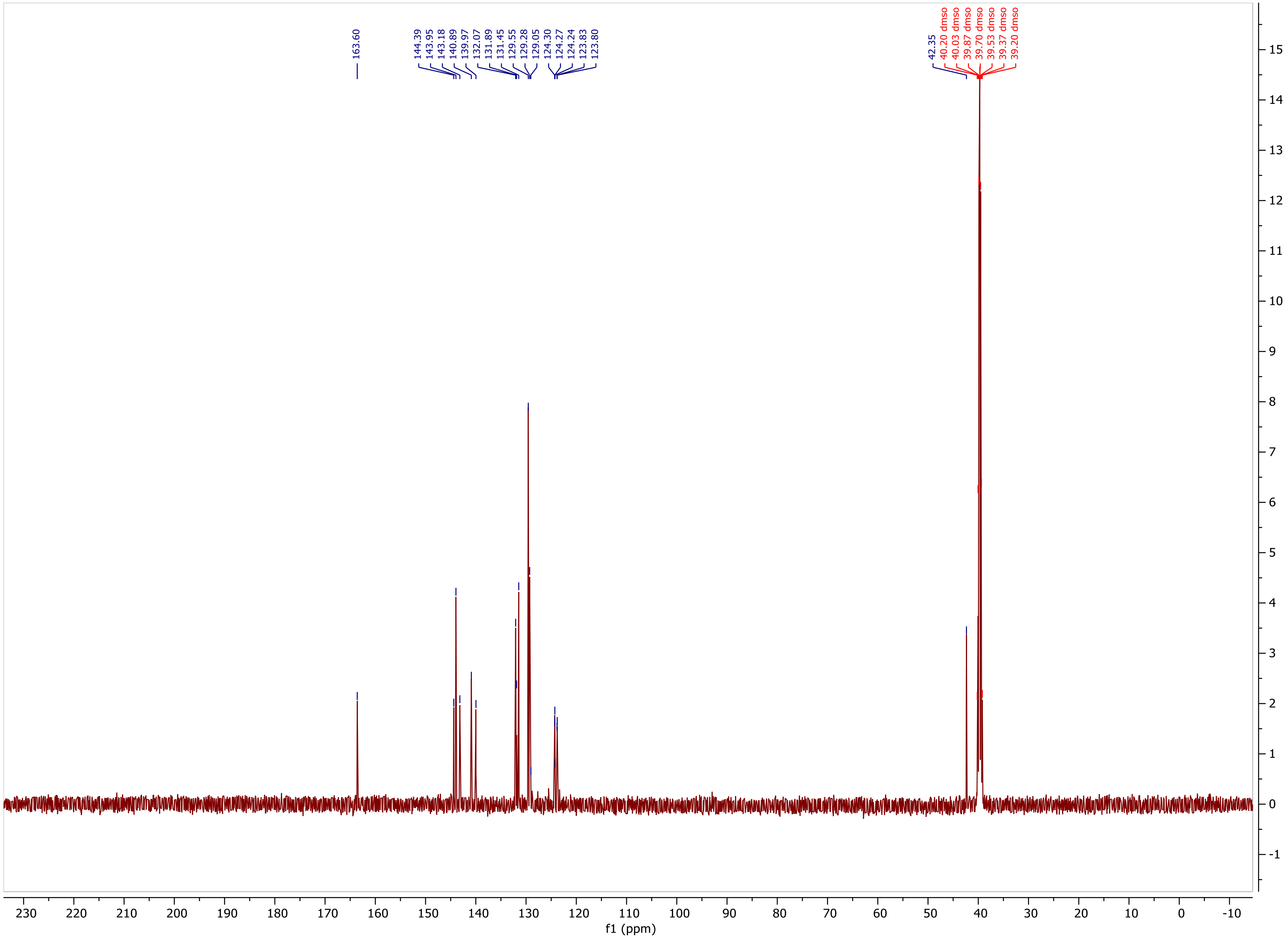

163.60
144.39
143.95
143.18
140.89
139.97
132.07
131.89
131.45
129.55
129.28
129.05
124.30
124.27
124.24
123.83
123.80
42.35
40.20 dmso
40.03 dmso
39.87 dmso
39.70 dmso
39.53 dmso
39.37 dmso
39.20 dmso
f1 (ppm)

Compound 29

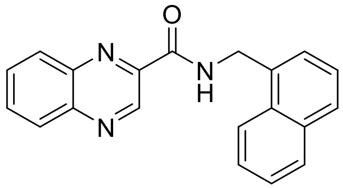

N-(naphthalen-1-ylmethyl)quinoxaline-2-carboxamide. M.p.: 281.9–285.2˚C. Yield: 37%. Yellow-white powder. ^{1}H-NMR (500 MHz, DMSO-d_6) δ 9.60 (t, J = 6.2 Hz, 1H, amide), 9.52 (s, 1H, pyrazine), 8.31–8.26 (m, 1H, aromatic), 8.23–8.15 (m, 2H, aromatic), 8.00–7.93 (m, 3H, aromatic), 7.88–7.83 (m, 1H, aromatic), 7.62–7.51 (m, 3H, aromatic), 7.51–7.44 (m, 1H, aromatic), 5.06 (d, J = 6.1 Hz, 2H, methylene). ^{13}C-NMR (126 MHz, DMSO-d_6) δ 163.39, 144.50, 143.97, 143.15, 139.96, 134.33, 133.46, 132.05, 131.43, 131.00, 129.58, 129.26, 128.69, 127.75, 126.46, 125.95, 125.79, 125.59, 123.65, 40.70. IR (ATR-Ge, cm^{-1}): 3369 (NH, CONH), 2921 (CH_2), 1677 (CO, CONH), 1625, 1600, 1574 (aromatic). Calculated for $C_{20}H_{15}N_3O$ (313.36 g/mol): C, 77.04%; H, 5.23%; N, 12.84%. Found: C, 77.16%; H, 5.28%; N, 12.85%. CAS# 1444225-34-6.

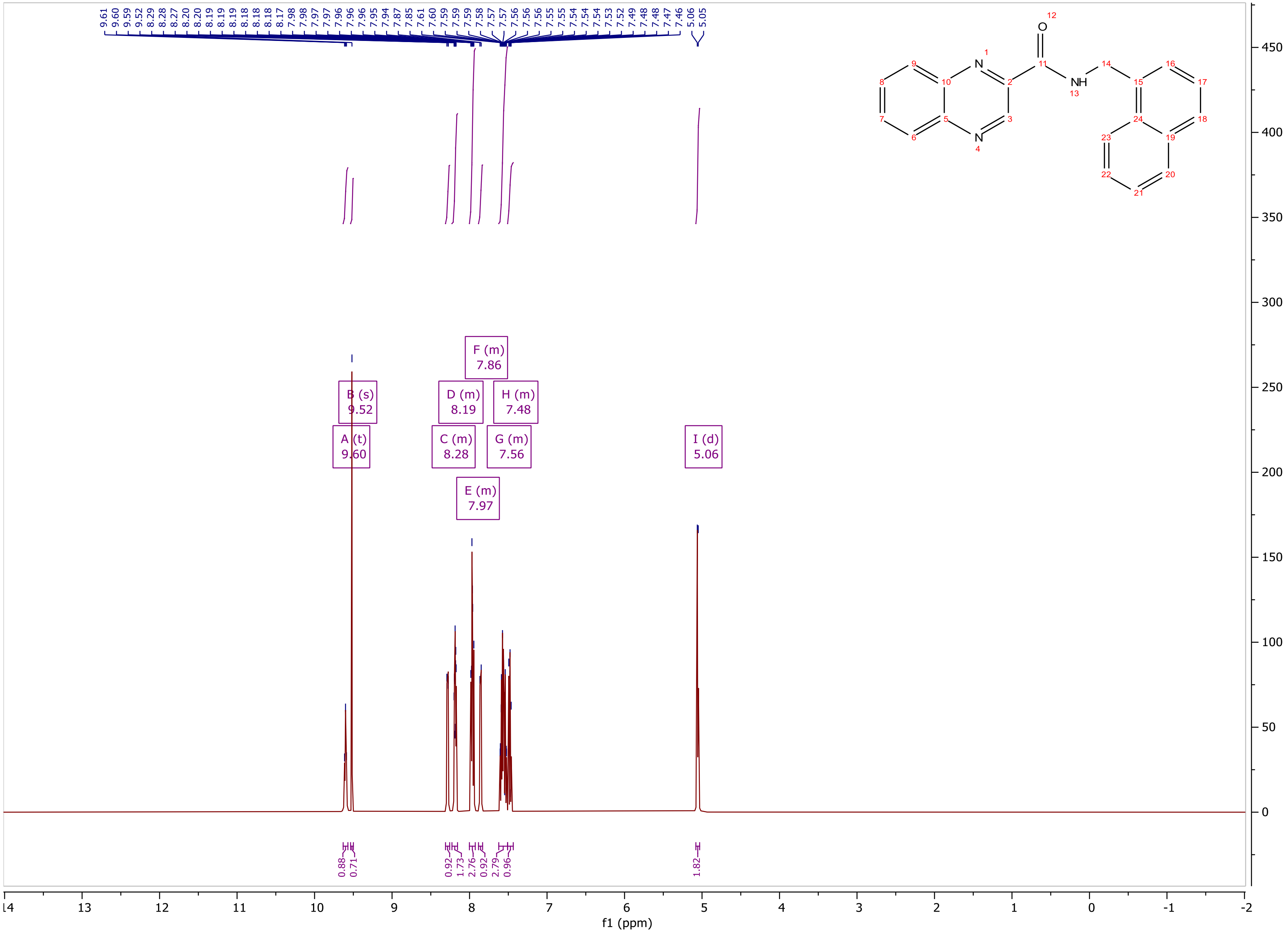

A (t)
9.60
B (s)
9.52
C (m)
8.28
D (m)
8.19
E (m)
7.97
F (m)
7.86
G (m)
7.56
H (m)
7.48
I (d)
5.06
f1 (ppm)

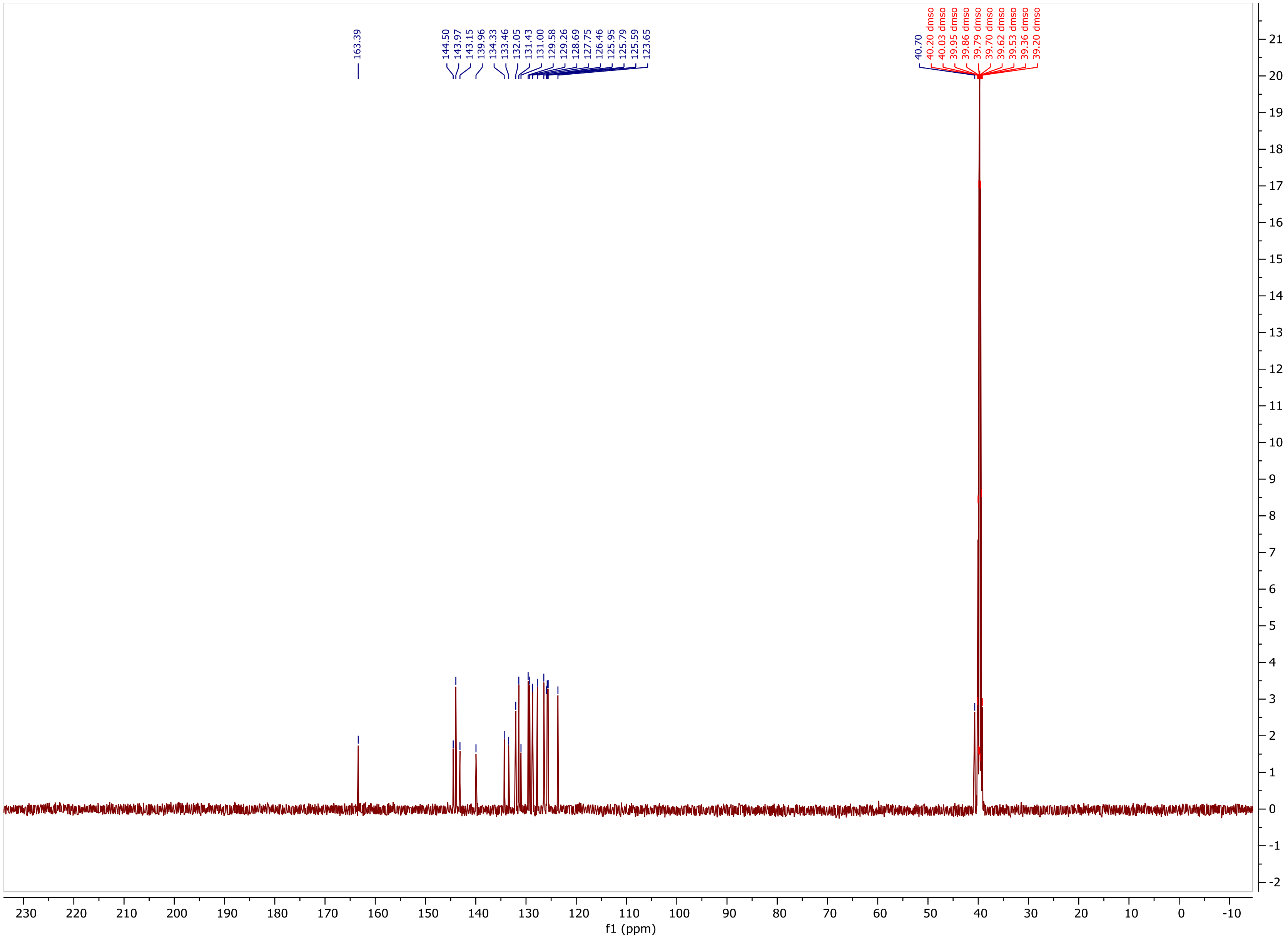
163.39
144.50
143.97
143.15
139.96
134.33
133.46
132.05
131.43
131.00
129.58
129.26
128.69
127.75
126.46
125.95
125.79
125.59
123.65
40.70
40.20 dmso
40.03 dmso
39.95 dmso
39.86 dmso
39.79 dmso
39.70 dmso
39.62 dmso
39.53 dmso
39.36 dmso
39.20 dmso
f1 (ppm)

Compound 30

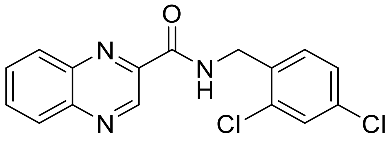

***N*-(2,4-dichlorobenzyl)quinoxaline-2-carboxamide**. M.p.: 205.5–206.7˚C. Yield: 31%. Light beige powder. ^{1}H-NMR (600 MHz, DMSO-d_6) δ 9.62 (t, *J* = 6.2 Hz, 1H, amide), 9.45 (s, 1H, pyrazine), 8.21–8.14 (m, 2H, aromatic), 8.00–7.93 (m, 2H, aromatic), 7.59 (d, *J* = 2.1 Hz, 1H, aromatic), 7.42–7.34 (m, 2H, aromatic), 4.58 (d, *J* = 6.2 Hz, 2H, methylene). ^{13}C-NMR (151 MHz, DMSO-d_6) δ 164.12, 144.64, 144.32, 143.61, 140.37, 135.76, 133.33, 132.78, 132.55, 131.91, 130.62, 129.99, 129.69, 129.10, 127.89, 40.60. IR (ATR-Ge, cm^{-1}): 3361 (NH, CONH), 2922 (CH_2), 1668 (CO, CONH), 1629, 1589, 1571 (aromatic). Calculated for $C_{15}H_{17}N_3O$ (255.32 g/mol): C, 70.56; H, 6.71%; N, 16.46%. Found: C, 70.42%; H, 6.67%; N, 16.71%. CAS# 155129-19-4.

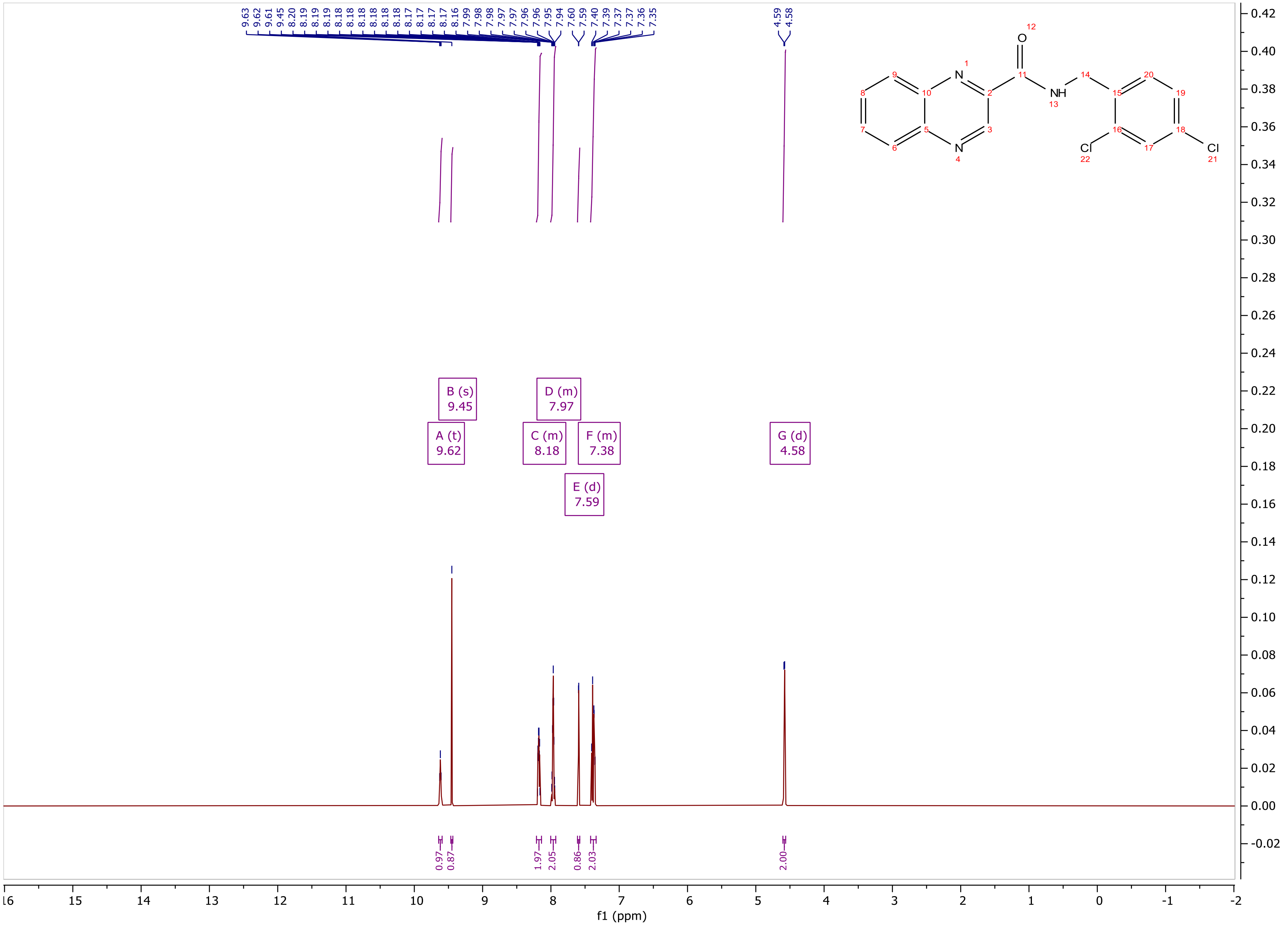

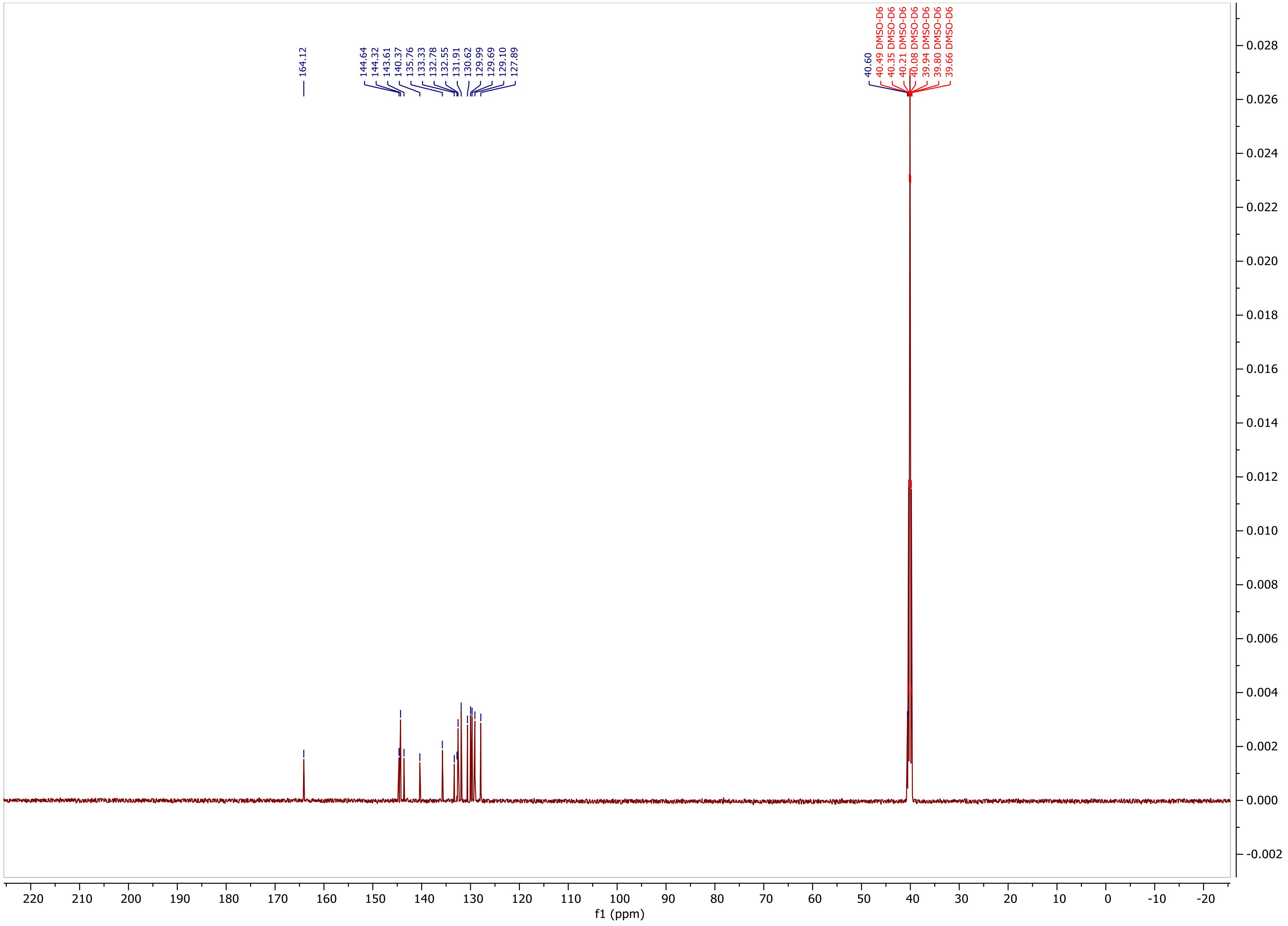
164.12
144.64
144.32
143.61
140.37
135.76
133.33
132.78
132.55
131.91
130.62
129.99
129.69
129.10
127.89
40.60
40.49 DMSO-D6
40.35 DMSO-D6
40.21 DMSO-D6
40.08 DMSO-D6
39.94 DMSO-D6
39.80 DMSO-D6
39.66 DMSO-D6
f1 (ppm)

Compound 31

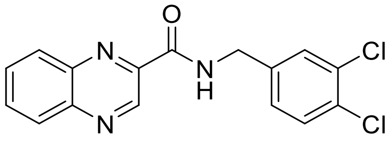

***N*-(3,4-dichlorobenzyl)quinoxaline-2-carboxamide**. M.p.: 203.5–205.5˚C. Yield: 49%. Yellow solid. ^{1}H-NMR (500 MHz, DMSO-d_6) δ 9.69 (t, *J* = 6.4 Hz, 1H, amide), 9.47 (s, 1H, pyrazine), 8.22–8.15 (m, 2H, aromatic), 8.02–7.95 (m, 2H, aromatic), 7.65–7.61 (m, 1H, aromatic), 7.60–7.55 (m, 1H, aromatic), 7.42–7.35 (m, 1H, aromatic), 4.56 (d, *J* = 6.3 Hz, 2H, methylene). ^{13}C-NMR (126 MHz, DMSO-d_6) δ 163.57, 144.33, 143.94, 143.17, 140.62, 139.95, 132.06, 131.44, 131.00, 130.62, 129.70, 129.58, 129.55, 129.27, 128.08, 41.72. IR (ATR-Ge, cm^{-1}): 3361 (NH, CONH), 2922 (CH_2), 1668 (CO, CONH), 1629, 1589, 1571 (aromatic). Calculated for $C_{16}H_{11}Cl_2N_3O$ (332.18 g/mol): C, 57.85%; H, 3.34%; N, 12.65%. Found: C, 57.60%; H, 3.31%; N, 12.45%.

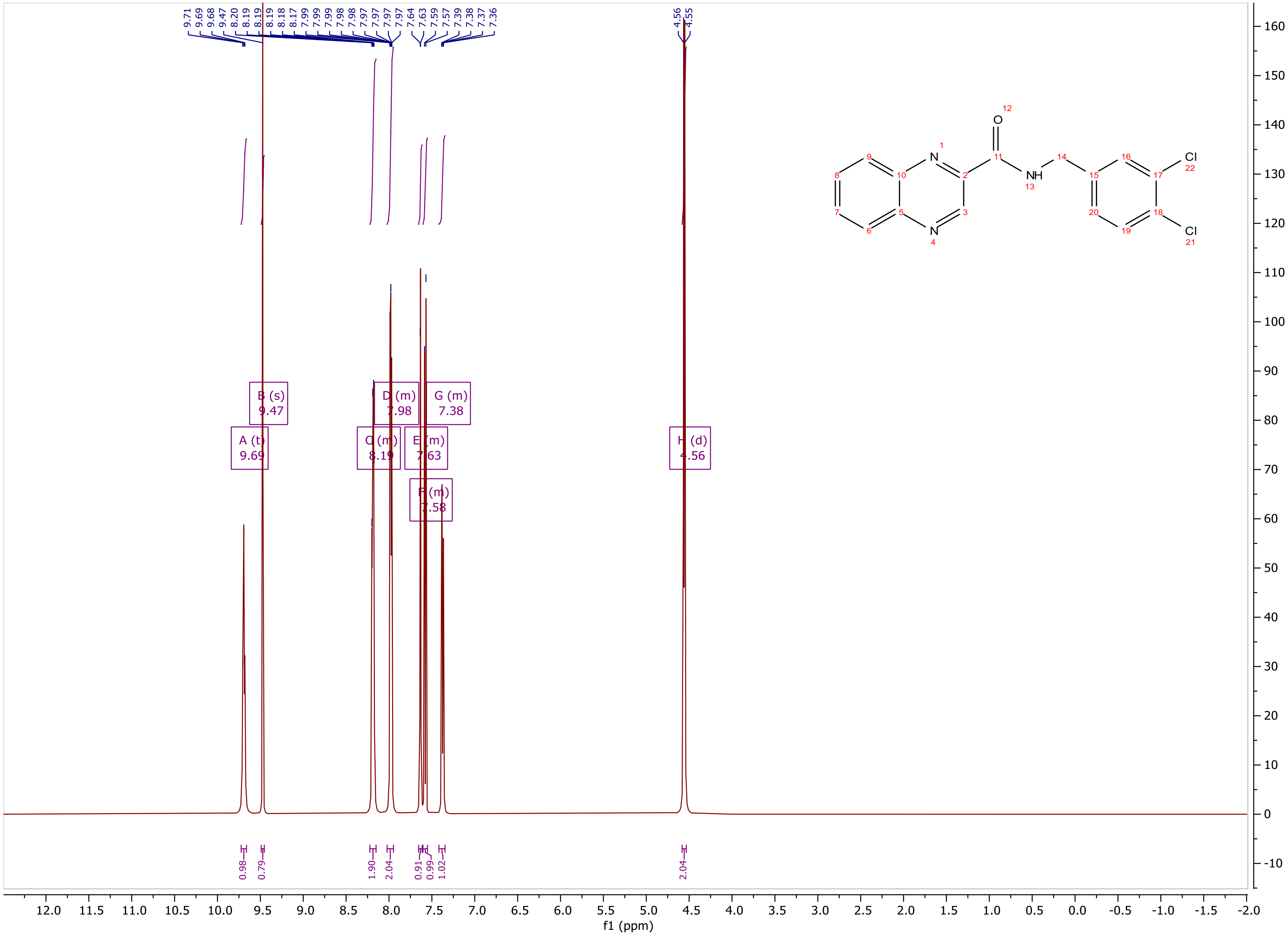
9.71 9.69 9.68 9.47 8.20 8.19 8.19 8.19 8.18 8.17 7.99 7.99 7.98 7.98 7.97 7.97 7.97 7.97 7.64 7.63 7.59 7.57 7.39 7.38 7.37 7.36
4.56 4.55
A (t) 9.69
B (s) 9.47
C (m) 8.19
D (m) 7.98
E (m) 7.63
F (m) 7.58
G (m) 7.38
H (d) 4.56
0.98 0.79 1.90 2.04 0.91 0.99 1.02 2.04
f1 (ppm)
O
N
NH
Cl
Cl
N

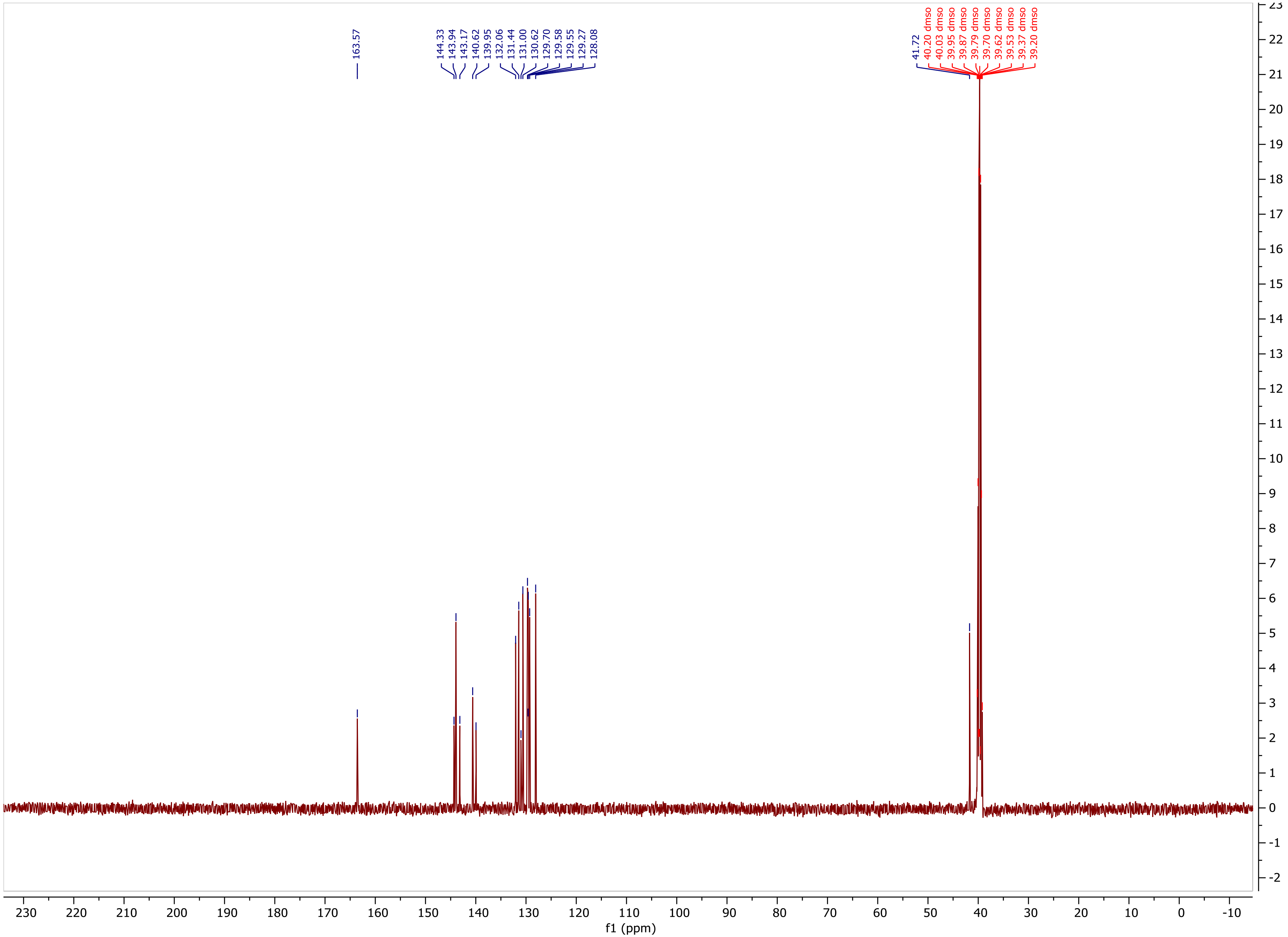
163.57
144.33
143.94
143.17
140.62
139.95
132.06
131.44
131.00
130.62
129.70
129.58
129.55
129.27
128.08
41.72
40.20 dmso
40.03 dmso
39.95 dmso
39.87 dmso
39.79 dmso
39.70 dmso
39.62 dmso
39.53 dmso
39.37 dmso
39.20 dmso
f1 (ppm)

Compound 32

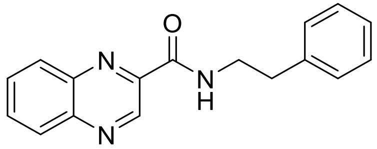

***N*-phenethylquinoxaline-2-carboxamide**. M.p.: 222.4–223.1˚C. Yield: 67%. White powder. ^{1}H-NMR (600 MHz, DMSO-d_6) δ 9.00 (s, 1H, pyrazine), 8.66 (t, J = 6.4 Hz, 1H, amide), 7.83–7.77 (m, 1H, aromatic), 7.71–7.66 (m, 1H, aromatic), 7.29–7.27 (m, 2H, aromatic), 7.22–7.20 (m, 5H, aromatic), 2.97–2.95 (m, 2H, methylene), 2.88–2.86 (m, 2H, methylene).^{13}C-NMR (151 MHz, DMSO-d_6) δ 168.05, 150.96, 148.96, 138.01, 136.60, 130.32, 130.15, 129.17, 129.13, 128.51, 127.22, 126.09, 124.92, 122.50, 33.46. IR (ATR-Ge, cm^{-1}): 3370 (NH, CONH), 2976 (CH_2), 1601 (CO, CONH), 1498, 1477, 1465 (aromatic). Calculated for $C_{17}H_{15}N_3O$ (277.33 g/mol): C, 73.63; H, 5.45%; N, 15.15%. Found: C, 73.44%; H, 5.37%; N, 14.92%. CAS#112369-27-4.

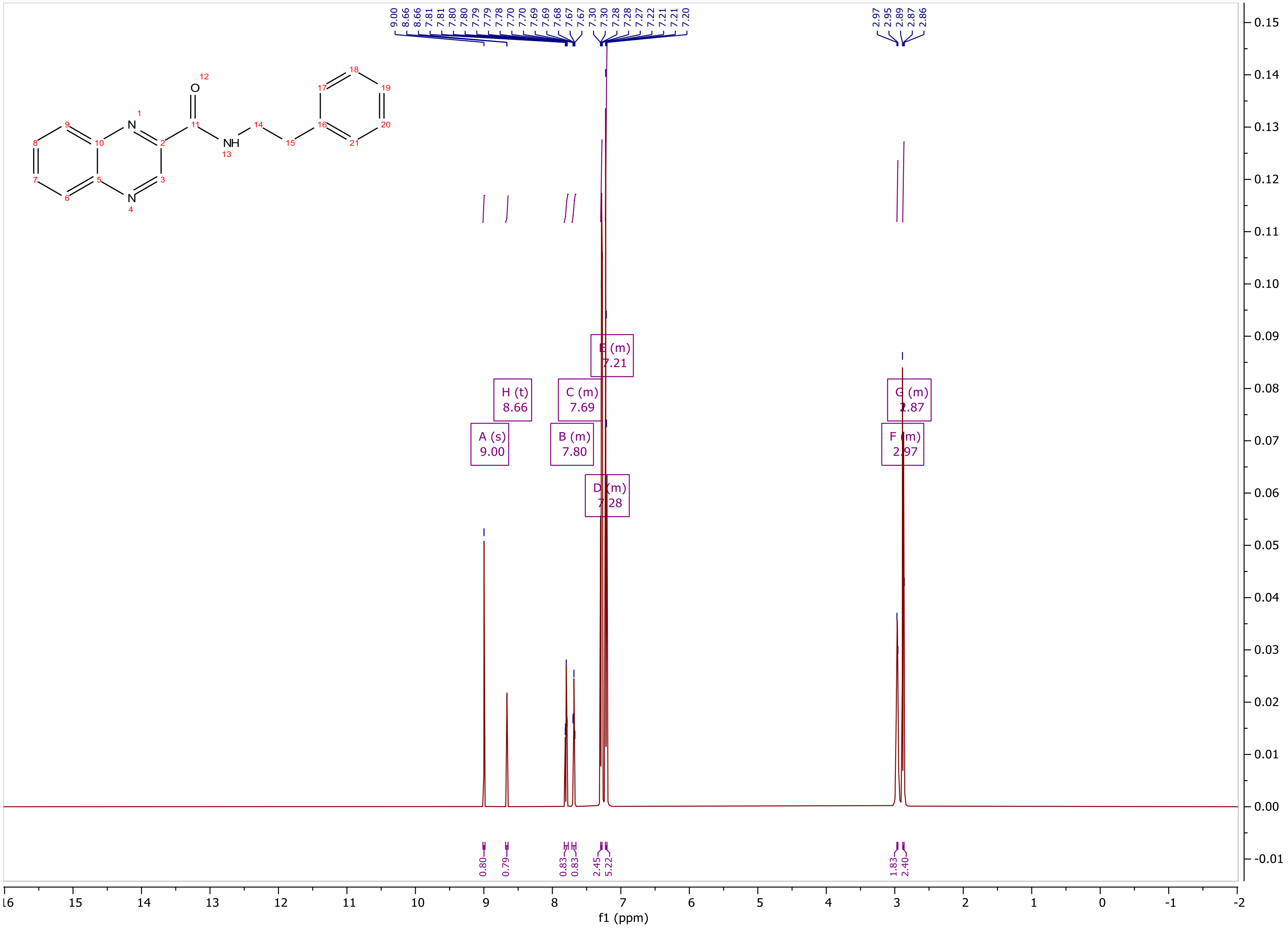

9.00 8.66 8.66 7.81 7.81 7.80 7.80 7.79 7.79 7.78 7.70 7.70 7.69 7.69 7.68 7.67 7.67 7.30 7.30 7.28 7.28 7.27 7.22 7.21 7.21 7.20
2.97 2.95 2.89 2.87 2.86
A (s) 9.00
H (t) 8.66
B (m) 7.80
C (m) 7.69
D (m) 7.28
E (m) 7.21
F (m) 2.97
G (m) 2.87
0.80 0.79 0.83 0.83 2.45 5.22 1.83 2.40
f1 (ppm)

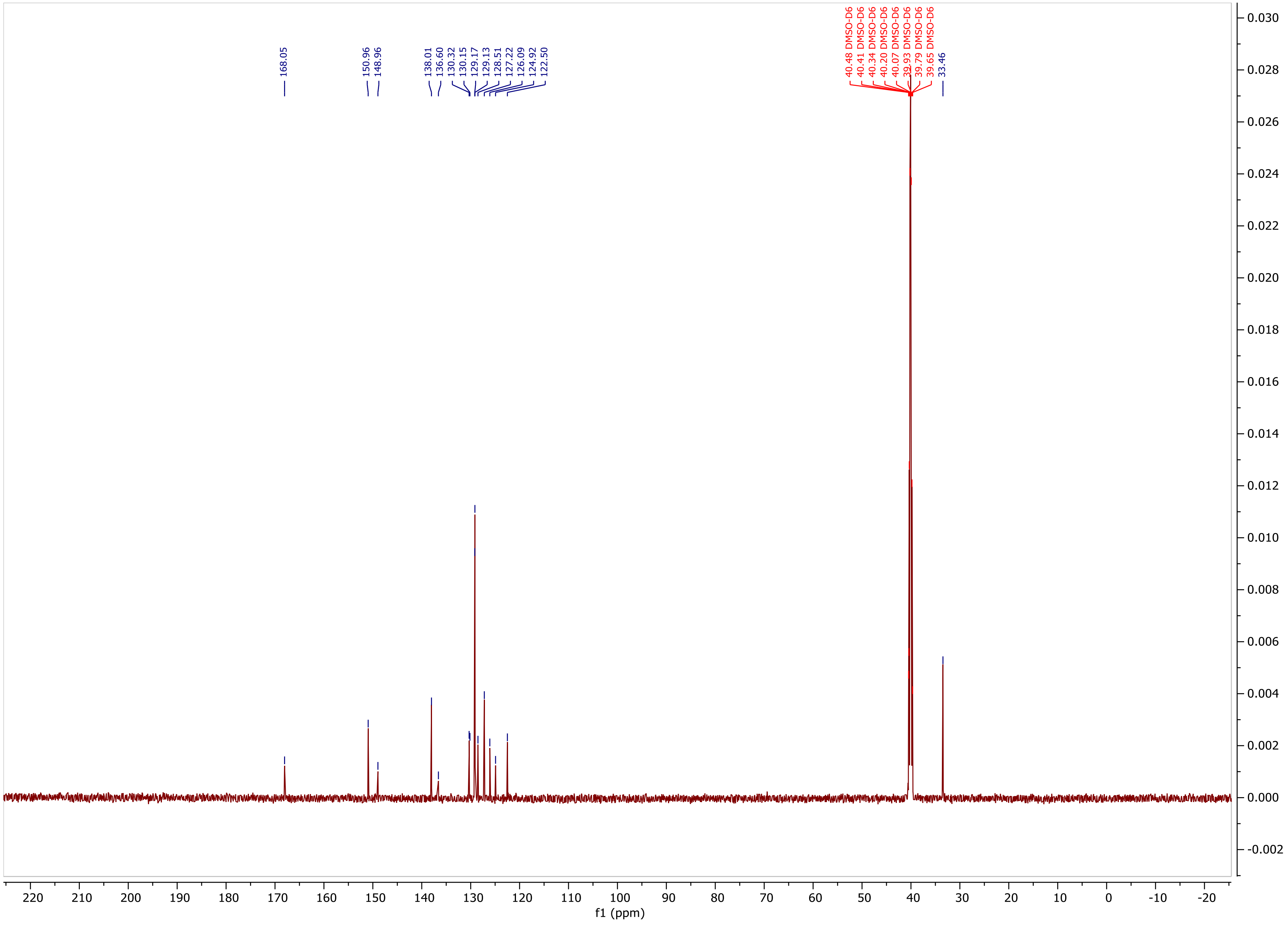

168.05
150.96
148.96
138.01
136.60
130.32
130.15
129.17
129.13
128.51
127.22
126.09
124.92
122.50
40.48 DMSO-D6
40.41 DMSO-D6
40.34 DMSO-D6
40.20 DMSO-D6
40.07 DMSO-D6
39.93 DMSO-D6
39.79 DMSO-D6
39.65 DMSO-D6
33.46
f1 (ppm)

Compound 33

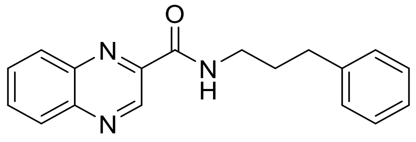

N-(3-phenylpropyl)quinoxaline-2-carboxamide. M.p.: 241.7–243.0˚C. Yield: 60%. White powder. ^{1}H-NMR (600 MHz, DMSO-d_6) δ 9.42 (s, 1H, pyrazine), 9.05 (t, *J* = 6.0 Hz, 1H, amide), 8.14 (m, 2H, aromatic), 7.97–7.90 (m, 2H, aromatic), 7.26–7.17 (m, 4H, aromatic), 7.16–7.09 (m, 1H, aromatic), 3.40–3.34 (m, 2H, methylene), 2.64–2.59 (m, 2H, methylene), 1.91–1.83 (m, 2H, methylene). ^{13}C-NMR (151 MHz, DMSO-d_6) δ 163.63, 145.06, 144.29, 143.44, 142.21, 140.33, 132.29, 131.75, 129.94, 129.63, 128.84, 128.81, 126.26, 39.32, 33.19, 31.30. IR (ATR-Ge, cm^{-1}): 3352 (NH, CONH), 2975 (CH_2), 1694 (CO, CONH), 1606, 1551, 1537 (aromatic). Calculated for $C_{18}H_{17}N_3O$ (291.35 g/mol): C, 74.20; H, 5.88%; N, 14.42%. Found: C, 74.45%; H, 5.67%; N, 14.22%. CAS# 1795027-08-5.

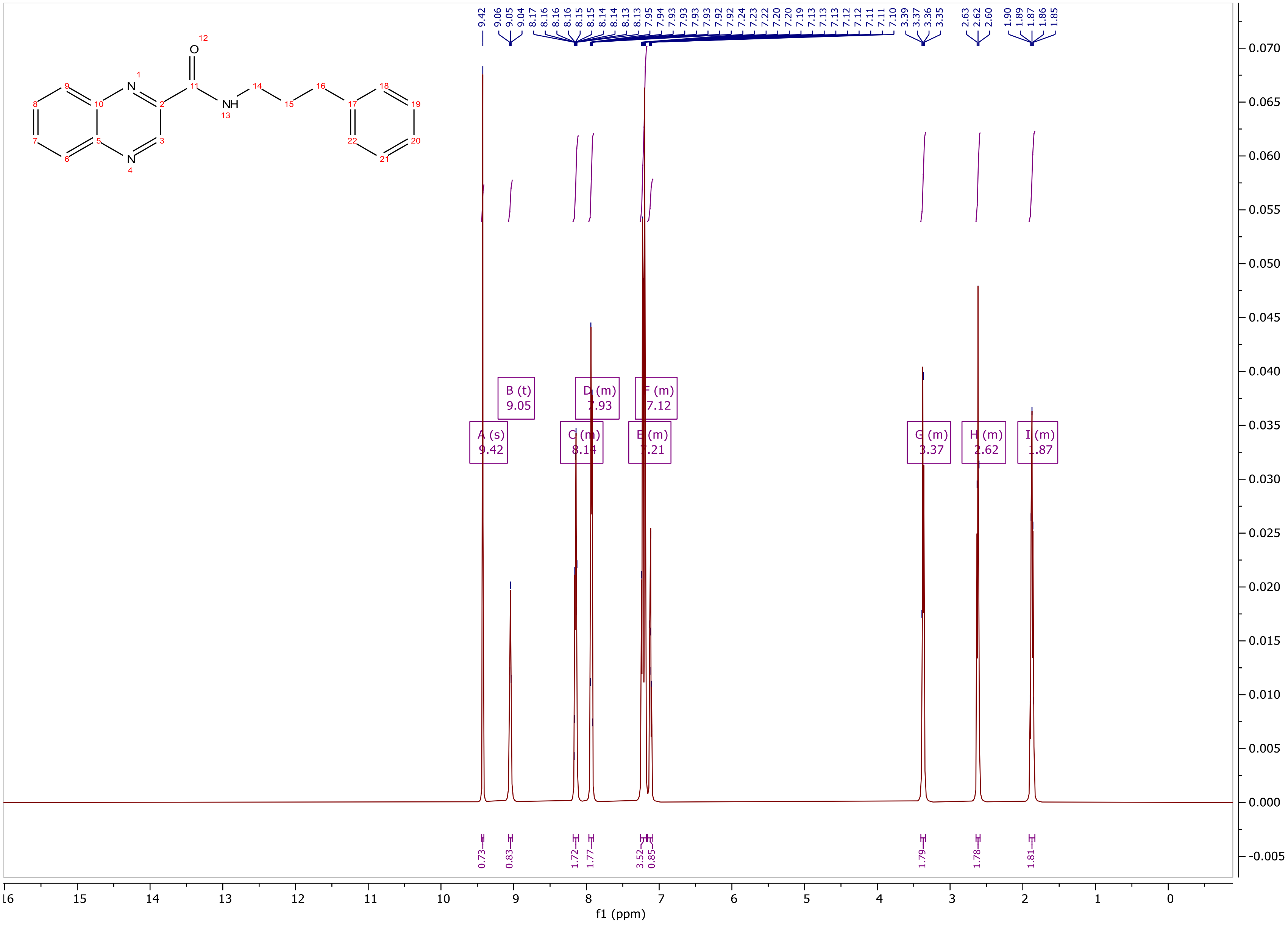

A (s)
9.42
B (t)
9.05
C (m)
8.14
D (m)
7.93
E (m)
7.21
F (m)
7.12
G (m)
3.37
H (m)
2.62
I (m)
1.87
f1 (ppm)

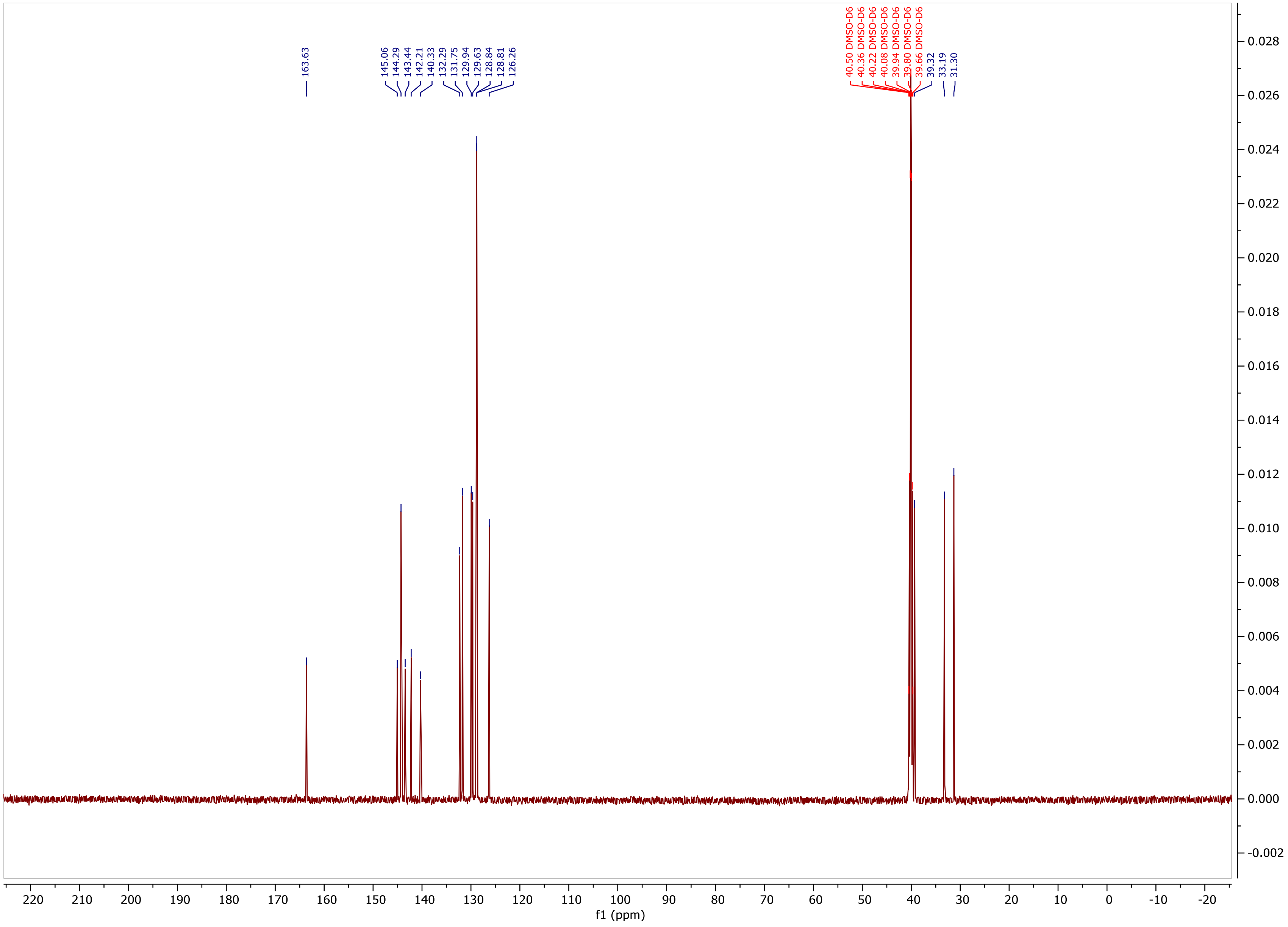
163.63
145.06
144.29
143.44
142.21
140.33
132.29
131.75
129.94
129.63
128.84
128.81
126.26
40.50 DMSO-D6
40.36 DMSO-D6
40.22 DMSO-D6
40.08 DMSO-D6
39.94 DMSO-D6
39.80 DMSO-D6
39.66 DMSO-D6
39.32
33.19
31.30
f1 (ppm)

References

1. Mahesh, R., et al., *Design, synthesis and structure-activity relationship of novel quinoxalin-2-carboxamides as 5-HT3 receptor antagonists for the management of depression.* Bioorganic & Medicinal Chemistry Letters, 2010. **20**(22): p. 6773-6776.
