# Supplementary material for: Synthesis, Biological Evaluation, and In Silico Modeling of N-Substituted Quinoxaline-2-Carboxamides
Source: Pharmaceuticals (Basel). 2021 Aug 4;14(8):768. doi: 10.3390/ph14080768 (PMC8399443; doi:10.3390/ph14080768)
Supplement: Supplementary file 1 [file pharmaceuticals-14-00768-s001.zip › Supplementary Material 2 - Experimental protocols.pdf]

Synthesis, Biological Evaluation and In Silico Modelling of *N*-substituted Quinoxaline-2-carboxamides

Ghada Bouz, Sarah Bouz, Ondřej Janďourek, Klára Konečná, Pavel Bárta, Jarmila Vinšová, Martin Doležal, and Jan Zitko

Faculty of Pharmacy in Hradec Králové, Charles University, Akademika Heyrovského 1203, 50005 Hradec Králové, Czech Republic

Supplementary Materials 2 – Experimental protocols

CONTENTS

1. In vitro antimycobacterial evaluation 1

2. In vitro antibacterial evaluation 2

3. In vitro antifungal evaluation 2

4. In vitro cytotoxicity evaluation 3

References 5

1. In vitro antimycobacterial evaluation

Testing was performed according to a previously published method [1]. Briefly, the antimycobacterial assay was performed with fast-growing *Mycobacterium smegmatis* DSM 43465 (ATCC 607), *Mycobacterium aurum* DSM 43999 (ATCC 23366) from German Collection of Microorganisms and Cell Cultures (Braunschweig, Germany) and with avirulent strain of *Mycobacterium tuberculosis* H37Ra ITM-M006710 (ATCC 9431) from Belgian Co-ordinated Collections of Micro-organisms (Antwerp, Belgium). The technique used for activity determination was microdilution broth panel method using 96-well microtiter plates. The cultivation medium was Middlebrook 7H9 broth (Sigma-Aldrich, Steinheim, Germany) enriched with 0.4% of glycerol (Sigma-Aldrich, Steinheim, Germany) and 10% of Middlebrook OADC growth supplement (Himedia, Mumbai, India). Mycobacterial strains were cultured on Middlebrook 7H9 agar and suspensions were prepared in Middlebrook 7H9 broth. Final density was adjusted to value 1.0 according to McFarland scale and diluted in ratio 1:20 (for fast-growing mycobacteria) or 1:10 (for *M. tuberculosis*) with broth. Tested compounds were dissolved in DMSO (Sigma-Aldrich, Steinheim, Germany), then Middlebrook broth was added to obtain a concentration 2000 µg/mL. Standards used for activity determination were isoniazid (INH), rifampicin (RIF) and ciprofloxacin (CPX) (Sigma-Aldrich, Steinheim, Germany). Final concentrations were reached by binary dilution and addition of mycobacterial suspension and were set as 500, 250, 125, 62.5, 31.25, 15.625, 7.81 and 3.91 µg/mL. Isoniazid was diluted in the range 500–3.91 µg/mL for screening against fast-growing mycobacteria and in the range 1–0.0078 µg/mL for screening against *M. tuberculosis*. Rifampicin final concentrations ranged from 50 to 0.39 µg/mL for fast-growing mycobacteria and from 1 to 0.0078 µg/mL for *M. tuberculosis*. Ciprofloxacin was used for screening antimycobacterial activity with the final concentrations 1–0.0078 µg/mL. The final concentration of DMSO did not exceed 2.5% (v/v) and did not affect the growth of *M. smegmatis*, *M. aurum,* nor *M. tuberculosis*. Positive (broth, DMSO, bacteria) and negative (broth, DMSO) growth controls were included. Plates were sealed with polyester adhesive film and incubated in the dark at 37 °C without agitation. The 0.01% solution of

resazurin sodium salt was added after 48 hours of incubation for *M. smegmatis*, after 72 hours of incubation for *M. aurum* and after 120 hours of incubation for *M. tuberculosis*, respectively. After the addition of the dye, the microtitration plates were further incubated for 2.5 hours for *M. smegmatis*, 4 hours for *M. aurum* and 24 hours for *M. tuberculosis* before the activity was read. The antimycobacterial activity was expressed as minimum inhibitory concentration (MIC) and the value was read based on stain colour change (blue colour – inhibition of growth; pink colour – growth). All experiments were conducted in duplicate.

2. In vitro antibacterial evaluation

Microdilution broth method, according to The European Committee on Antimicrobial Susceptibility Testing (EUCAST) recommendations, with minor modifications, was employed for antibacterial activity evaluation *in vitro* [2]. For the screening of antibacterial activity, eight bacterial strains were included in the study, namely: three reference bacterial strains, *Staphylococcus aureus* CCM 4516 (ATCC 6538), *Escherichia coli* CCM 4517 (ATCC 8739), *Pseudomonas aeruginosa* CCM 1961 (ATCC 9027), obtained from Czech National Collection of Microorganisms Brno, Czech Republic, and five clinical isolate strains, methicillin-resistant *Staphylococcus aureus* H 5996/08 (MRSA), *Staphylococcus epidermidis* H 6966/08, *Enterococcus* sp. J 14365/08, *Klebsiella pneumoniae* D 11750/08, and *Klebsiella pneumoniae* J 14368/08-ESBL (kindly provided from the Department of Clinical Microbiology, University Hospital in Hradec Králové, Czech Republic). The technique used for activity determination was based on a microdilution broth panel method using 96-well microtiter plates. The cultivation was done in Cation-adjusted Mueller-Hinton broth (CAMHB, M-H 2 Broth, Sigma-Aldrich) buffered to pH 7.0 at 35±2 °C. Tested compounds were dissolved in DMSO to produce stock sample solutions. The final concentration of DMSO in the testing medium did not exceed 1% (v/v) of the total sample solution composition. Positive growth controls (microbes without exposure to tested compounds in cultivation medium), negative growth controls (cultivation medium only), and the internal quality standards, gentamicin (GNT) and CIP, were included in assays. All experiments were conducted in duplicates. Antibacterial activity was evaluated after 24 and 48 h of static incubation at 35±2 °C by visual inspection, and expressed as minimum inhibitory concentration (MIC), reported in µM. MICs for standards were as follows; gentamicin [MIC against *Staphylococcus aureus* 1 µg/mL (48 h); *Staphylococcus aureus* methicillin resistant 16–32 µg/mL (48 h); *Enterococcus faecalis* 8 µg/mL (48 h); *Escherichia coli* 1–2 µg/mL (48 h); *Pseudomonas aeruginosa* 0.5 µg/mL (48 h); *Staphylococcus epidermidis* >8 µg/mL (48 h); *Klebsiella pneumonia* >8 µg/mL (48 h); *Serratia marcescens* 2 µg/mL (48 h)] and ciprofloxacin [MIC against *Staphylococcus aureus* 0.128–0.256 µg/mL (48 h); *Staphylococcus aureus* methicillin resistant 0.128 µg/mL (48 h); *Enterococcus faecalis* 0.512 µg/mL (48 h); *Escherichia coli* 0.008 µg/mL (48 h); *Pseudomonas aeruginosa* 0.128 µg/mL (48 h); *Staphylococcus epidermidis* >1.024 µg/mL (48 h); *Klebsiella pneumonia* >1.024 µg/mL (48 h); *Serratia marcescens* 0.256 µg/mL (48 h)].

3. In vitro antifungal evaluation

Microdilution broth method according to EUCAST recommendations, with slight modifications, was employed for antifungal activity screening in vitro [3, 4]. Four yeast reference strains, *Candida albicans* CCM 8320 (ATCC 24433), *Candida krusei* CCM 8271 (ATCC 6258), *Candida parapsilosis* CCM 8260 (ATCC 22019), *Candida tropicalis* CCM 8264 (ATCC 750), and four filamentous fungi, *Aspergillus fumigatus* (ATCC 204305), *Aspergillus flavus* CCM 8363, *Lichtheimia corymbifera* CCM 8077, and *Trichophyton interdigitale* CCM 8377 (ATCC 9533), purchased from the Czech Collection of Microorganisms (CCM, Brno, Czech Republic) or from the American Type Collection Cultures (ATCC, Manassas, VA, USA)], were included in antifungal activity evaluation *in vitro*. The technique used for activity determination was based on microdilution broth panel method with 96-well microtiter plates. The cultivation was done in RPMI-1640 medium, with glutamine and 2% glucose, buffered to pH 7.0 with MOPS (3-morpholinopropane-1-sulfonic acid). Tested compounds were dissolved in DMSO to produce stock

sample solutions. The final concentration of DMSO in the testing medium did not exceed 1% (v/v) of the total sample solution composition. Positive growth controls (microbes without exposure to tested compounds in cultivation medium), negative growth controls (cultivation medium only), and the internal quality standards, amphotericin B (AmB) and voriconazole (VRC), were included in assays. Static incubation was performed in the dark and in a humid atmosphere, at 35±2 °C, for 24 and 48 h (72 and 120 h for *Trichophyton interdigitale*, respectively). All experiments were conducted in duplicates. Antifungal activity of tested compounds was evaluated after visual inspection and expressed as MIC, reported in µM. MICs for standards were as follows; amphotericin B [MIC against *Candida albicans* 0.5 µg/mL (48 h); *C. krusei* 1 µg/mL (48 h); *C. parapsilosis* 0.5 µg/mL (48 h); *C. tropicalis* 1 µg/mL (48 h); *Aspergillus flavus* 8 µg/mL (48 h); *Lichtheimia corymbifera* 0.5 µg/mL (48 h); *Trichophyton interdigitale* 2 µg/mL (72 h); *Aspergillus fumigatus* 1 µg/mL (48 h)] and voriconazole [MIC against *Candida albicans* >16 µg/mL (48 h); *C. krusei* 0.5 µg/mL (48 h); *C. parapsilosis* 8 µg/mL (48 h); *C. tropicalis* >16 µg/mL (48 h); *Aspergillus flavus* >16 µg/mL (48 h); *Lichtheimia corymbifera* >16 µg/mL (48 h); *Trichophyton interdigitale* >16 µg/mL (72 h); *Aspergillus fumigatus* 1 µg/mL (48 h)].

4. In vitro cytotoxicity evaluation

The Human Caucasian hepatocyte carcinoma cell line HepG2, obtained from the European Collection of Authenticated Cell Cultures (ECACC, Catalogue No.: 85011430), was cultured in MEM (Minimum Essentials Eagle Medium, Sigma–Aldrich) supplemented with 10% fetal bovine serum (PAA Laboratories, Pasching, Austria), 1% L-glutamine solution (Sigma–Aldrich) and non-essential amino acid solution (Sigma–Aldrich). Cell culture was grown in a humidified atmosphere containing 5% CO_2 at 37 °C. For subculturing, the cells were harvested after trypsin/EDTA (Sigma–Aldrich) treatment at 37 °C. To evaluate cytotoxicity, the cells treated with the tested substances were used as experimental groups, whereas untreated HepG2 cells served as controls. The cells were seeded in density 10,000 cells per well in a 96 well plate. The next day, the cells were treated with each of the tested substances dissolved in DMSO. The tested substances were prepared at different incubation concentrations in triplicates according to their solubility. Simultaneously, the controls representing 100% cell viability (the cells treated with 1% DMSO), 0% cell viability (the cells treated with 10% DMSO), no cell control and vehiculum controls were also prepared in triplicates. After 24 h incubation in a humidified atmosphere containing 5% CO_2 at 37%, the reagent from the kit CellTiter 96 AQueous One Solution Cell Proliferation Assay (CellTiter 96; PROMEGA, Fitchburg, USA) was added. After 2h incubation at 37 °C absorbance of samples was recorded at 490 nm (TECAN, Infinita M200, Austria). A standard toxicological parameter IC_{50} was calculated by nonlinear regression from a semilogarithmic plot of incubation concentration versus percentage of absorbance relative to untreated controls using GraphPad Prism 9 software. Tamoxifen was used as standard with IC_{50} value equal to 19.56 µM.

The human epithelial kidney carcinoma A498 cell line, obtained from American Type Culture Collection (ATCC, Manassas, VA, USA, catalogue No.: HTB-44), was cultured in RPMI medium, the kidney cortex proximal tubule HK-2 cell line, obtained from ATCC (Manassas, VA, USA, catalogue No.: CRL-2190) was cultured in DMEM High Glucose medium, Human Caucasian prostate adenocarcinoma PC-3 cell line, obtained from ATCC (Manassas, VA, USA, catalogue No.: 90112714), was cultured in a Nutrient mixture F12 Ham medium; Human Caucasian ovary adenocarcinoma SK-OV-3 cell line, obtained from ATCC, (Manassas, VA, USA, catalogue No.: 91091004), was cultured in McCoy's 5a medium, and Human glioblastoma astrocytoma U-87 MG cell line, obtained from ECACC (catalogue No.: 89081402) was cultured in DMEM High Glucose medium. All cultures were supplemented with 10% fetal bovine serum, 1% non-essential amino acids (A498 and U-87 MG only), 1% sodium pyruvate (A498 and U-87 MG only) and 2 mM L-glutamine. All cell culture consumables were purchased from Merck (NJ, USA). The cells were kept at 37 °C in a 5% carbon dioxide humidified incubator and were subcultured and used for experiments at a confluency of 70–90%. On the day of the experiment, cells were seeded in 96-well plate in the number 10 000 cells per well in the cell culture medium and left to incubate for 24 hours. The tested compound was added in the chosen concentrations (0.0001, 1, 5, 10, 25, 50, 100,

250, 500 and 1 000 µM in triplicates), and cells were incubated for the next 24 hours. Eventually, a colourimetric assay reagent was added, and absorption was measured.

HepG2 IC_{50} curves for our most active compounds ($MIC_{MtbH37Ra} \leq 7.81$ µg/mL) in addition to compound **29** (studied for its cytotoxic effects) are shown in Figure S1.

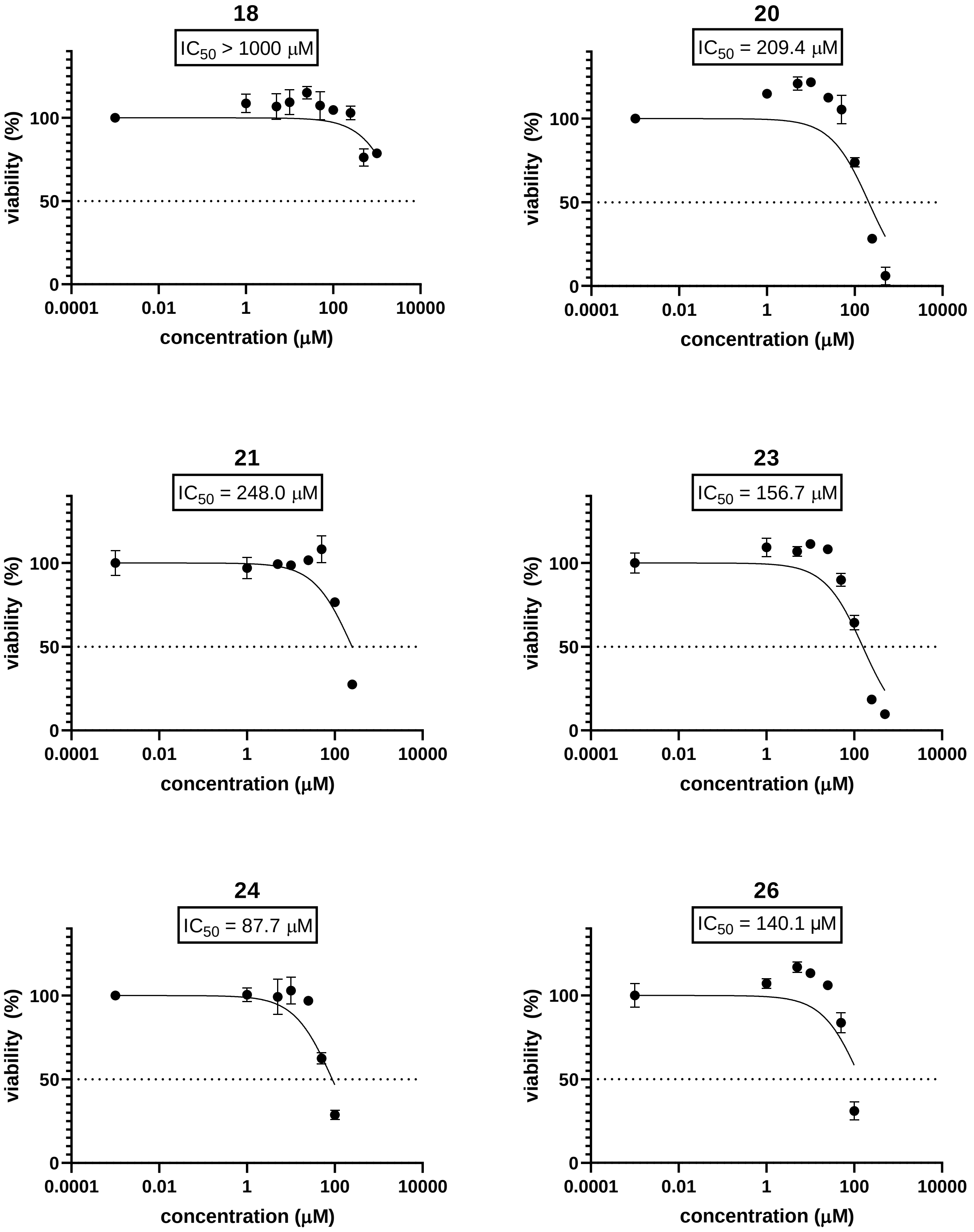

Figure S1. HepG2 IC_{50} curves of selected compounds.

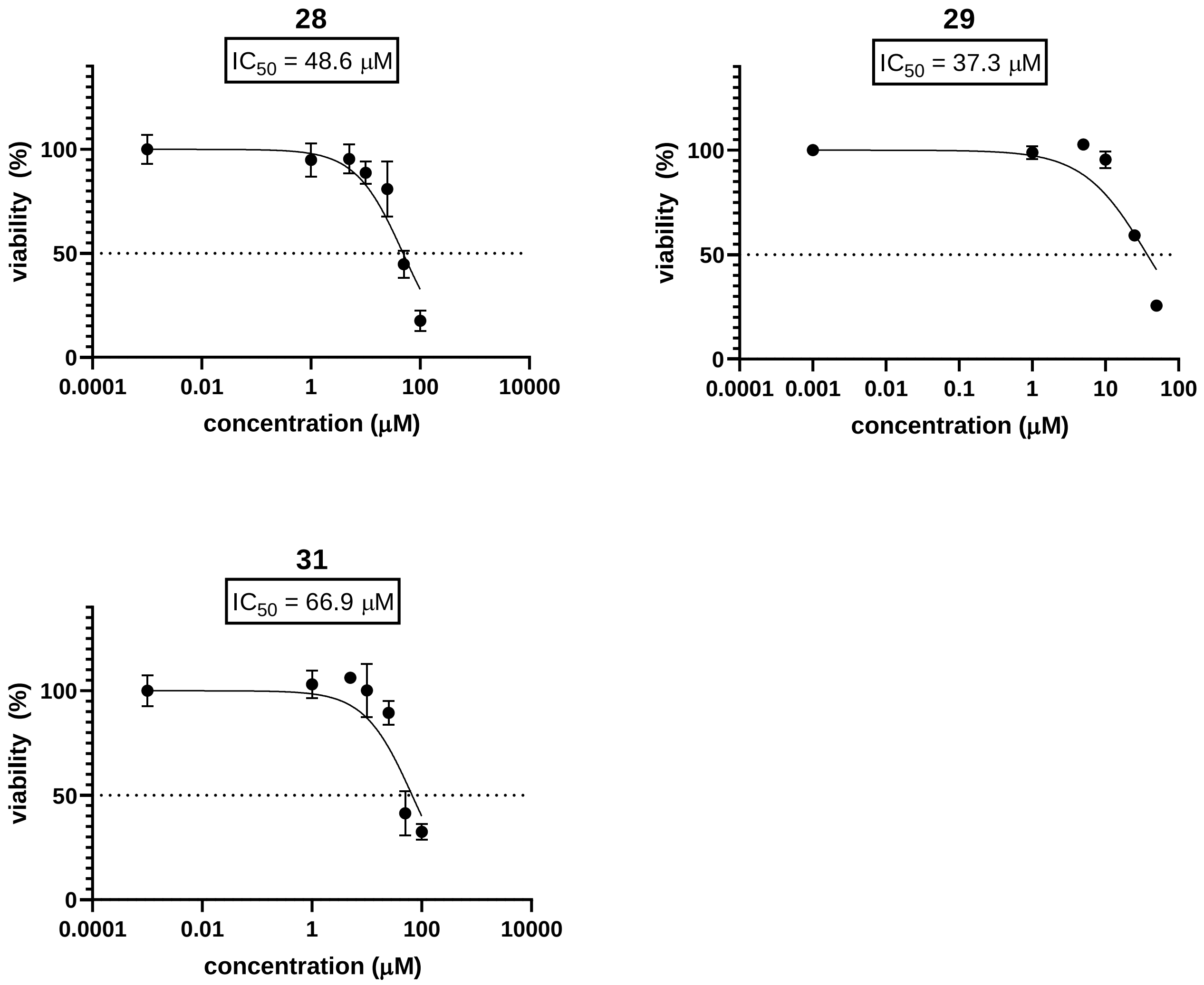

Figure S1. HepG2 IC_{50} curves of selected compounds (continued).

References

1. Franzblau, S.G.; Witzig, R.S.; McLaughlin, J.C.; Torres, P.; Madico, G.; Hernandez, A.; Degnan, M.T.; Cook, M.B.; Quenzer, V.K.; Ferguson, R.M.; et al. Rapid, low-technology MIC determination with clinical Mycobacterium tuberculosis isolates by using the microplate Alamar Blue assay. *J. Clin. Microbiol.* **1998**, *36*, 362–366.
2. European Committee for Antimicrobial Susceptibility Testing (EUCAST) of the European Society of Clinical Microbiology and Infectious Diseases (ESCMID). EUCAST DISCUSSION DOCUMENT E.Dis 5.1. Determination of minimum inhibitory concentrations (MICs) of antibacterial agents by broth dilution. *Clin. Microbiol. Infect.* **2003**, *9*, 1–7. doi: 10.1046/j.1469-0691.2003.00790.x.
3. Arendrup, M.C.; Meletiadis, J.; Mouton, J.W.; Lagrou, K.; Hamal, P.; Guinea, J.; Subcommittee on Antifungal Susceptibility Testing (AFST) of the ESCMID European Committee for Antimicrobial Susceptibility Testing (EUCAST). Method for the determination of broth dilution minimum inhibitory concentrations of antifungal agents for yeasts. EUCAST Definitive Document E.Def 7.3.1. 2017. Available online: http://www.eucast.org/astoffungi/methodsinantifungalsusceptibilitytesting/susceptibility_testing_of_yeasts/ (accessed on 5 March 2020).

4. Arendrup, M.C.; Meletiadis, J.; Mouton, J.W.; Lagrou, K.; Hamal, P.; Guinea, J.; Subcommittee on Antifungal Susceptibility Testing (AFST) of the ESCMID European Committee for Antimicrobial Susceptibility Testing (EUCAST). Method for the determination of broth dilution minimum inhibitory concentrations of antifungal agents for conidia forming moulds. EUCAST Definitive Document E.Def 9.3.1. 2017. Available online: http://www.eucast.org/astoffungi/methodsinantifungalsusceptibilitytesting/susceptibility_testing_of_moulds/ (accessed on 5 March 2020).
