# Supplementary material for: Synthesis, Biological Evaluation, and In Silico Modeling of N-Substituted Quinoxaline-2-Carboxamides
Source: Pharmaceuticals (Basel). 2021 Aug 4;14(8):768. doi: 10.3390/ph14080768 (PMC8399443; doi:10.3390/ph14080768)
Supplement: Supplementary file 1 [file pharmaceuticals-14-00768-s001.zip › Supplementary Material 3 - In silico studies.pdf]

Synthesis, Biological Evaluation and In Silico Modelling of *N*-substituted Quinoxaline-2-carboxamides

Ghada Bouz, Sarah Bouz, Ondřej Janďourek, Klára Konečná, Pavel Bárta, Jarmila Vinšová, Martin Doležal, and Jan Zitko

Faculty of Pharmacy in Hradec Králové, Charles University, Akademika Heyrovského 1203, 50005 Hradec Králové, Czech Republic

Supplementary Material 3 – *In silico* modelling

CONTENTS

1. Experimental 1

1.1. General 1

1.2. Preparation of ligands 1

1.3. Docking to DprE1 1

1.4. Docking to selected anticancer targets 2

1.5. Docking to human protein kinases 2

1.6. Docking to human VEGFR2 3

2. Additional results 3

2.1. Docking to selected anticancer targets 3

2.2. Docking to human protein kinases 4

2.3. Docking to human VEGFR2 4

REFERENCES 4

1. Experimental

1.1. General

In silico calculations were performed in Molecular Operating Environment (MOE) 2020.09 (Chemical Computing Group Inc., Montreal, QC, Canada) under Amber10:EHT forcefield. Figures were prepared using MOE.

1.2. Preparation of ligands

The ligands for docking were generated from SMILES, using the dominant protomer at pH 7. The abundance of the dominant protomer was >99% for all compounds. 3D coordinates were minimized until RMS gradient 0.01 kcal.mol^{-1}.Å^{-1}.

1.3. Docking to DprE1

3D coordinates of mycobacterial decaprenylphosphoryl-β-D-ribose oxidase (DprE1) were downloaded from the PDB database (PDB ID: 4P8N). For Trp230, the altA coordinates were chosen from the two mutually alternative sets of coordinates written in the original PDB file with equal fractional occupancy

(0.5). In the altA set, the sidechain of Trp230 is closer to the co-crystalized ligand and forms a tighter binding place, therefore more accustomed to the ligand. The protein was prepared by MOE QuickPrep functionality with default settings, which included corrections of structural errors, the addition of hydrogens, calculation of partial charges, 3D optimization of protonation/tautomeric states and *H*-bond network (Protonate3D), deletion of water molecules further than 4.5 Å from ligand or protein, and a restrained minimization (to RMS gradient of 0.01 kcal.mol^{-1}.Å^{-1}) of ligand and pocket residues within 8 Å from the ligand. Subsequently, all solvent molecules were removed, and the FAD co-factor was defined as a part of the protein.

The docking was focused on the pocket, which is defined as a set of residues having at least one atom within 4.5 Å from the co-crystalized ligand. Parameters of the MOE docking protocol: Docking stage – Placement method: Triangle Matcher; Score: London dG; retain 30 poses. Refinement stage – Rigid receptor; Score: GBVI/WSA dG; retain 5 poses. Ligand conformations – Rotate bonds.

1.4. Docking to selected anticancer targets

We performed molecular docking of compound **29** into human targets selected based on the review article.[1] The selection from the PDB database was made manually, considering the structure quality criteria and structural similarity of the co-crystalized ligand to compound **29**. The full list of studied targets with resulting docking scores may be found in Table S1.

The coordinates of selected targets were downloaded from the PDB database. After manual inspection, the ligand was defined, and co-factors (if present) were redefined as a part of the receptor. In the case of protein multimers, chain A was used. All structures were subjected to the QuickPrep procedure described above. Water molecules were disregarded in the docking calculation. The docking was focused on the pocket, which is defined as a set of residues having at least one atom within 4.5 Å from the co-crystalized ligand. The docking was performed as induced-fit docking, assigning partial flexibility to side chains of the pocket residues. Parameters of the MOE docking protocol: Docking stage – Placement method: Triangle Matcher; Score: London dG; retain 30 poses. Refinement stage – Induced fit (Side Chains: Tether); Score: GBVI/WSA dG; retain 5 poses. Ligand conformations – Rotate bonds.

1.5. Docking to human protein kinases

The manually curated Kinase Database (8661 structures) distributed with MOE 2020.09 was searched for human protein kinases which contained a ligand with close structural similarity to title compound **29** (compared via Bit-Packed MACCS Structural Keys as molecular fingerprints, with Tanimoto index ≥ 0.6). The search yielded **40 structures** with unique PDB codes. The PDB coordinates (as present in the curated database) of the selected structures were imported into a database and submitted (in a batch mode) to the QuickPrep procedure described above. The docking was focused on the pocket, which is defined as a set of residues having at least one atom within 4.5 Å from the co-crystalized ligand. The docking was performed as induced-fit docking, using the same protocol as in section 1.4. The results are presented in Table S2.

List of studied structures (40 structures with unique PDB code) of human protein kinases extracted from the MOE Kinase Database (ordered alphabetically):

1ZYJ; 2BRH; 2JBO; 2JBP; 2P2I; 3E87; 3FHR; 3GOK; 3HNG; 3NUU; 3NUY; 3R1Y; 3R2Y; 4AFJ; 4BDC; 4BDK; 4DIT; 4EH6; 4F9B; 4FT9; 4J71; 4JAJ; 4JG7; 4ZLY; 4ZLZ; 5EOL; 5EW3; 5IH8; 5L4Q; 5L6P; 5MZL; 5NDT; 5TIU; 6CJE; 6E9W; 6ED6; 6FT7; 6GI6; 6HK6; 7JNT (chain A used for all entries)

1.6. Docking to human VEGFR-2

The Kinase Database was searched for the term "vascular endothelial growth factor" – (57 entries); filtered to Family "VEGFR" – (53 entries); filtered to human – (52 entries); and filtered by crystallographic resolution ≤ 2.5 Å (**33 entries** with unique PDB code). All retrieved structures were VEGFR-2. The PDB coordinates (as present in the curated database) of the selected structures were imported into a database and submitted (in a batch mode) to the QuickPrep procedure described above. The docking was focused on the pocket, which is defined as a set of residues having at least one atom within 4.5 Å from the co-crystalized ligand. The docking was performed as induced-fit docking, using the same protocol as in section 1.4. The results are presented in Table S3.

List of studied structures (33 structures with unique PDB code) of human VEGFR-2 extracted from the MOE Kinase Database (ordered alphabetically):

1Y6A; 1Y6B; 1YWN; 2OH4; 2P2H; 2P2I; 2QU6; 3BE2; 3C7Q; 3CJF; 3CJG; 3CP9; 3CPC; 3EFL; 3EWH; 3U6J; 3VHE; 3VHK; 3VID; 3VNT; 3VO3; 3WZD; 3WZE; 4AG8; 4AGC; 4ASD; 4ASE; 5EW3; 6GQO; 6GQP; 6GQQ; 6XVJ; 6XVK (chain A used in all entries)

2. Additional results

2.1. Docking to selected anticancer targets

The overview of PDB structures studied as potential targets for cytostatic activity of compound **29** by molecular docking can be found in Table S1. Docking score (S) is the docking score for the best pose of compound **29** in each receptor.

Table S1. Results of molecular docking of compound **29** to selected human targets (ordered by docking score)

PDB ID	Name	UniProt ID	Docking Score
1K4T	Human DNA topoisomerase/DNA	P11387	-8.284[a]
6JQR	Fms-like tyrosine kinase 3	P36888	-8.079
6VWC	Bcl-2-like protein 1	Q07817	-8.065
5MY8	Serine/arginine-protein kinase 1	Q96SB4	-7.514
5LQF	Cyclin-dependent kinase 1	P06493	-7.279
3R04	Proto-oncogene serine/threonine-protein kinase pim-1	P11309	-7.156
1ZXN	DNA topoisomerase II, alpha isozyme	P11388	-7.074
6INL	Cyclin-dependent kinase 2	P24941	-6.997
2WXF	PI3-kinase subunit delta	O35904	-6.959
2IEJ	Human Protein Farnesyltransferase	P49354	-6.601
6QGH	Bcl-2-like protein 1	Q07817	-6.229

[a] The pose is depicted in Figure 5 in the main article.

2.2. Docking to human protein kinases

Table S2 presents the results of induced fit molecular docking of compound **29** into human kinases selected based on structural similarity of co-crystalized ligand to compound **29**. The poses of compound **29** with *cis*-carboxamide configuration were omitted.

Table S2. Top 10 non-redundant human kinases as potential targets for compound **29**

Entry	PDB ID	Name	UniprotID	Score
1	5EW3.A	VEGFR2 kinase domain	P35968	-8.127
2	3HNG.A	VEGFR1 kinase domain	P17948	-8.017
3	2P2I.A	VEGFR2 kinase domain	P35968	-7.657
4	4JG7.A	RSK2 CTD	P51812	-7.561
5	6GI6.A	ACVR1 (ALK2) kinase	Q04771	-7.512
6	5L6P.A	EphB3 kinase domain	P54753	-7.475
7	7JNT.A	Rho-associated protein kinase 2 (Rock2)	O75116	-7.381
8	3E87.A	kinase domain of AKT2	P31751	-7.362
9	6ED6.A	Rock2	O75116	-7.325
10	2JBP.A	Protein kinase MK2	P49137	-7.322
11	4ZLZ.A	Bruton's Tyrosine Kinase	Q06187	-7.295
12	6E9W.A	Rock1	Q13464	-7.283
13	4FT9.A	CHK1	O14757	-7.250

Colouring of the cells denotes isoforms of the same kinase (white background cells were not categorized).

2.3. Docking to human VEGFR2

Table S3. Top 10 scoring structures of human VEGFR2 after docking of compound **29** [a]

Rank	PDB ID	Score
1	4ASD.A	-8.368
2	6XVJ.A	-8.336
3	4AGC.A	-8.336
4	4AG8.A	-8.320[b]
5	3VO3.A	-8.289
6	3VNT.A	-8.232
7	2QU6.A	-8.065
8	6GQO.A	-8.060
9	3WZE.A	-8.042
10	6XVK.A	-8.035

[a] Colouring of the cells aggregates the same binding mode (white background cells were not categorized). [b] The pose is depicted in Figure 6 in the main paper.

REFERENCES

[1] T. Kaushal, G. Srivastava, A. Sharma, A. Singh Negi, An insight into medicinal chemistry of anticancer quinoxalines, Bioorganic & Medicinal Chemistry, 27 (2019) 16-35. https://doi.org/https://doi.org/10.1016/j.bmc.2018.11.021
